# Supplementary material for: Systematic morphological profiling of human gene and allele function via Cell Painting
Source: eLife. 2017 Mar 18;6:e24060. doi: 10.7554/eLife.24060 (PMC5386591; doi:10.7554/eLife.24060)
Supplement: Supplementary file 1. — (A) List of all the 323 constructs used in the experiment along with the target transcript and their public clone ID. (B) Replicate correlation is higher in the constitutively active mutant allele compared to the wild-type allele, except for AKT3_E17K. Constitutively active mutant annotations were obtained by literature search for all the mutants in the experiment showing a detectable phenotype. Genes shown here are only those where either the wild-type gene or its constitutively activating allele yielded a phenotype distinct from controls. (C) Pathways sorted based on proportion of their associated gene showing a detectable phenotype. (D) Highly correlated proteins (according to morphology in the Cell Painting assay) that have also been reported to interact physically. (E) Highly correlated genes (according to morphology in the Cell Painting assay) that have also been annotated to be related to the same pathway. (F) Gene Ontology terms associated with each gene cluster (Alexa and Rahnenführer, 2009). (G) Rank ordered list of distinctive features based on their z-scores for Cluster 19. (H): All genes/alleles in Cluster 8 and 10 induce cell rounding. (I) The NF-κB signaling pathway is the most enriched when searching for gene overexpressions that downregulate known YAP/TAZ targets (CYR61, CTGF, and BIRC5). DOI: http://dx.doi.org/10.7554/eLife.24060.016 [file elife-24060-supp1.zip › Supp_Files/1A - List of constructs.pdf]

A: List of all the 323 constructs used in the experiment along with the target transcript and their public clone ID.

Assigned Treatment Name	Target Transcript	Public clone ID
ACVR1B_WT.1	BC000254.1	ccsbBroad304_14528
ACVR1B_WT.2.mismatch	NM_001106.3	BRDN0000464885
ACVR1B_WT.3	BC000254.1	ccsbBroad304_00018
ADAM17_WT	BC062687.1	ccsbBroad304_11169
AKT1_E17K	AKT1_c.49G>A	BRDN0000464992
AKT1_WT.1	BC000479.2	ccsbBroad304_14538
AKT1_WT.2	BC000479.2	ccsbBroad304_00046
AKT1S1_WT.1	NM_001098633.3	BRDN0000464862
AKT1S1_WT.2	NM_001098633.3	BRDN0000464863
AKT2_WT	BC120994.1	ccsbBroad304_00047
AKT3_E17K	NM_181690.2:c.49G>A	BRDN0000464970
AKT3_WT.1	BC121154.2	ccsbBroad304_07529
AKT3_WT.2	NM_005465.4	BRDN0000464937
AKT3_WT.3	NM_005465.3	ccsbBroad304_14950
APAF1_WT	NM_181861.1	BRDN0000464892
APC_WT	NM_001127510.2	BRDN0000464853
ARAF_WT.1	NM_001654.4	BRDN0000464899
ARAF_WT.2	BC002466.2	ccsbBroad304_14544
ARNTL_WT	BC041129.1	ccsbBroad304_00105
ATF2_WT	BC130335.1	ccsbBroad304_00361
ATF4_WT.1	BC016855.2	ccsbBroad304_00117
ATF4_WT.2	NM_182810.1	BRDN0000464922
ATF6_1-373	NM_007348.3	BRDN0000464973
ATF6_WT.1	BC014969.1	ccsbBroad304_11651
ATF6_WT.2	NM_007348.3	BRDN0000464861
ATG16L1_WT	BC117337.1	ccsbBroad304_03512
ATG5_WT	BC002699.2	ccsbBroad304_02173
ATM_WT.1	BC061584.1	ccsbBroad304_10688
ATM_WT.2	BC022307.1	ccsbBroad304_10689
AXIN2_WT.1	BC101533.1	ccsbBroad304_07217

AXIN2_WT.2	NM_004655.3	BRDN0000464927
BAMBI_WT	BC019252.1	ccsbBroad304_02857
BAX_WT	BC014175.2	ccsbBroad304_00150
BCL2L1_WT	BC019307.1	ccsbBroad304_00156
BCL2L11_WT	BC033694.1	ccsbBroad304_02292
BECN1_WT	NM_003766.3	BRDN0000464916
BMP2_WT	NM_001200.2	BRDN0000464919
BMPR1B_K231R	NM_001256794.1:c.692A>G	BRDN0000464959
BMPR1B_Q203D	NM_001256794.1:c.607CAG>GAC	BRDN0000464971
BMPR1B_WT.1	BC047773.1	ccsbBroad304_00168
BMPR1B_WT.2	BC008718.2	ccsbBroad304_05836
BRAF_V600E	NM_004333.4	BRDN0000464987
BRAF_WT.1	NM_004333.4	BRDN0000464953
BRAF_WT.2	NM_004333.4	BRDN0000464954
BRCA1_WT	BC072418.1	ccsbBroad304_00173
BTRC_WT	BC027994.1	ccsbBroad304_02053
CARD11_F123I.mismatch	NM_032415.4	BRDN0000464978
CARD11_L225LI	NM_032415.4:c.676insATT	BRDN0000464979
CARD11_WT	NM_032415.2	ccsbBroad304_15196
CASP8_WT.1	BC068050.1	ccsbBroad304_10710
CASP8_WT.2	NM_033355.3	BRDN0000464929
CASP9_WT	BC002452.2	ccsbBroad304_00223
CCND1_WT.1	BC000076.2	ccsbBroad304_00155
CCND1_WT.2	BC023620.2	ccsbBroad304_05880
CCNE1_WT.mismatch	BC035498.2	ccsbBroad304_00238
CDC42_Q61L	NM_001791.3:c.182A>T	BRDN0000464980
CDC42_T17N	NM_001791.3:c.50CA>AC	BRDN0000464972
CDC42_WT	NM_001791.3	BRDN0000464907
CDK2_WT.1	BC003065.2	ccsbBroad304_00276
CDK2_WT.2	BC003065.2	ccsbBroad304_14572
CDK4_R24C	NM_000075.3:c.70C>T	BRDN0000464958
CDK4_WT.1	BC003644.2	ccsbBroad304_00277
CDK4_WT.2	BC003644.2	ccsbBroad304_14574
CDKN1A_WT	BC000275.1	ccsbBroad304_00282

CEBPA_WT.1	NM_004364.3	BRDN0000464846
CEBPA_WT.2	NM_004364.3	BRDN0000464847
CHUK_WT.1.mismatch	NM_001278.3	BRDN0000464881
CHUK_WT.2	NM_001278.3	BRDN0000464884
CLOCK_WT	BC126157.1	ccsbBroad304_02201
CREB1_WT	BC010636.1	ccsbBroad304_00360
CREBBP_WT	NM_004380.2	BRDN0000464848
CRY1_WT.1	NM_004075.3	BRDN0000464909
CRY1_WT.2	BC030519.1	ccsbBroad304_06040
CSNK1A1_WT.1	BC008717.2	ccsbBroad304_06055
CSNK1A1_WT.2	NM_001892.3	ccsbBroad304_14599
CSNK1A1_WT.3	NM_001892.4	BRDN0000464855
CSNK1E_WT.1	BC006490.2	ccsbBroad304_00379
CSNK1E_WT.2	BC006490.2	ccsbBroad304_14601
CTNNB1_S33A.S37A.T41A.T45A	NM_001904.3:c.97TCTGGAATCCATTCTGGTGCCACTACCACAGCTCCTTCT>GCTGGAATCCATGCTGGTGCCACTGCCACAGCTCCTGCT	BRDN0000464976
CTNNB1_WT	NM_001904.3	BRDN0000464854
CXXC4_WT	BC119751.1	ccsbBroad304_04207
CYLD_WT	NM_001042412.1	BRDN0000464943
DDIT3_WT.1	NM_004083.5	BRDN0000464925
DDIT3_WT.2	BC003637.2	ccsbBroad304_06089
DDIT4_WT	BC007714.1	ccsbBroad304_03433
DEPTOR_WT.1	NM_022783.2	BRDN0000464870
DEPTOR_WT.2	NM_022783.2	BRDN0000464871
DIABLO_WT	BC011909.2	ccsbBroad304_03728
DKK1_WT	BC001539.2	ccsbBroad304_02706
DLL1_WT	NM_005618.3	BRDN0000464866
DUSP1_WT.mismatch	NM_004417.3	BRDN0000464923
DVL1_WT.1	BC050454.1	ccsbBroad304_10790
DVL1_WT.2	NM_004421.2	BRDN0000464888
DVL2_WT	BC014844.1	ccsbBroad304_00470
DVL3_WT	BC032459.1	ccsbBroad304_06132

E2F1_WT	NM_005225.2	BRDN0000464915
EGLN1_WT.1.mismatch	BC005369.1	ccsbBroad304_12062
EGLN1_WT.2	NM_022051.2	BRDN0000464947
EGLN1_WT.3	NM_022051.2	BRDN0000464948
EIF2A_WT.1	NM_032025.3	BRDN0000464896
EIF2A_WT.2	NM_032025.3	BRDN0000464891
EIF4E_WT.1	NM_001968.3	BRDN0000464904
EIF4E_WT.2	BC012611.1	ccsbBroad304_06150
EIF4EBP1_WT	BC004459.1	ccsbBroad304_00490
ELK1_WT	BC056150.1	ccsbBroad304_00498
ERBB2_WT.2	NM_004448.2	BRDN0000464849
ERG_truncated	NM_182918.3:c.1_96del	BRDN0000464994
ERN1_WT.1	NM_001433.3	BRDN0000464913
ERN1_WT.2	BC130405.1	ccsbBroad304_14634
FGFR3_K650E	FGFR3_c.1947A>G	BRDN0000464993
FH_WT	BC003108.1	ccsbBroad304_00565
FOXO1_WT.1	NM_002015.3	BRDN0000464875
FOXO1_WT.2	NM_002015.3	BRDN0000464876
FURIN_WT.1	NM_002569.2	BRDN0000464932
FURIN_WT.2	BC012181.1	ccsbBroad304_06683
GLI1_WT	NM_005269.2	BRDN0000464869
GRB10_WT.1	NM_005311.4	BRDN0000464921
GRB10_WT.2	NM_005311.4	BRDN0000464936
GSK3B_WT.1	NM_002093.3	BRDN0000464897
GSK3B_WT.2	NM_002093.3	BRDN0000464903
HIF1A_WT.1	NM_001530.3	BRDN0000464910
HIF1A_WT.2	BC012527.2	ccsbBroad304_06365
HIF1AN_WT	BC007719.2	ccsbBroad304_03625
HRAS_G12V	HRAS_c.35G>T	BRDN0000464990
HSP90AA1_WT	CCSB53186.1	ccsbBroad304_06413
HSP90B1_WT.1	NM_003299.1	BRDN0000464924
HSP90B1_WT.2	NM_003299.1	BRDN0000464938
HSPA5_WT	NM_005347.4	BRDN0000464901
IKBKB_WT	BC108694.1	ccsbBroad304_00841

IKBKE_WT.1	BC107812.1	ccsbBroad304_02218
IKBKE_WT.2	NM_014002.2	ccsbBroad304_14943
IRAK1_WT	NM_001025243.1	BRDN0000464931
IRAK4_WT	BC013316.1	ccsbBroad304_08230
IRGM_WT.1	BC128168.1	ccsbBroad304_13609
IRGM_WT.2	NM_001145805.1	BRDN0000464864
IRS1_WT	BC053895.1	ccsbBroad304_00882
JAG1_WT	NM_000214.2	BRDN0000464934
JAK2_V617F.mismatch	NM_004972.3	BRDN0000464967
JAK2_WT	NM_004972.3	ccsbBroad304_14680
JUN_WT.1	BC006175.1	ccsbBroad304_14682
JUN_WT.2	NM_002228.3	BRDN0000464926
KRAS_G12V	NM_033360.2	BRDN0000464989
KRAS_WT.1	NM_004985.3	BRDN0000464850
KRAS_WT.2	NM_004985.3	BRDN0000464851
LRPPRC_WT	NM_133259.2	ccsbBroad304_14956
MAP2K1_WT.1	NM_002755.3	ccsbBroad304_14807
MAP2K1_WT.2	NM_002755.3	BRDN0000464894
MAP2K3_WT	NM_145109.2	BRDN0000464945
MAP2K4_WT.1	NM_003010.2	BRDN0000464935
MAP2K4_WT.2	NM_003010.2	BRDN0000464893
MAP3K11_WT	NM_002419.2	ccsbBroad304_14698
MAP3K2_WT.1	NM_006609.4	BRDN0000464898
MAP3K2_WT.2	NM_006609.3	ccsbBroad304_14974
MAP3K5_WT	BC054503.2	ccsbBroad304_14695
MAP3K7_WT	BC017715.2	ccsbBroad304_14856
MAP3K8_WT	BC113566.1	ccsbBroad304_00348
MAP3K9_WT	NM_033141.2	BRDN0000464882
MAPK1_WT.1	NM_138957.2	BRDN0000464917
MAPK1_WT.2	NM_138957.2	ccsbBroad304_14799
MAPK13_WT.1	BC000433.2	ccsbBroad304_01289
MAPK13_WT.2	BC000433.2	ccsbBroad304_14806
MAPK14_WT.1	BC000092.1	ccsbBroad304_00371
MAPK14_WT.2	BC000092.1	ccsbBroad304_14596

MAPK3_WT	NM_002746.2	ccsbBroad304_14800
MAPK7_WT.mismatch	NM_139034.2	BRDN0000464930
MAPK8_WT.1	BC130570.1	ccsbBroad304_01287
MAPK8_WT.2	NM_139047.1	ccsbBroad304_14803
MAPK9_WT.1	BC032539.1	ccsbBroad304_01288
MAPK9_WT.2	BC032539.1	ccsbBroad304_14804
MAPKAP1_WT	NM_024117.3	BRDN0000464941
MCL1_WT	BC017197.2	ccsbBroad304_00985
MEK1_S218D.S222D.mismatch	NM_002755.3:c.652TCCATGGCCAACTCC>GACATGGCCAACGAC	BRDN0000464991
MKNK1_WT	NM_003684.4	ccsbBroad304_14905
MLST8_WT	BC001313.2	ccsbBroad304_03937
MOS_WT.1	BC069569.1	ccsbBroad304_01029
MOS_WT.2	BC069569.1	ccsbBroad304_14699
MYD88_L265P	NM_002468.4:c.795G>A	BRDN0000464966
MYD88_WT	NM_002468.4	BRDN0000464874
NFKB1_WT.1	NM_003998.3	BRDN0000464933
NFKB1_WT.2	BC051765.1	ccsbBroad304_06637
NFKB2_WT	NM_001077494.2	BRDN0000464911
NFKBIA_WT	BC002601.2	ccsbBroad304_01093
NFKBIB_WT	BC015528.1	ccsbBroad304_01094
NFKBIE_WT	NM_004556.2	BRDN0000464920
NOTCH1_ICN1.1	NM_017617.3:c.4_5280del	BRDN0000464996
NOTCH1_WT.1	NM_017617.3	BRDN0000464955
NOTCH1_WT.2	NM_017617.3	BRDN0000464956
NOTCH2_WT	BC071562.1	ccsbBroad304_11001
PAK1_WT.1	BC109299.1	ccsbBroad304_01144
PAK1_WT.2	NM_002576.4	ccsbBroad304_14726
PDPK1_WT.1	BC006339.2	ccsbBroad304_01168
PDPK1_WT.2	BC006339.2	ccsbBroad304_14741
PER1_WT.1	BC028207.1	ccsbBroad304_11025
PER1_WT.2	NM_002616.2	BRDN0000464860
PHLPP1_WT	NM_194449.3	BRDN0000464946
PIK3CA_WT.1	NM_006218.2	ccsbBroad304_14759

PIK3CA_WT.2	NM_006218.2	BRDN0000464880
PIK3CB_WT.1	BC114432.1	ccsbBroad304_01202
PIK3CB_WT.2	NM_006219.1	ccsbBroad304_14760
PIK3CD_WT	BC132919.1	ccsbBroad304_01204
PIK3R1_WT.1	BC030815.1	ccsbBroad304_14763
PIK3R1_WT.2	BC030815.1	ccsbBroad304_06728
PIK3R2_WT	BC014170.2	ccsbBroad304_11031
PKIA_WT	NM_006823.3	BRDN0000464858
PPARGC1A_WT.1	NM_013261.3	BRDN0000464872
PPARGC1A_WT.2	NM_013261.3	BRDN0000464873
PPP2R5C_WT.1	BC016183.1	ccsbBroad304_11052
PPP2R5C_WT.2	NM_002719.3	BRDN0000464928
PRKAA1_WT.1	BC037303.2	ccsbBroad304_14776
PRKAA1_WT.2	BC048980.1	ccsbBroad304_06770
PRKACA_WT.1	NM_002730.3	ccsbBroad304_14780
PRKACA_WT.2	NM_002730.3	BRDN0000464879
PRKACB_WT.1	BC016285.1	ccsbBroad304_01278
PRKACB_WT.2	BC035058.1	ccsbBroad304_14781
PRKACB_WT.3	BC035058.1	ccsbBroad304_06771
PRKACG_WT.1	BC039888.1	ccsbBroad304_06772
PRKACG_WT.2	BC039888.1	ccsbBroad304_14782
PRKACG_WT.3	NM_002732.3	BRDN0000464859
PRKCA_del1-325	NM_002737.2:c.4_975del	BRDN0000464962
PRKCA_K368R	NM_002737.2:c.1103A>G	BRDN0000464977
PRKCA_WT.1	BC109273.1	ccsbBroad304_01281
PRKCA_WT.2	NM_002737.2	ccsbBroad304_14788
PRKCE_del1-394	NM_005400.2:c.4_1182del	BRDN0000464963
PRKCE_K437R.mismatch	NM_005400.2:c.1310A>G	BRDN0000464984
PRKCE_WT.1	BC109033.2	ccsbBroad304_01283
PRKCE_WT.2	NM_005400.2	ccsbBroad304_14790
PRKCZ_del1-238	NM_002744.4:c.1_711del	BRDN0000464961
PRKCZ_K281R	NM_002744.4:c.842A>G	BRDN0000464964
PRKCZ_WT.1	BC008058.2	ccsbBroad304_01284
PRKCZ_WT.2	BC008058.2	ccsbBroad304_14796

PSENEN_WT	BC009575.1	ccsbBroad304_03668
PTEN_WT	NM_000314.4	BRDN0000464939
RAC1_Q61L	NM_006908.4:c.182A>T	BRDN0000464982
RAC1_T17N	NM_006908.4:c.50C>A	BRDN0000464983
RAC1_WT.1	NM_006908.4	BRDN0000464912
RAC1_WT.2	BC004247.1	ccsbBroad304_06831
RAF1_L613V	NM_002880.3:c.1837C>G	BRDN0000464981
RAF1_WT.1	BC018119.2	ccsbBroad304_14825
RAF1_WT.2	BC018119.2	ccsbBroad304_06837
RB1_WT.1.mismatch	NM_000321.2	BRDN0000464905
RB1_WT.2	BC039060.1	ccsbBroad304_06846
RBPJ_WT.1	BC020780.1	ccsbBroad304_06435
RBPJ_WT.2	NM_203283.2	BRDN0000464865
REL_WT.1	NM_002908.2	BRDN0000464942
REL_WT.2	NM_002908.2	BRDN0000464895
RELA_WT.1	BC014095.2	ccsbBroad304_11095
RELA_WT.2	NM_021975.3	BRDN0000464890
RELB_WT	BC028013.1	ccsbBroad304_01388
RHEB_WT.1	BC016155.1	ccsbBroad304_01398
RHEB_WT.2	BC066307.1	ccsbBroad304_06864
RHOA_Q63L	NM_001664.2:c.188A>T	BRDN0000464985
RHOA_T19N	NM_001664.2:c.56CA>AC	BRDN0000464975
RHOA_WT	BC001360.2	ccsbBroad304_00100
RICTOR_WT	BC029608.1	ccsbBroad304_13449
RIPK1_WT	BC126254.1	ccsbBroad304_14910
RPS6KB1_T389E.mismatch	RPS6KB1_c.1234AC>GA	BRDN0000464960
RPS6KB1_WT.1	NM_003161.3	BRDN0000464914
RPS6KB1_WT.2	BC053365.1	ccsbBroad304_14833
RPTOR_WT	BC064515.1	ccsbBroad304_12357
SDHA_WT	BC001380.2	ccsbBroad304_06926
SGK3_WT.1	BC015326.1	ccsbBroad304_02831
SGK3_WT.2	BC015326.1	ccsbBroad304_15022
SLIRP_WT.1	BC017895.1	ccsbBroad304_09091
SLIRP_WT.2	NM_031210.5	BRDN0000464957

SMAD3_WT.1	BC050743.1	ccsbBroad304_06549
SMAD3_WT.2	NM_001145102.1	BRDN0000464889
SMAD4_WT	BC002379.2	ccsbBroad304_00962
SMAD5_WT	BC009682.2	ccsbBroad304_00963
SMAD7_WT	BC074818.2	ccsbBroad304_00964
SMO_WT.1	NM_005631.4	BRDN0000464867
SMO_WT.2	NM_005631.4	BRDN0000464868
SMURF2_WT	BC093876.1	ccsbBroad304_03961
SOCS3_WT	BC060858.1	ccsbBroad304_02064
SRC_Y527F	NM_198291.1:c.1589A>T	BRDN0000464988
SREBF1_WT	BC057388.1	ccsbBroad304_06995
STAT1_WT	BC002704.2	ccsbBroad304_01607
STAT1_Y701F	NM_139266.2:c.2102A>T	BRDN0000464965
STAT3_C-C	NM_139276.2:c.1984GCTACCAAT>TGTACCTGT	BRDN0000464969
STAT3_WT	BC014482.1	ccsbBroad304_01609
STAT3_Y705F	NM_139276.2:c.2114A>T	BRDN0000464968
STK11_WT.1	BC007981.2	ccsbBroad304_01613
STK11_WT.2	BC007981.2	ccsbBroad304_14853
STK3_WT.1	BC010640.2	ccsbBroad304_14850
STK3_WT.2	BC010640.2	ccsbBroad304_07012
TBK1_WT.1	BC034950.2	ccsbBroad304_08116
TBK1_WT.2	BC034950.2	ccsbBroad304_15050
TCF4_WT.1	NM_003199.2	BRDN0000464887
TCF4_WT.2	BC125084.1	ccsbBroad304_07036
TGFB1_WT	BC000125.1	ccsbBroad304_07056
TGFBR1_K232R	NM_001130916.1	BRDN0000464986
TGFBR1_WT.1	BC071181.1	ccsbBroad304_01666
TGFBR1_WT.2	NM_004612.2	ccsbBroad304_14861
TGFBR1_WT.3	NM_001130916.1	BRDN0000464886
TGFBR2_WT.1	BC040499.1	ccsbBroad304_11186
TGFBR2_WT.2	BC040499.1	ccsbBroad304_14862
TNFAIP3_WT	BC114480.1	ccsbBroad304_01686
TP53_WT.1	NM_001126112.2	BRDN0000464908

TP53_WT.2	BC003596.1	ccsbBroad304_07088
TRAF2_WT	BC032410.1	ccsbBroad304_01710
TRAF3_WT.1	NM_145725.2	BRDN0000464940
TRAF3_WT.2	BC075086.2	ccsbBroad304_07098
TRAF5_WT	NM_145759.2	BRDN0000464918
TRAF6_WT.1	NM_145803.2	BRDN0000464951
TRAF6_WT.2	NM_145803.2	BRDN0000464952
TSC1_WT.1	NM_000368.4	BRDN0000464949
TSC1_WT.2	NM_000368.4	BRDN0000464950
TSC2_WT	BC150300.1	ccsbBroad304_07101
VEGFC_WT.1	BC035212.1	ccsbBroad304_07127
VEGFC_WT.2	NM_005429.3	BRDN0000464883
VHL_WT.1	BC058831.1	ccsbBroad304_07128
VHL_WT.2	NM_000551.3	BRDN0000464902
WNT5A_WT	BC064694.1	ccsbBroad304_01779
WWTR1_WT	BC014052.2	ccsbBroad304_02889
XBP1_S	NM_005080.3	BRDN0000464974
XBP1_WT.1	NM_005080.3	BRDN0000464900
XBP1_WT.2	NM_005080.3	BRDN0000464877
XBP1_WT.3	NM_005080.3	BRDN0000464878
XIAP_WT.1	NM_001204401.1	BRDN0000464906
XIAP_WT.2	BC032729.1	ccsbBroad304_05835
YAP1_WT.1	BC038235.1	ccsbBroad304_07601
YAP1_WT.2	NM_001130145.2	BRDN0000464852
YAP1_WT.3	NM_001195045.1	BRDN0000464856
YAP1_WT.4	NM_001195045.1	BRDN0000464857
