# Supplementary material for: Systematic morphological profiling of human gene and allele function via Cell Painting
Source: eLife. 2017 Mar 18;6:e24060. doi: 10.7554/eLife.24060 (PMC5386591; doi:10.7554/eLife.24060)
Supplement: Supplementary file 1. — (A) List of all the 323 constructs used in the experiment along with the target transcript and their public clone ID. (B) Replicate correlation is higher in the constitutively active mutant allele compared to the wild-type allele, except for AKT3_E17K. Constitutively active mutant annotations were obtained by literature search for all the mutants in the experiment showing a detectable phenotype. Genes shown here are only those where either the wild-type gene or its constitutively activating allele yielded a phenotype distinct from controls. (C) Pathways sorted based on proportion of their associated gene showing a detectable phenotype. (D) Highly correlated proteins (according to morphology in the Cell Painting assay) that have also been reported to interact physically. (E) Highly correlated genes (according to morphology in the Cell Painting assay) that have also been annotated to be related to the same pathway. (F) Gene Ontology terms associated with each gene cluster (Alexa and Rahnenführer, 2009). (G) Rank ordered list of distinctive features based on their z-scores for Cluster 19. (H): All genes/alleles in Cluster 8 and 10 induce cell rounding. (I) The NF-κB signaling pathway is the most enriched when searching for gene overexpressions that downregulate known YAP/TAZ targets (CYR61, CTGF, and BIRC5). DOI: http://dx.doi.org/10.7554/eLife.24060.016 [file elife-24060-supp1.zip › Supp_Files/1B - Constitutively active mutant vs WT replicate correlation.pdf]

B: Replicate correlation is higher in the constitutively active mutant allele compared to the wild-type allele, except for AKT3_E17K. Constitutively active mutant annotations were obtained by literature search for all the mutants in the experiment showing a detectable phenotype. Genes shown here are only those where either the wild-type gene or its constitutively activating allele yielded a phenotype distinct from controls.

Gene	Constitutively active mutation	Evidence on being constitutively active	Wild-type median replicate correlation	Mutant median replicate correlation
BRAF	V600E	(Cantwell-Dorris, O'Leary, and Sheils 2011)	0.57	0.79
RAF1	L613V	(Wu et al. 2011)	0.76	0.79
KRAS	G12V	(Eser et al. 2014)	0.43	0.72
CDC42	Q61L	(Nobes and Hall 1999)	0.64	0.89
AKT3	E17K	(M. S. Kim et al. 2008; M. A. Davies et al. 2008)	0.84	0.70
AKT1	E17K	(M. S. Kim et al. 2008; M. A. Davies et al. 2008)	0.40	0.93
RHOA	Q63L	(Z.-G. Zhang et al. 2006)	0.38	0.54
RAC1	Q61L	(Z.-G. Zhang et al. 2006)	0.30	0.53
