# Supplementary material for: Systematic morphological profiling of human gene and allele function via Cell Painting
Source: eLife. 2017 Mar 18;6:e24060. doi: 10.7554/eLife.24060 (PMC5386591; doi:10.7554/eLife.24060)
Supplement: Supplementary file 1. — (A) List of all the 323 constructs used in the experiment along with the target transcript and their public clone ID. (B) Replicate correlation is higher in the constitutively active mutant allele compared to the wild-type allele, except for AKT3_E17K. Constitutively active mutant annotations were obtained by literature search for all the mutants in the experiment showing a detectable phenotype. Genes shown here are only those where either the wild-type gene or its constitutively activating allele yielded a phenotype distinct from controls. (C) Pathways sorted based on proportion of their associated gene showing a detectable phenotype. (D) Highly correlated proteins (according to morphology in the Cell Painting assay) that have also been reported to interact physically. (E) Highly correlated genes (according to morphology in the Cell Painting assay) that have also been annotated to be related to the same pathway. (F) Gene Ontology terms associated with each gene cluster (Alexa and Rahnenführer, 2009). (G) Rank ordered list of distinctive features based on their z-scores for Cluster 19. (H): All genes/alleles in Cluster 8 and 10 induce cell rounding. (I) The NF-κB signaling pathway is the most enriched when searching for gene overexpressions that downregulate known YAP/TAZ targets (CYR61, CTGF, and BIRC5). DOI: http://dx.doi.org/10.7554/eLife.24060.016 [file elife-24060-supp1.zip › Supp_Files/1E - Highly correlated gene pairs belonging to the same pathway.pdf]

E: Highly correlated genes (according to morphology in the Cell Painting assay) that have also been annotated to be related to the same pathway. Two genes are highly correlated if their correlation is greater than 0.43 (see Methods).

Gene 1	Gene 2	Pathway annotation
CCND1_WT.2	CDK2_WT.2	Cell Cycle
ERN1_WT.1	HSPA5_WT	ER Stress/UPR
BRAF_WT.1	JUN_WT.1	MAPK
ELK1_WT	JUN_WT.1	MAPK
MAP2K3_WT	MAP2K4_WT.1	MAPK
MAP2K3_WT	MAP3K2_WT.1	MAPK
MAP3K2_WT.1	MAP3K7_WT	MAPK
MAP2K3_WT	MAP3K7_WT	MAPK
MAP3K2_WT.1	MAP3K9_WT	MAPK
MAP2K3_WT	MAP3K9_WT	MAPK
BRAF_WT.1	MAP3K9_WT	MAPK
ELK1_WT	MAP3K9_WT	MAPK
ARAF_WT.1	MAPK14_WT.1	MAPK
AKT1S1_WT.1	MLST8_WT	TOR
MAP3K9_WT	MOS_WT.1	MAPK
BRAF_WT.1	MOS_WT.1	MAPK
AKT3_WT.2	PIK3CB_WT.1	PI3K/AKT
FOXO1_WT.2	PIK3R1_WT.1	PI3K/AKT
FOXO1_WT.2	PTEN_WT	PI3K/AKT
PIK3R1_WT.1	PTEN_WT	PI3K/AKT
MAP3K9_WT	RAF1_WT.1	MAPK
BRAF_WT.1	RAF1_WT.1	MAPK
MOS_WT.1	RAF1_WT.1	MAPK
JUN_WT.1	RAF1_WT.1	MAPK
NFKB1_WT.1	RELB_WT	NFkB
CDC42_WT	RHOA_WT	Cytoskeletal Re-org
NFKB1_WT.1	TRAF2_WT	NFkB
HSP90B1_WT.2	XBP1_WT.1	ER Stress/UPR
WWTR1_WT	YAP1_WT.1	Hippo
