# Supplementary material for: Systematic morphological profiling of human gene and allele function via Cell Painting
Source: eLife. 2017 Mar 18;6:e24060. doi: 10.7554/eLife.24060 (PMC5386591; doi:10.7554/eLife.24060)
Supplement: Supplementary file 1. — (A) List of all the 323 constructs used in the experiment along with the target transcript and their public clone ID. (B) Replicate correlation is higher in the constitutively active mutant allele compared to the wild-type allele, except for AKT3_E17K. Constitutively active mutant annotations were obtained by literature search for all the mutants in the experiment showing a detectable phenotype. Genes shown here are only those where either the wild-type gene or its constitutively activating allele yielded a phenotype distinct from controls. (C) Pathways sorted based on proportion of their associated gene showing a detectable phenotype. (D) Highly correlated proteins (according to morphology in the Cell Painting assay) that have also been reported to interact physically. (E) Highly correlated genes (according to morphology in the Cell Painting assay) that have also been annotated to be related to the same pathway. (F) Gene Ontology terms associated with each gene cluster (Alexa and Rahnenführer, 2009). (G) Rank ordered list of distinctive features based on their z-scores for Cluster 19. (H): All genes/alleles in Cluster 8 and 10 induce cell rounding. (I) The NF-κB signaling pathway is the most enriched when searching for gene overexpressions that downregulate known YAP/TAZ targets (CYR61, CTGF, and BIRC5). DOI: http://dx.doi.org/10.7554/eLife.24060.016 [file elife-24060-supp1.zip › Supp_Files/1F - Gene Ontology terms associated with each gene cluster.pdf]

F: Gene Ontology terms associated with each gene cluster. For clusters containing at least two genes, associated GO terms (limited to those related to Biological Processes) are listed. Mutant and wild-type alleles of a gene are considered the same in the analysis. Clusters containing a single gene are omitted from the table. In order to make the analysis unbiased, the gene universe is set to the genes showing up in the clusters. The p-values are not adjusted and obtained by the Fisher's exact test, using topGO package in R (Alexa and Rahnenführer 2009).

Cluster ID	Gene/Alleles	GO ID	GO Term	# of genes associated with the GO term in the cluster vs. all the clusters	p-value
2	TBK1 HSP90B1 CDK4_R24C STAT3_C-C	GO:0001654	eye development	2/3	0.014
		GO:0007423	sensory organ development	2/7	0.086
		GO:0016032	viral process	2/7	0.086
		GO:0044403	symbiosis, encompassing mutualism through parasitism	2/7	0.086
		GO:0044419	interspecies interaction between organisms	2/7	0.086
3	CDKN1A KRAS_G12V HRAS_G12V MAP2K4 MAP2K3	GO:0071900	regulation of protein serine/threonine kinase activity	5/17	0.0026
		GO:0045860	positive regulation of protein kinase activity	5/20	0.0066

		GO:0051385	response to mineralocorticoid	2/2	0.0078
		GO:0043405	regulation of MAP kinase activity	4/12	0.0086
		GO:0043406	positive regulation of MAP kinase activity	4/12	0.0086
4	MAP3K2 CDC42_Q61L MAP3K9	GO ID	GO Term	# of genes associated with the GO term in the cluster vs. all the clusters	p-value
		GO:0032874	positive regulation of stress-activated MAPK cascade	3/9	0.004
		GO:0046330	positive regulation of JNK cascade	3/9	0.004
		GO:0070304	positive regulation of stress-activated protein kinase signaling cascade	3/9	0.004
		GO:0007254	JNK cascade	3/10	0.0058
		GO:0046328	regulation of JNK cascade	3/10	0.0058
5	XBP1 MAPK14 RBPJ	GO ID	GO Term	# of genes associated with the GO term in the cluster vs. all the clusters	p-value
		GO:0050663	cytokine secretion	3/4	0.00019
		GO:0051147	regulation of muscle cell differentiation	3/4	0.00019
		GO:0007517	muscle organ development	3/5	0.00048
		GO:0051146	striated muscle cell	3/5	0.00048

			differentiation		
		GO:0051153	regulation of striated muscle cell differentiation	3/3	4.80E-05
6	RAF1_L613V BRAF_V600E	GO ID	GO Term	# of genes associated with the GO term in the cluster vs. all the clusters	p-value
		GO:0033135	regulation of peptidyl-serine phosphorylation	2/5	0.0078
		GO:0033138	positive regulation of peptidyl-serine phosphorylation	2/5	0.0078
		GO:0023061	signal release	2/6	0.0118
		GO:0035019	somatic stem cell population maintenance	2/6	0.0118
		GO:0007411	axon guidance	2/8	0.022
7	RAC1_T17N AKT1_E17K AKT3 AKT3_E17K CDC42_T17N	GO ID	GO Term	# of genes associated with the GO term in the cluster vs. all the clusters	p-value
		GO:0031294	lymphocyte costimulation	3/4	0.00076
		GO:0031295	T cell costimulation	3/4	0.00076
		GO:0045834	positive regulation of lipid metabolic process	3/5	0.00186
		GO:0010256	endomembrane system organization	3/6	0.00366
		GO:0030307	positive regulation of cell growth	2/2	0.00471

8	RHOA_Q63L PRKACA GLI1	GO ID	GO Term	# of genes associated with the GO term in the cluster vs. all the clusters	p-value
		GO:0007043	cell-cell junction assembly	2/2	0.0024
		GO:0043297	apical junction assembly	2/2	0.0024
		GO:0001503	ossification	3/8	0.0027
		GO:0007224	smoothened signaling pathway	2/3	0.007
		GO:0007283	spermatogenesis	2/3	0.007
9	EIF4EBP1 DIABLO	NA			
10	ELK1 RHOA	NA			
11	CDC42 TRAF2	GO ID	GO Term	# of genes associated with the GO term in the cluster vs. all the clusters	p-value
		GO:0043393	regulation of protein binding	2/4	0.0047
		GO:0051098	regulation of binding	2/7	0.0165
		GO:0016567	protein ubiquitination	2/8	0.022
		GO:0022407	regulation of cell-cell adhesion	2/8	0.022
		GO:0022409	positive regulation of cell-cell adhesion	2/8	0.022
12	SMAD4 RPS6KB1 SMO	GO ID	GO Term	# of genes associated with the GO term in the cluster vs.	p-value

				all the clusters	
		GO:0001657	ureteric bud development	2/2	0.0024
		GO:0001658	branching involved in ureteric bud morphogenesis	2/2	0.0024
		GO:0001823	mesonephros development	2/2	0.0024
		GO:0009798	axis specification	2/2	0.0024
		GO:0009952	anterior/posterior pattern specification	2/2	0.0024
13	CDK2<br>AKT1S1	NA			
14	PIK3CB<br>TRAF5<br>MAP3K7	GO ID	GO Term	# of genes associated with the GO term in the cluster vs. all the clusters	p-value
		GO:0002250	adaptive immune response	2/4	0.014
		GO:0050851	antigen receptor-mediated signaling pathway	2/5	0.023
		GO:0050852	T cell receptor signaling pathway	2/5	0.023
		GO:0043122	regulation of I-kappaB kinase/NF-kappaB signaling	2/6	0.033
		GO:0043123	positive regulation of I-kappaB kinase/NF-kappaB signaling	2/6	0.033
15	JUN<br>STK3	GO ID	GO Term	# of genes associated with the GO term in the	p-value

				cluster vs. all the clusters	
		GO:0051098	regulation of binding	2/7	0.016
		GO:0002520	immune system development	2/10	0.035
		GO:0008285	negative regulation of cell proliferation	2/10	0.035
		GO:0030097	hemopoiesis	2/10	0.035
		GO:0048534	hematopoietic or lymphoid organ development	2/10	0.035
16	PRKCE GRB10	GO ID	GO Term	# of genes associated with the GO term in the cluster vs. all the clusters	p-value
		GO:0010675	regulation of cellular carbohydrate metabolic process	2/5	0.0078
		GO:0006109	regulation of carbohydrate metabolic process	2/6	0.0118
		GO:0044262	cellular carbohydrate metabolic process	2/6	0.0118
		GO:0005996	monosaccharide metabolic process	2/7	0.0165
		GO:0051051	negative regulation of transport	2/8	0.022
18	CEBPA JUN	GO ID	GO Term	# of genes associated with the GO term in the cluster vs. all the clusters	p-value

		GO:0001889	liver development	2/3	0.0024
		GO:0061008	hepaticobiliary system development	2/3	0.0024
		GO:0071248	cellular response to metal ion	2/3	0.0024
		GO:0002573	myeloid leukocyte differentiation	2/6	0.0118
		GO:0007005	mitochondrion organization	2/6	0.0118
19	KRAS<br>BRAF<br>RAF1<br>MOS	GO ID	GO Term	# of genes associated with the GO term in the cluster vs. all the clusters	p-value
		GO:0000186	activation of MAPKK activity	4/9	0.0005
		GO:0032147	activation of protein kinase activity	4/17	0.0095
		GO:0007411	axon guidance	3/8	0.0099
		GO:0097485	neuron projection guidance	3/8	0.0099
		GO:0007265	Ras protein signal transduction	3/9	0.0146
20	YAP1<br>WWTR1	GO ID	GO Term	# of genes associated with the GO term in the cluster vs. all the clusters	p-value
		GO:0035850	epithelial cell differentiation involved in kidney development	2/2	0.00078
		GO:0061005	cell differentiation involved	2/2	0.00078

			in kidney development		
		GO:0072160	nephron tubule epithelial cell differentiation	2/2	0.00078
		GO:0072170	metanephric tubule development	2/2	0.00078
		GO:0072182	regulation of nephron tubule epithelial cell differentiation	2/2	0.00078
21	PIK3R1<br>PTEN	GO ID	GO Term	# of genes associated with the GO term in the cluster vs. all the clusters	p-value
		GO:0001953	negative regulation of cell-matrix adhesion	2/2	0.00078
		GO:0007162	negative regulation of cell adhesion	2/2	0.00078
		GO:0010812	negative regulation of cell-substrate adhesion	2/2	0.00078
		GO:0006650	glycerophospholipid metabolic process	2/3	0.00235
		GO:0006661	phosphatidylinositol biosynthetic process	2/3	0.00235
23	NFKB1<br>PRKACG	GO ID	GO Term	# of genes associated with the GO term in the cluster vs. all the clusters	p-value
		GO:0044283	small molecule biosynthetic process	2/5	0.0078
		GO:0016051	carbohydrate biosynthetic process	2/6	0.0118

		GO:0002220	innate immune response activating cell surface receptor signaling pathway	2/7	0.0165
		GO:0002223	stimulatory C-type lectin receptor signaling pathway	2/7	0.0165
		GO:0005975	carbohydrate metabolic process	2/10	0.0353
24	SMURF2<br>DVL3	GO ID	GO Term	# of genes associated with the GO term in the cluster vs. all the clusters	p-value
		GO:0030177	positive regulation of Wnt signaling pathway	2/5	0.0078
		GO:0090263	positive regulation of canonical Wnt signaling pathway	2/5	0.0078
		GO:0060828	regulation of canonical Wnt signaling pathway	2/10	0.0353
		GO:0030111	regulation of Wnt signaling pathway	2/11	0.0431
		GO:0060070	canonical Wnt signaling pathway	2/12	0.0518
25	PRKCZ_del1-238<br>FOXO1	GO ID	GO Term	# of genes associated with the GO term in the cluster vs. all the clusters	p-value
		GO:0001933	negative regulation of protein phosphorylation	2/9	0.028
		GO:0031400	negative regulation of protein modification process	2/9	0.028

		GO:0010563	negative regulation of phosphorus metabolic process	2/11	0.043
		GO:0042326	negative regulation of phosphorylation	2/11	0.043
		GO:0045936	negative regulation of phosphate metabolic process	2/11	0.043
