# Supplementary material for: Systematic morphological profiling of human gene and allele function via Cell Painting
Source: eLife. 2017 Mar 18;6:e24060. doi: 10.7554/eLife.24060 (PMC5386591; doi:10.7554/eLife.24060)
Supplement: Supplementary file 1. — (A) List of all the 323 constructs used in the experiment along with the target transcript and their public clone ID. (B) Replicate correlation is higher in the constitutively active mutant allele compared to the wild-type allele, except for AKT3_E17K. Constitutively active mutant annotations were obtained by literature search for all the mutants in the experiment showing a detectable phenotype. Genes shown here are only those where either the wild-type gene or its constitutively activating allele yielded a phenotype distinct from controls. (C) Pathways sorted based on proportion of their associated gene showing a detectable phenotype. (D) Highly correlated proteins (according to morphology in the Cell Painting assay) that have also been reported to interact physically. (E) Highly correlated genes (according to morphology in the Cell Painting assay) that have also been annotated to be related to the same pathway. (F) Gene Ontology terms associated with each gene cluster (Alexa and Rahnenführer, 2009). (G) Rank ordered list of distinctive features based on their z-scores for Cluster 19. (H): All genes/alleles in Cluster 8 and 10 induce cell rounding. (I) The NF-κB signaling pathway is the most enriched when searching for gene overexpressions that downregulate known YAP/TAZ targets (CYR61, CTGF, and BIRC5). DOI: http://dx.doi.org/10.7554/eLife.24060.016 [file elife-24060-supp1.zip › Supp_Files/1H - All genes and alleles in Cluster 8 and 10 induce cell rounding.pdf]

H: All genes/alleles in Cluster 8 and 10 induce cell rounding. “Form Factor” is a measurement in the morphological profile that is defined as the area of an object divided by its perimeter; the metric shown here was calculated on the shape of the entire cell, which is defined by RNA staining (SYTO14). The positive z-scores seen here indicate increased cell rounding.

Treatment	Cells_AreaShape_FormFactor z-score
RHOA_Q63L	6.39
PRKACA_WT.1	4.18
PRKACA_WT.2	6.64
GLI1_WT	3.46
ELK1_WT	4.98
RHOA_WT	3.42
