# Supplementary material for: Systematic morphological profiling of human gene and allele function via Cell Painting
Source: eLife. 2017 Mar 18;6:e24060. doi: 10.7554/eLife.24060 (PMC5386591; doi:10.7554/eLife.24060)
Supplement: Supplementary file 1. — (A) List of all the 323 constructs used in the experiment along with the target transcript and their public clone ID. (B) Replicate correlation is higher in the constitutively active mutant allele compared to the wild-type allele, except for AKT3_E17K. Constitutively active mutant annotations were obtained by literature search for all the mutants in the experiment showing a detectable phenotype. Genes shown here are only those where either the wild-type gene or its constitutively activating allele yielded a phenotype distinct from controls. (C) Pathways sorted based on proportion of their associated gene showing a detectable phenotype. (D) Highly correlated proteins (according to morphology in the Cell Painting assay) that have also been reported to interact physically. (E) Highly correlated genes (according to morphology in the Cell Painting assay) that have also been annotated to be related to the same pathway. (F) Gene Ontology terms associated with each gene cluster (Alexa and Rahnenführer, 2009). (G) Rank ordered list of distinctive features based on their z-scores for Cluster 19. (H): All genes/alleles in Cluster 8 and 10 induce cell rounding. (I) The NF-κB signaling pathway is the most enriched when searching for gene overexpressions that downregulate known YAP/TAZ targets (CYR61, CTGF, and BIRC5). DOI: http://dx.doi.org/10.7554/eLife.24060.016 [file elife-24060-supp1.zip › Supp_Files/1I - The NF-kB signaling pathway is the most enriched when searching for gene overexpressions that downregulate known YAP and TAZ targets.pdf]

I: The NF-κB signaling pathway is the most enriched when searching for gene overexpressions that downregulate known YAP/TAZ targets (CYR61, CTGF, and BIRC5). The ORFs corresponding to each pathway are listed in the last column.

Pathway Name	Benjamini and Hochberg (BH) Adjusted p-value	Gene IDs
NF-kappa B signaling pathway	2e-08	BCL10/GADD45B/CD40/LTBR/TIRAP/RELB/TRAF2/LYN
Epstein-Barr virus infection	5.3e-06	CDKN1B/IFNG/CDKN1A/CD40/RELB/MAP2K6/TRAF2/LYN
Cell cycle	0.0011	CDKN2C/CDKN1B/CDKN1A/GADD45B/GADD45A
Osteoclast differentiation	0.0011	IFNB1/IFNG/RELB/MAP2K6/TRAF2
FoxO signaling pathway	0.0011	CDKN1B/CDKN1A/GADD45B/RAF1/GADD45A
Hepatitis B	0.0013	CDKN1B/IFNB1/CDKN1A/TIRAP/RAF1
MAPK signaling pathway	0.0013	GADD45B/RELB/RAF1/MAP2K6/TRAF2/GADD45A
HTLV-I infection	0.0013	CDKN2C/CDKN1A/CD40/LTBR/RELB/IL2RB
Jak-STAT signaling pathway	0.0013	IFNB1/IFNG/CDKN1A/RAF1/IL2RB
Tuberculosis	0.002	IFNB1/IFNG/BCL10/TIRAP/RAF1
Transcriptional misregulation in cancer	0.002	CDKN2C/CDKN1B/CDKN1A/CD40/IL2RB
Endocrine resistance	0.002	CDKN2C/CDKN1B/CDKN1A/RAF1
HIF-1 signaling pathway	0.0023	CDKN1B/IFNG/CDKN1A/LTBR
Toll-like receptor signaling pathway	0.0023	IFNB1/CD40/TIRAP/MAP2K6
Viral carcinogenesis	0.0026	CDKN1B/CDKN1A/LTBR/TRAF2/LYN
Hepatitis C	0.0049	IFNB1/CDKN1A/RAF1/TRAF2
Measles	0.005	CDKN1B/IFNB1/IFNG/IL2RB
Apoptosis	0.0053	GADD45B/RAF1/TRAF2/GADD45A
Cytokine-cytokine receptor interaction	0.0067	IFNB1/IFNG/CD40/LTBR/IL2RB
p53 signaling pathway	0.0069	CDKN1A/GADD45B/GADD45A
Fc epsilon RI signaling pathway	0.0069	RAF1/MAP2K6/LYN
B cell receptor	0.0072	BCL10/RAF1/LYN

signaling pathway		
Chronic myeloid leukemia	0.0072	CDKN1B/CDKN1A/RAF1
Influenza A	0.009	IFNB1/IFNG/RAF1/MAP2K6
ErbB signaling pathway	0.011	CDKN1B/CDKN1A/RAF1
Prostate cancer	0.011	CDKN1B/CDKN1A/RAF1
PI3K-Akt signaling pathway	0.014	CDKN1B/IFNB1/CDKN1A/RAF1/IL2RB
T cell receptor signaling pathway	0.017	IFNG/BCL10/RAF1
Toxoplasmosis	0.022	IFNG/CD40/MAP2K6
Allograft rejection	0.024	IFNG/CD40
Bladder cancer	0.027	CDKN1A/RAF1
Natural killer cell mediated cytotoxicity	0.029	IFNB1/IFNG/RAF1
Breast cancer	0.033	CDKN1A/RAF1/HEY1
Intestinal immune network for IgA production	0.033	CD40/LTBR
Malaria	0.033	IFNG/CD40
Long-term depression	0.048	RAF1/LYN
