# Supplementary material for: Systematic morphological profiling of human gene and allele function via Cell Painting
Source: eLife. 2017 Mar 18;6:e24060. doi: 10.7554/eLife.24060 (PMC5386591; doi:10.7554/eLife.24060)

Cluster 11

a

b

c

How similar is this cluster to the other clusters?

1

Genes in the cluster along with the pathways as annotated by experts

Treatment	Expert Annotation		
	Pathway	Regulation Type	Cell Count z-score
CDC42_WT	Canonical Cytoskeletal Re-org	Activator	-0.26
TRAF2_WT	Canonical NFkB	Activator	-0.29

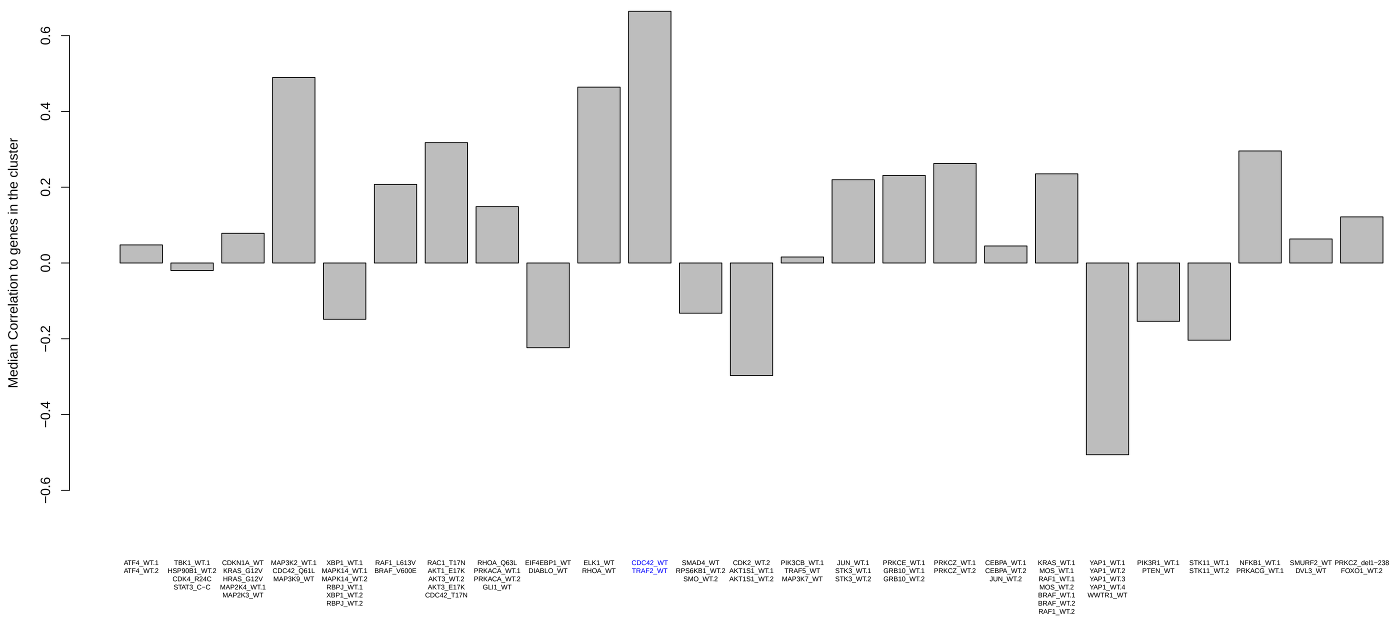

Top 5 genes/alleles negatively correlated to the cluster

Treatment	Expert Annotation			
	Pathway	Regulation Type	Mean Correlation	Standard Deviation
YAP1_WT.4	Canonical Hippo	Inhibitor	-0.55	0.00
YAP1_WT.1	Canonical Hippo	Inhibitor	-0.53	0.04
YAP1_WT.3	Canonical Hippo	Inhibitor	-0.50	0.02
WWTR1_WT	Canonical Hippo	Inhibitor	-0.49	0.09
YAP1_WT.2	Canonical Hippo	Inhibitor	-0.45	0.02

What groups of morphological features are distinguishing in the cluster relative to the untreated samples? (maximum of absolute m-score for the features belonging to the same category; m-score defined as median of a feature z-score across genes in the cluster) Black means no feature is available in the category

Which individual morphological features are distinguishing in the cluster relative to the untreated samples? Blue/Red means the feature has a positive/negative z-score. Size is proportional to the z-score value.

How strongly are genes within the cluster correlated?

2

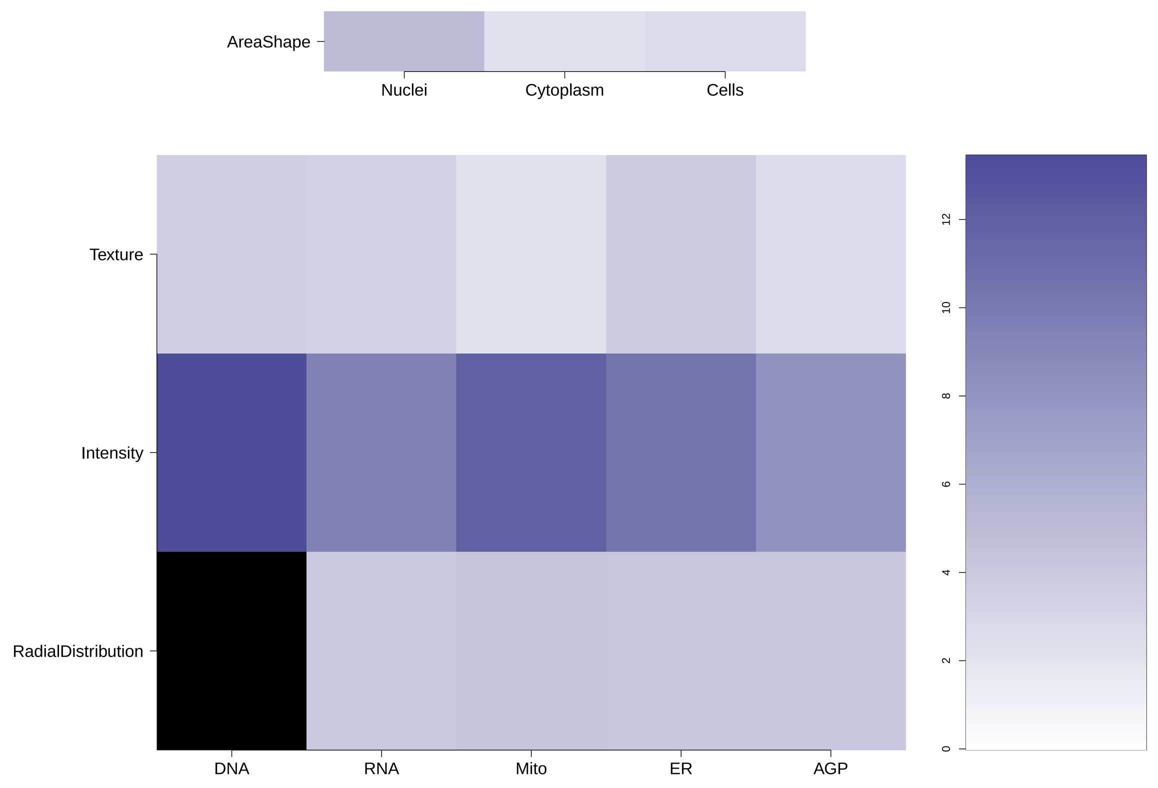

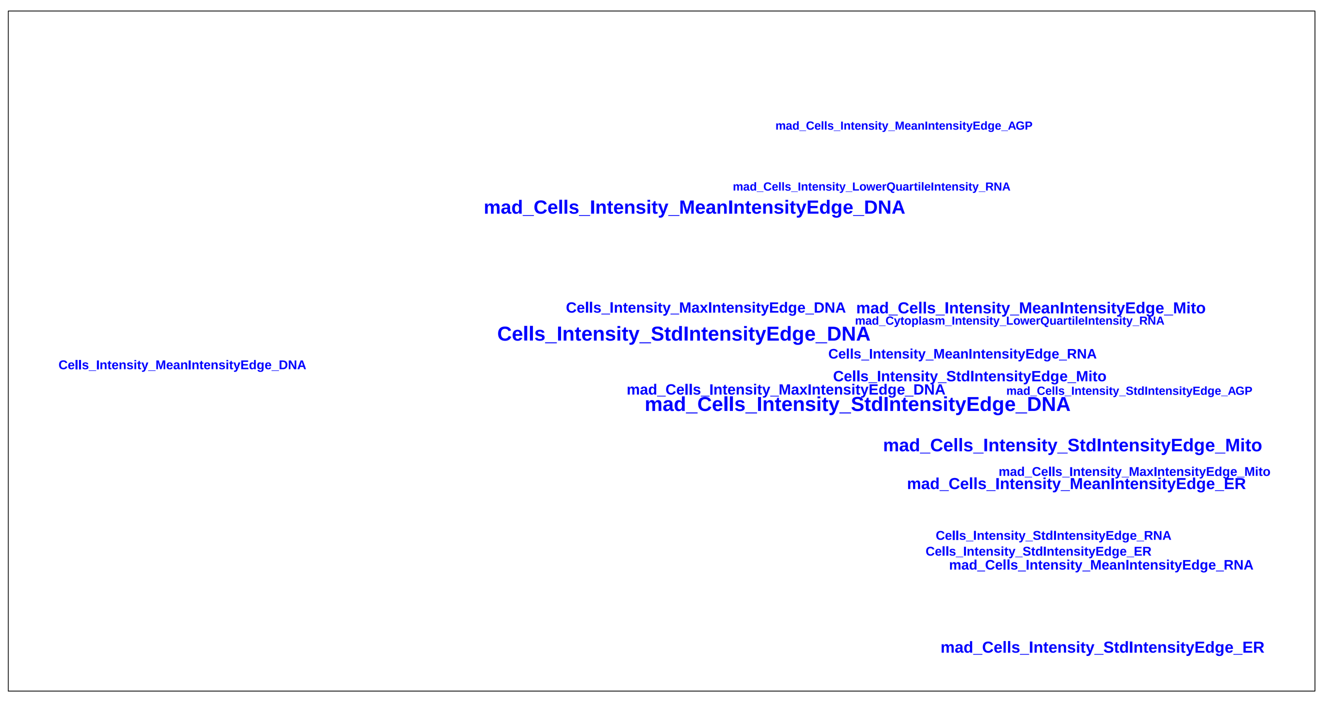

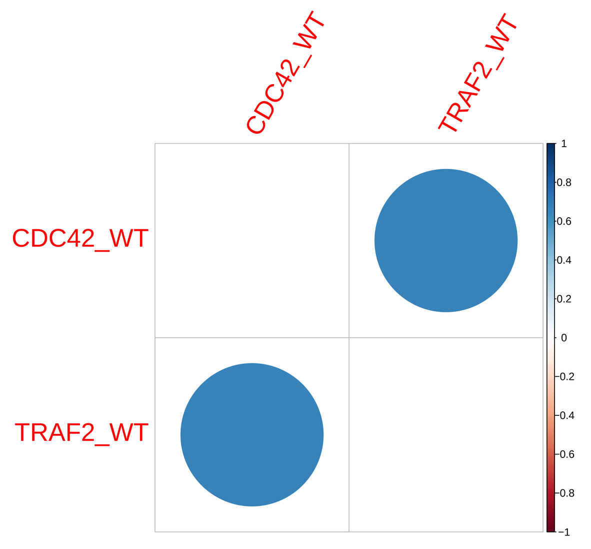

Images show 11% area of a well. Scale bar is 65.60 μm. Plate : 41744 - Genes in the Cluster (Channels are sorted based on their dominance in the grid plot)

Empty CDC42_WT TRAF2_WT

3

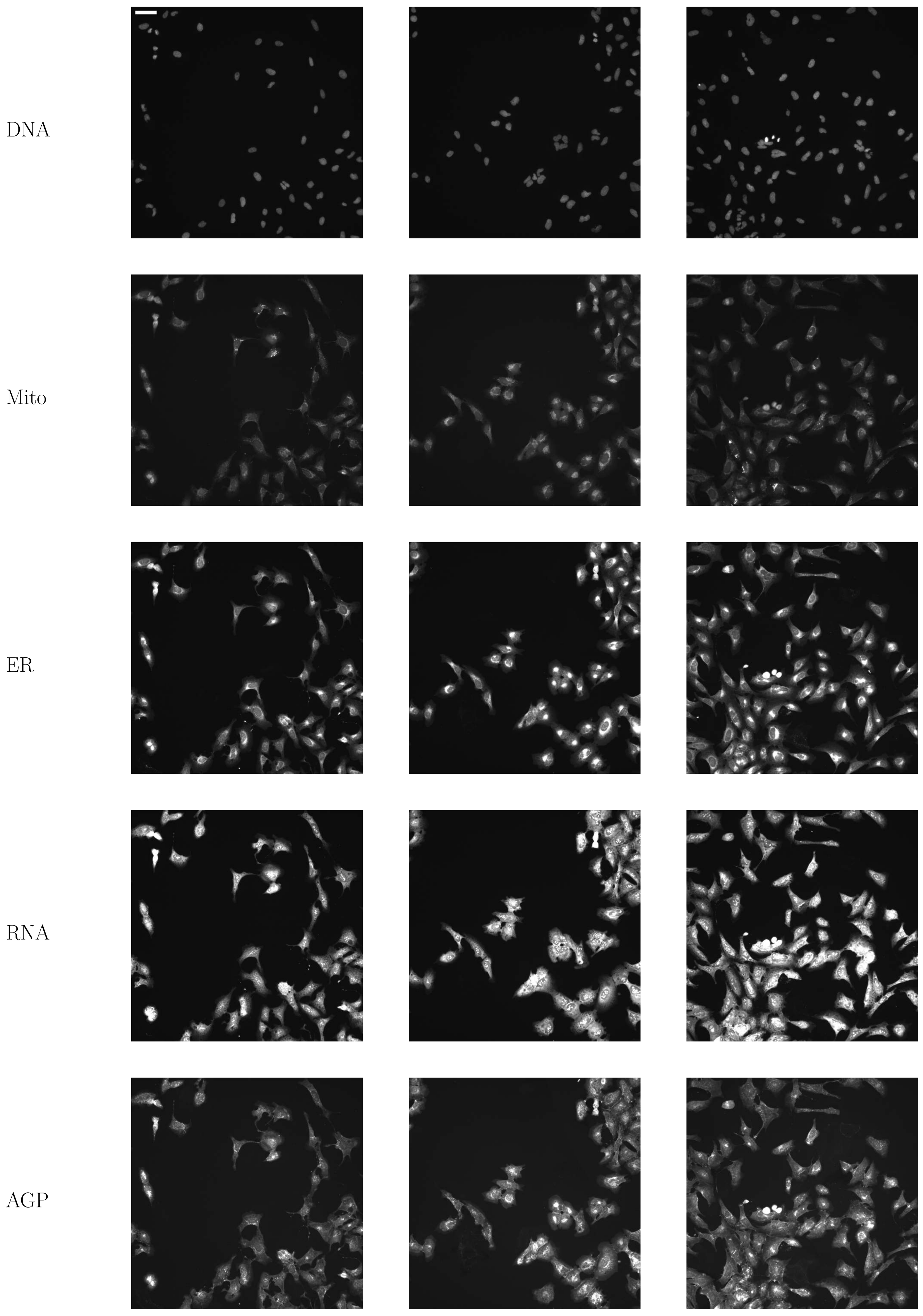

Supplement: Supplementary file 2. — The details of the contents have been described in Figure 5. DOI: http://dx.doi.org/10.7554/eLife.24060.017 [file elife-24060-supp2.zip › Supplementary file 2/type A/11A.pdf]
