# Supplementary material for: Systematic morphological profiling of human gene and allele function via Cell Painting
Source: eLife. 2017 Mar 18;6:e24060. doi: 10.7554/eLife.24060 (PMC5386591; doi:10.7554/eLife.24060)

Cluster 13

a b c

How similar is this cluster to the other clusters?

1

Genes in the cluster along with the pathways as annotated by experts

	Expert Annotation		
Treatment	Pathway	Regulation Type	Cell Count z-score
CDK2_WT.2	Canonical Cell Cycle	Activator	0.82
AKT1S1_WT.1	TOR	Inhibitor	-0.25
AKT1S1_WT.2	TOR	Inhibitor	-0.92

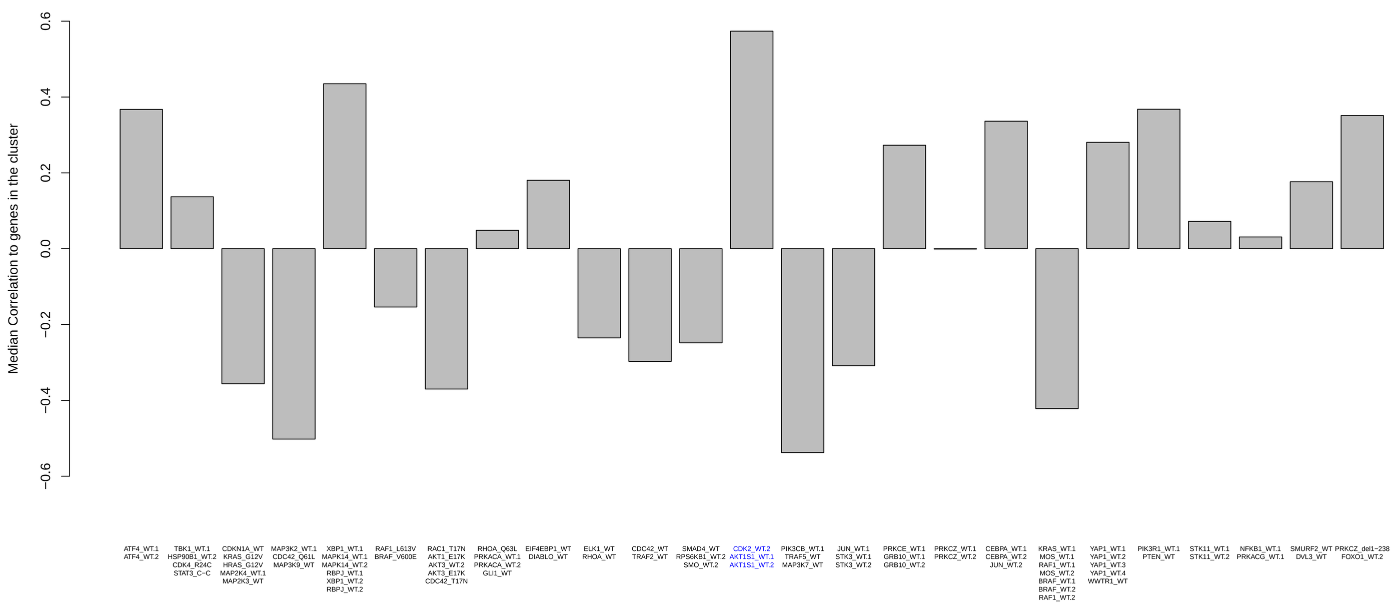

Top 5 genes/alleles negatively correlated to the cluster

	Expert Annotation			
Treatment	Pathway	Regulation Type	Mean Correlation	Standard Deviation
MAP3K2_WT.1	Canonical MAPK	Activator	-0.57	0.05
TRAF5_WT	Canonical NFkB	Activator	-0.54	0.07
MAP2K3_WT	Canonical MAPK	Activator	-0.53	0.07
MOS_WT.2	MAPK	Activator	-0.49	0.07
PIK3CB_WT.1	Canonical PI3K/AKT	Activator	-0.49	0.12

2

What groups of morphological features are distinguishing in the cluster relative to the untreated samples? (maximum of absolute m-score for the features belonging to the same category; m-score defined as median of a feature z-score across genes in the cluster) Black means no feature is available in the category

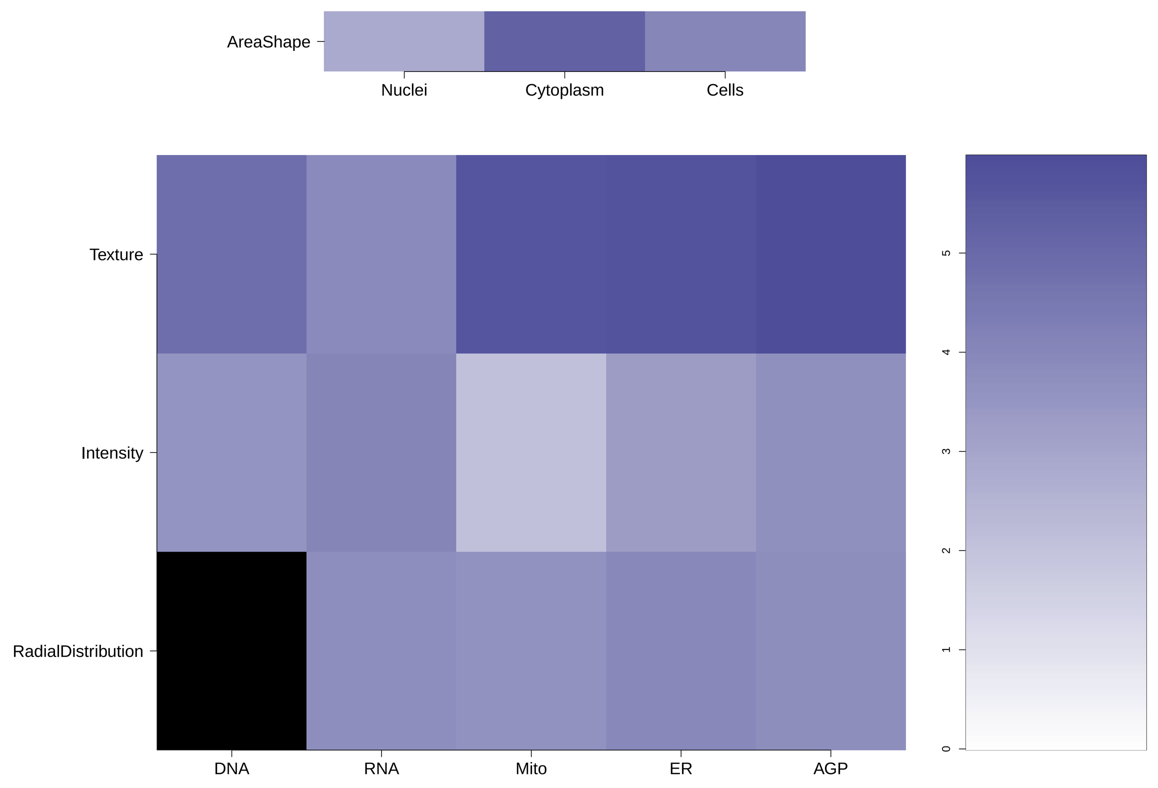

Which individual morphological features are distinguishing in the cluster relative to the untreated samples? Blue/Red means the feature has a positive/negative z-score. Size is proportional to the z-score value.

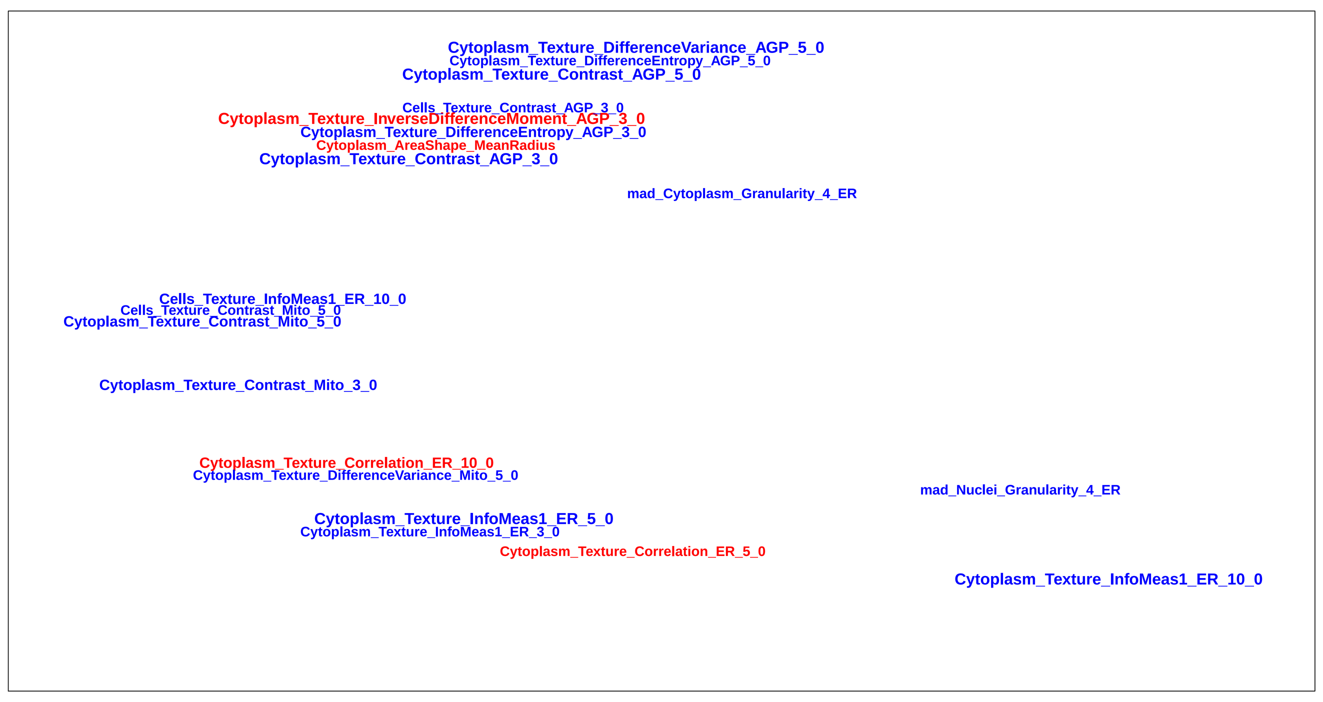

How strongly are genes within the cluster correlated?

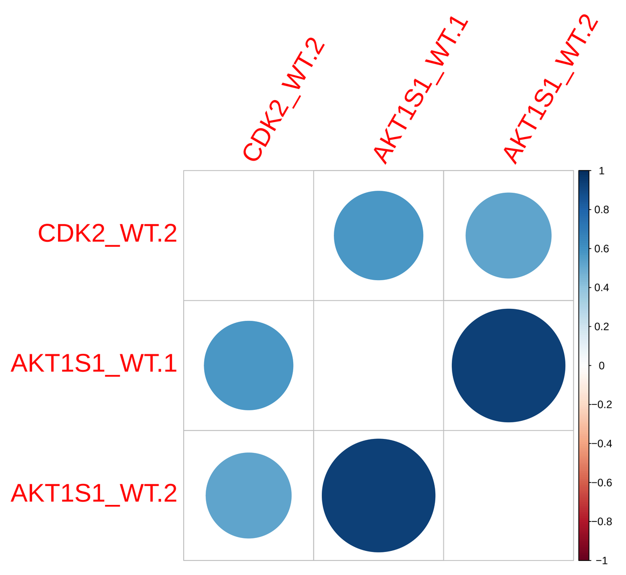

3

Images show 11% area of a well. Scale bar is 65.60 μm. Plate : 41744 - Genes in the Cluster (Channels are sorted based on their dominance in the grid plot)

Empty CDK2_WT.2 AKT1S1_WT.1 AKT1S1_WT.2

AGP

ER

Mito

RNA

DNA

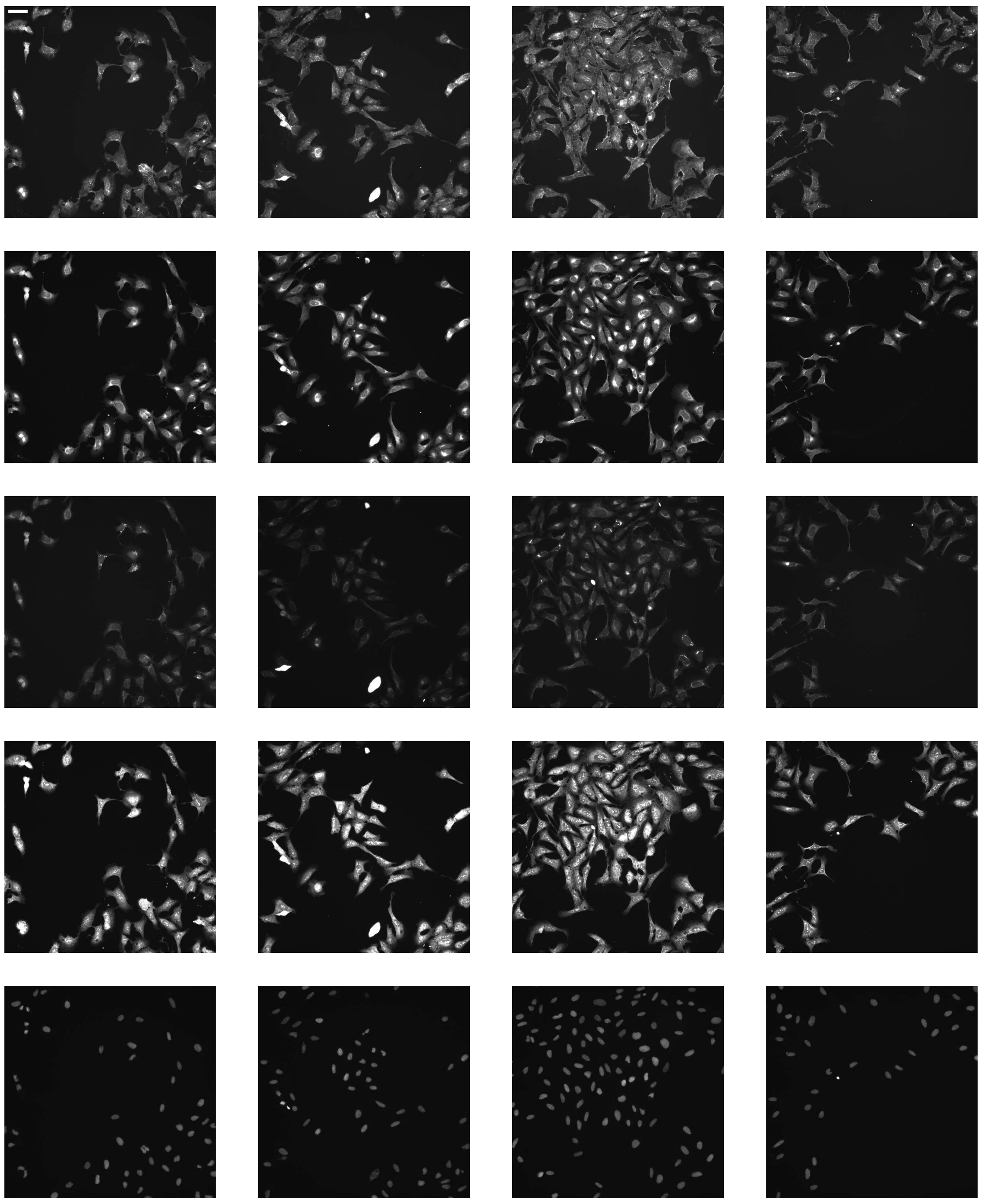

Supplement: Supplementary file 2. — The details of the contents have been described in Figure 5. DOI: http://dx.doi.org/10.7554/eLife.24060.017 [file elife-24060-supp2.zip › Supplementary file 2/type A/13A.pdf]
