# Supplementary material for: Systematic morphological profiling of human gene and allele function via Cell Painting
Source: eLife. 2017 Mar 18;6:e24060. doi: 10.7554/eLife.24060 (PMC5386591; doi:10.7554/eLife.24060)

Cluster 15

a

b

c

How similar is this cluster to the other clusters?

1

Genes in the cluster along with the pathways as annotated by experts

	Expert Annotation		
Treatment	Pathway	Regulation Type	Cell Count z-score
STK3_WT.1	Canonical Hippo	Activator	-0.69
STK3_WT.2	Canonical Hippo	Activator	-1.00
JUN_WT.1	Canonical MAPK	Activator	0.44

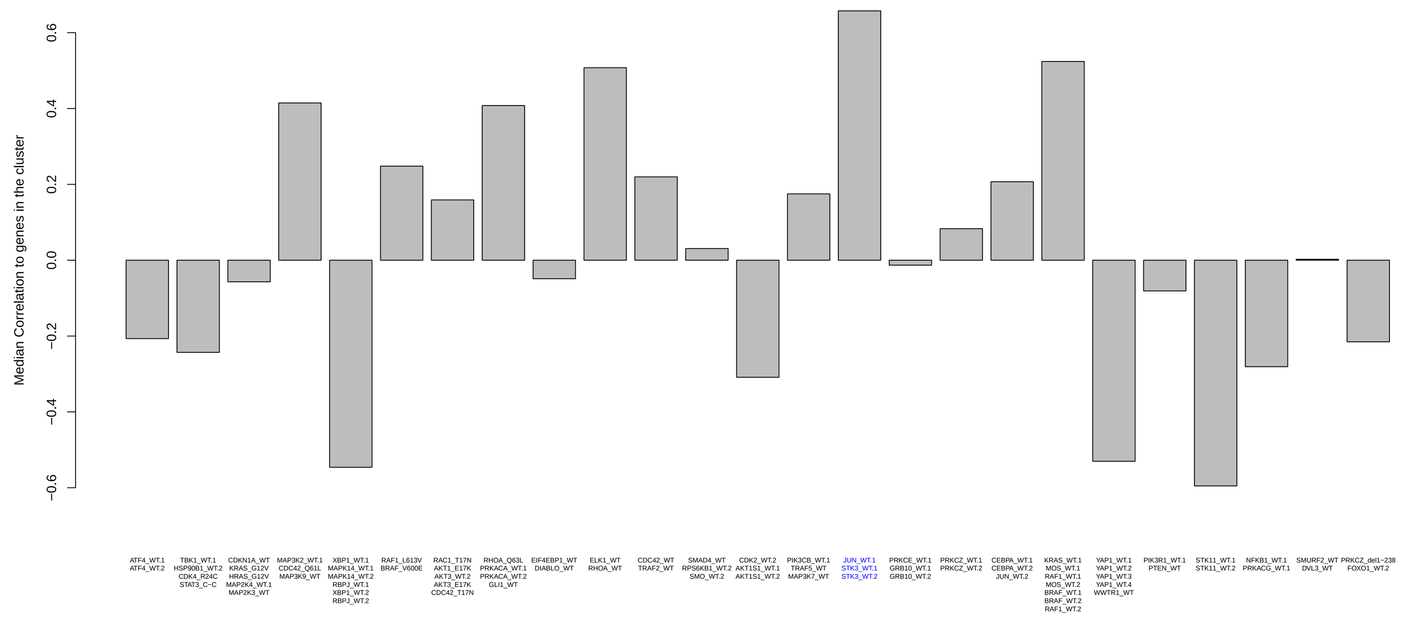

Top 5 genes/alleles negatively correlated to the cluster

	Expert Annotation			
Treatment	Pathway	Regulation Type	Mean Correlation	Standard Deviation
STK11_WT.2	Canonical TOR	Inhibitor	-0.68	0.03
RBPJ_WT.2	NOTCH	Activator	-0.61	0.09
YAP1_WT.2	Canonical Hippo	Inhibitor	-0.59	0.07
YAP1_WT.3	Canonical Hippo	Inhibitor	-0.57	0.06
MAPK14_WT.2	Canonical MAPK	Activator	-0.56	0.07

2

What groups of morphological features are distinguishing in the cluster relative to the untreated samples? (maximum of absolute m-score for the features belonging to the same category; m-score defined as median of a feature z-score across genes in the cluster) Black means no feature is available in the category

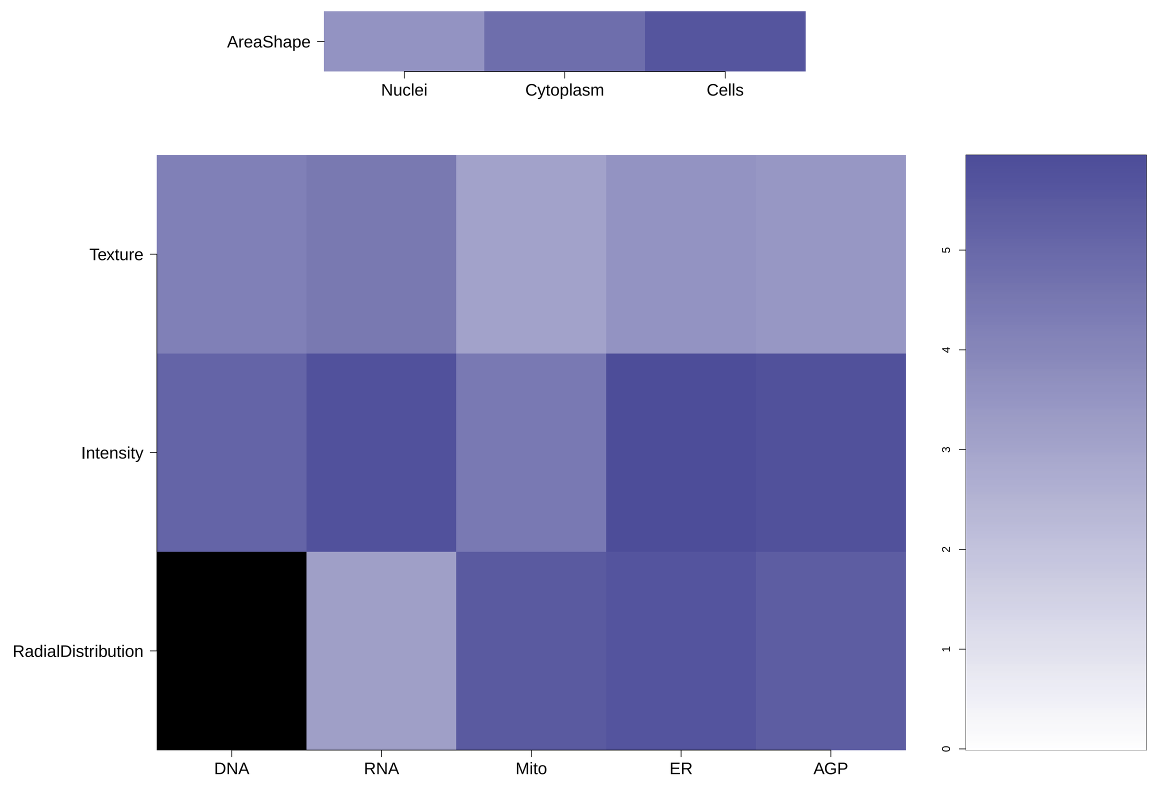

Which individual morphological features are distinguishing in the cluster relative to the untreated samples? Blue/Red means the feature has a positive/negative z-score. Size is proportional to the z-score value.

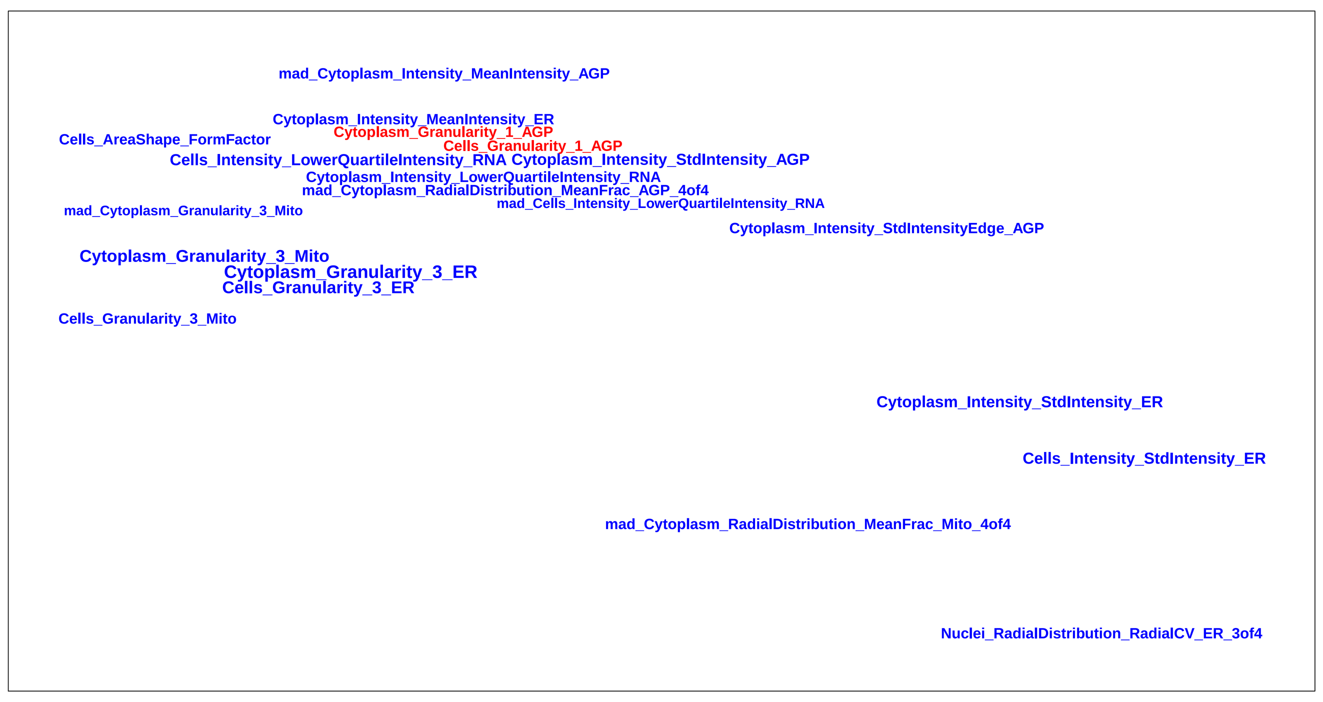

How strongly are genes within the cluster correlated?

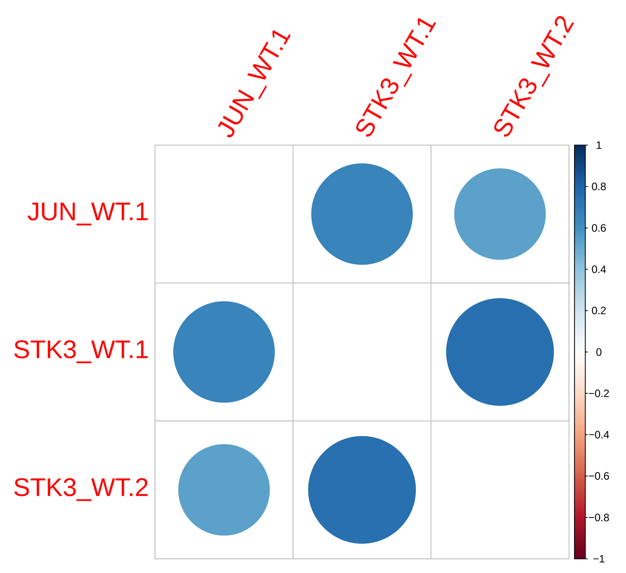

3

Images show 11% area of a well. Scale bar is 65.60 μm. Plate : 41744 - Genes in the Cluster (Channels are sorted based on their dominance in the grid plot)

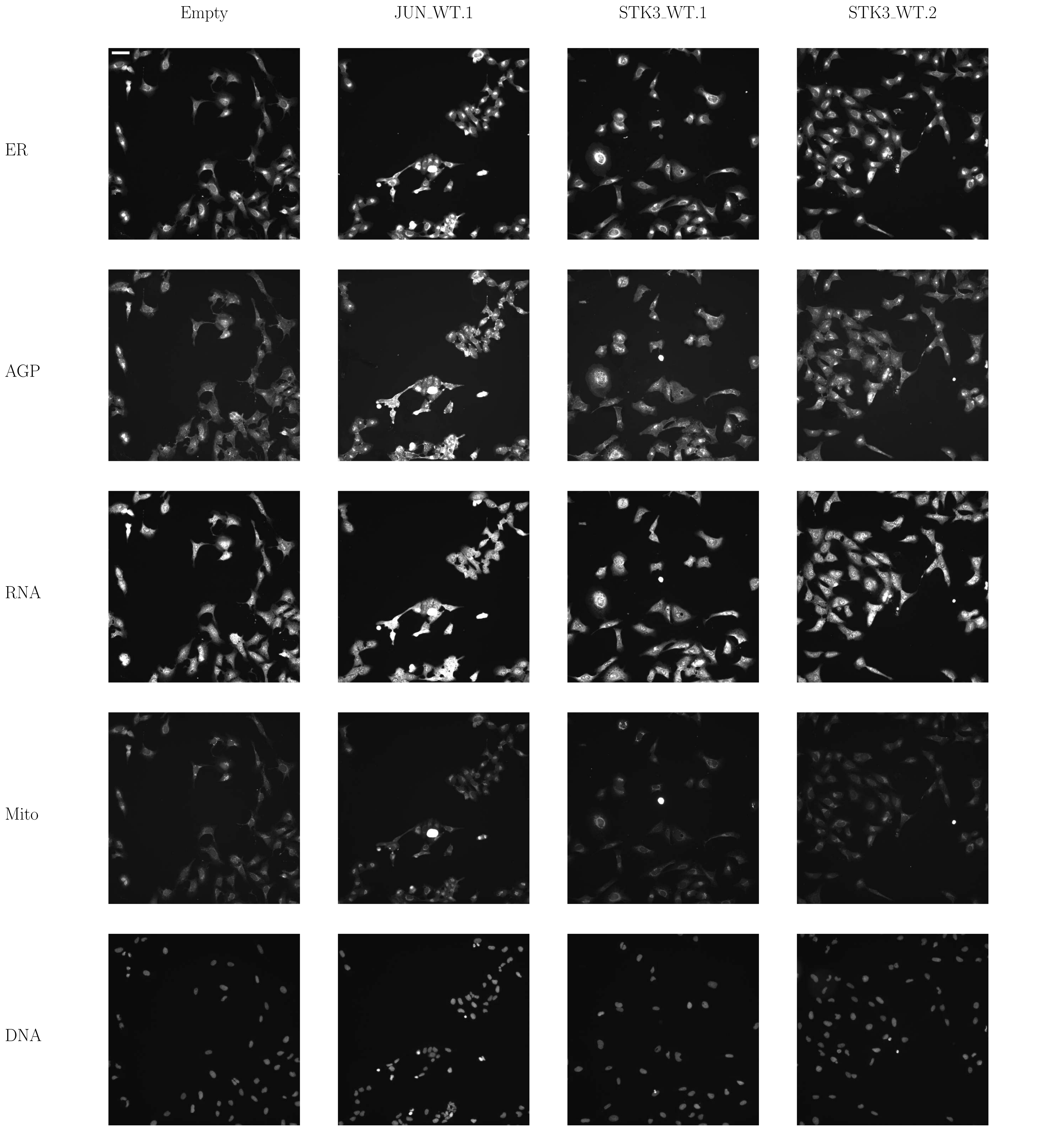

Supplement: Supplementary file 2. — The details of the contents have been described in Figure 5. DOI: http://dx.doi.org/10.7554/eLife.24060.017 [file elife-24060-supp2.zip › Supplementary file 2/type A/15A.pdf]
