# Supplementary material for: Systematic morphological profiling of human gene and allele function via Cell Painting
Source: eLife. 2017 Mar 18;6:e24060. doi: 10.7554/eLife.24060 (PMC5386591; doi:10.7554/eLife.24060)

Cluster 17

a

b

c

How similar is this cluster to the other clusters?

1

Genes in the cluster along with the pathways as annotated by experts

Treatment	Expert Annotation		Cell Count z-score
	Pathway	Regulation Type	
PRKCZ_WT.1	Canonical PKC	Activator	0.03
PRKCZ_WT.2	Canonical PKC	Activator	0.50

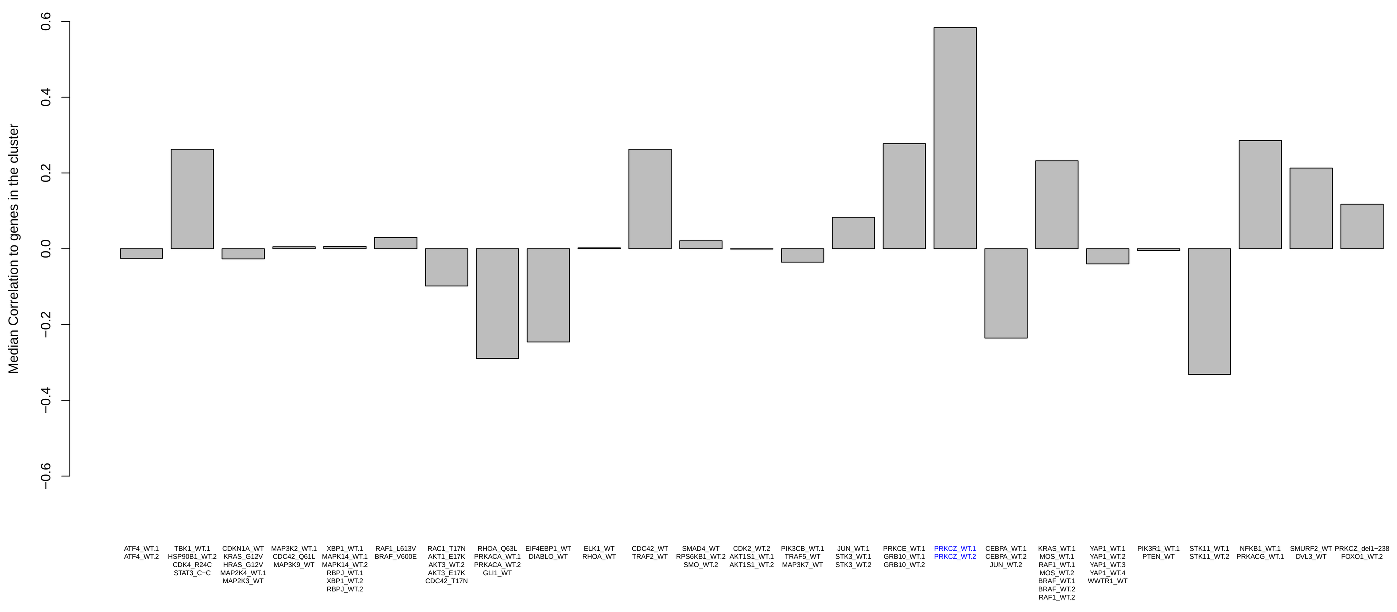

Top 5 genes/alleles negatively correlated to the cluster

Treatment	Expert Annotation		Mean Correlation	Standard Deviation
	Pathway	Regulation Type		
STK11_WT.1	Canonical TOR	Inhibitor	-0.43	0.11
DIABLO_WT	Canonical Apoptosis	Inhibitor	-0.35	0.10
GLI1_WT	Hedgehog	Activator	-0.33	0.15
WWTR1_WT	Canonical Hippo	Inhibitor	-0.32	0.04
PRKACA_WT.2	Canonical PKA	Activator	-0.32	0.22

2

What groups of morphological features are distinguishing in the cluster relative to the untreated samples? (maximum of absolute m-score for the features belonging to the same category; m-score defined as median of a feature z-score across genes in the cluster) Black means no feature is available in the category

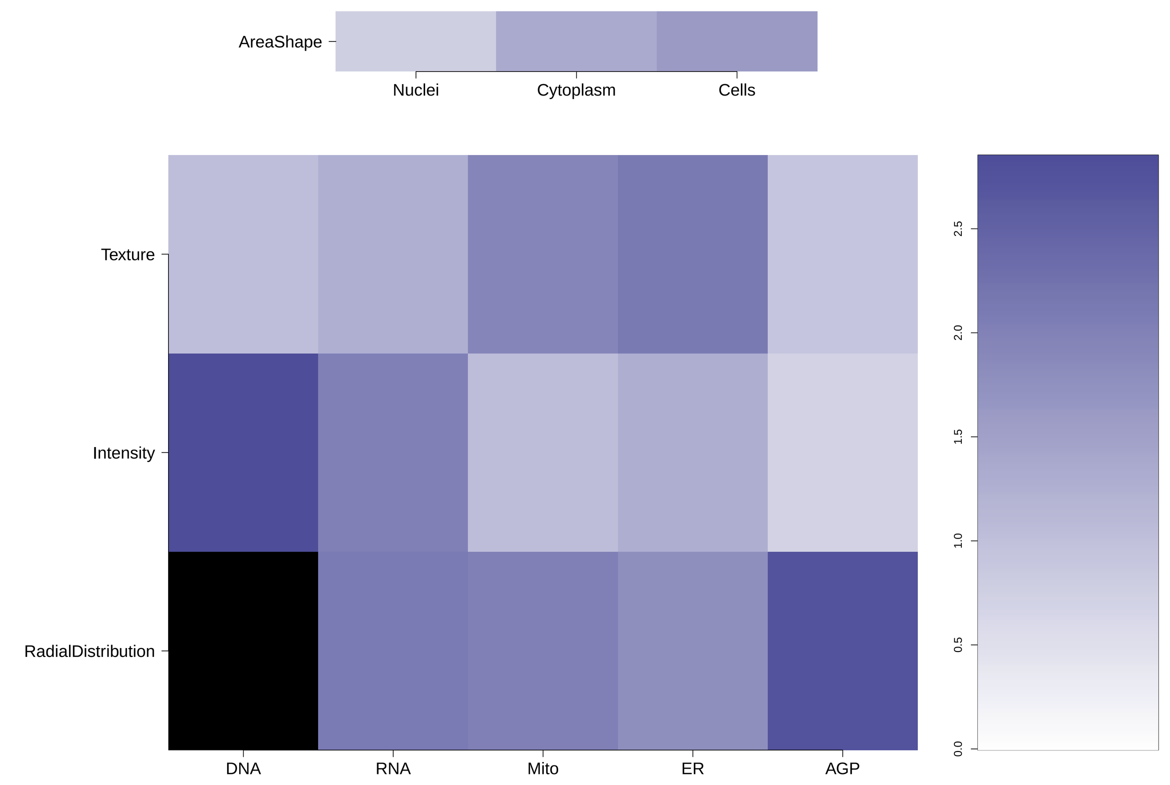

Which individual morphological features are distinguishing in the cluster relative to the untreated samples? Blue/Red means the feature has a positive/negative z-score. Size is proportional to the z-score value.

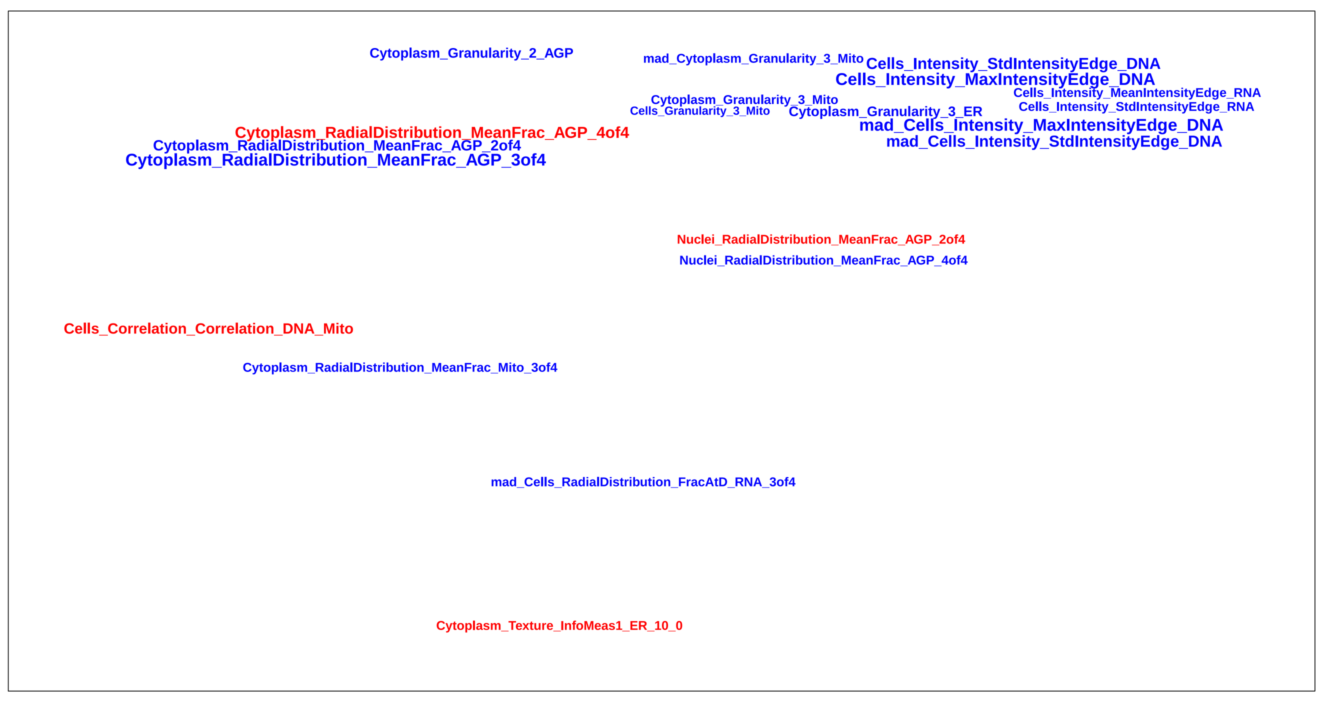

How strongly are genes within the cluster correlated?

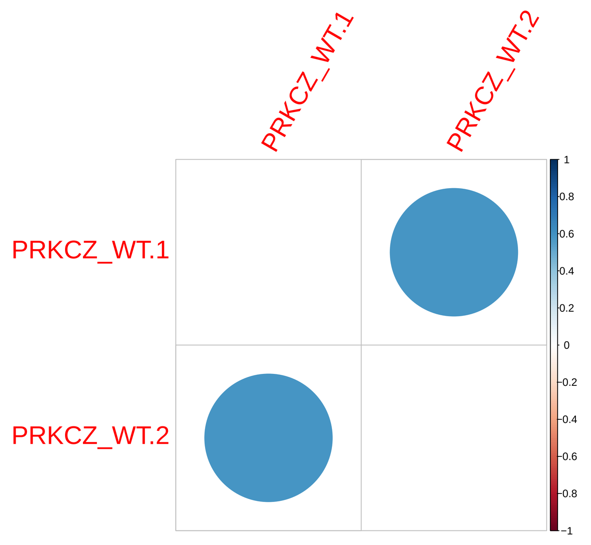

Images show 11% area of a well. Scale bar is 65.60 μm. Plate : 41744 - Genes in the Cluster (Channels are sorted based on their dominance in the grid plot)

3

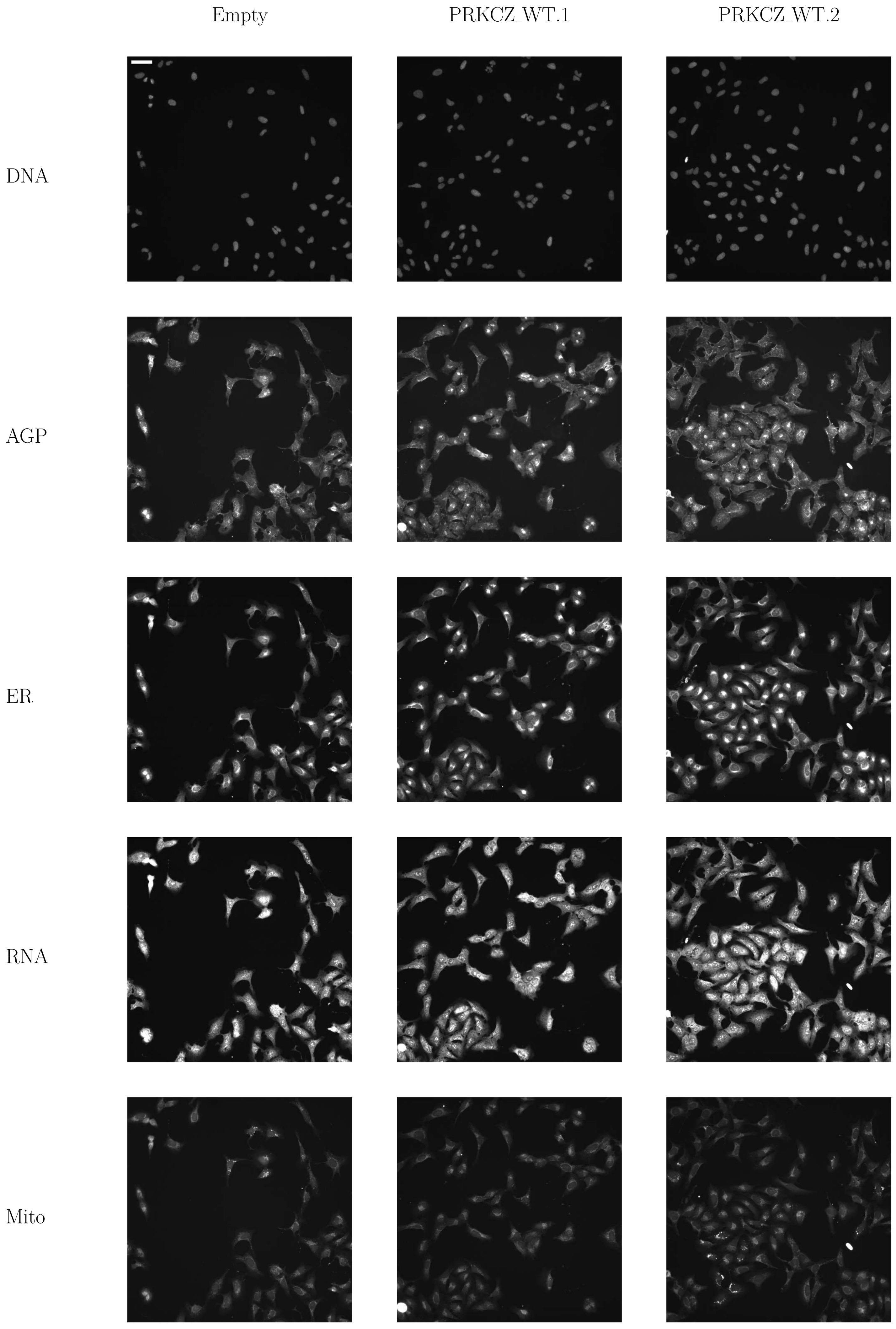

Supplement: Supplementary file 2. — The details of the contents have been described in Figure 5. DOI: http://dx.doi.org/10.7554/eLife.24060.017 [file elife-24060-supp2.zip › Supplementary file 2/type A/17A.pdf]
