# Supplementary material for: Systematic morphological profiling of human gene and allele function via Cell Painting
Source: eLife. 2017 Mar 18;6:e24060. doi: 10.7554/eLife.24060 (PMC5386591; doi:10.7554/eLife.24060)

Cluster 2

a | b | c

How similar is this cluster to the other clusters?

1

Genes in the cluster along with the pathways as annotated by experts

	Expert Annotation		
Treatment	Pathway	Regulation Type	Cell Count z-score
CDK4_R24C	Canonical Cell Cycle	Activator	-0.43
HSP90B1_WT.2	Canonical ER Stress/UPR	Activator	-0.22
STAT3_C-C	Canonical JAK/STAT	Activator	-0.59
TBK1_WT.1	Canonical NFkB	Activator	-0.63

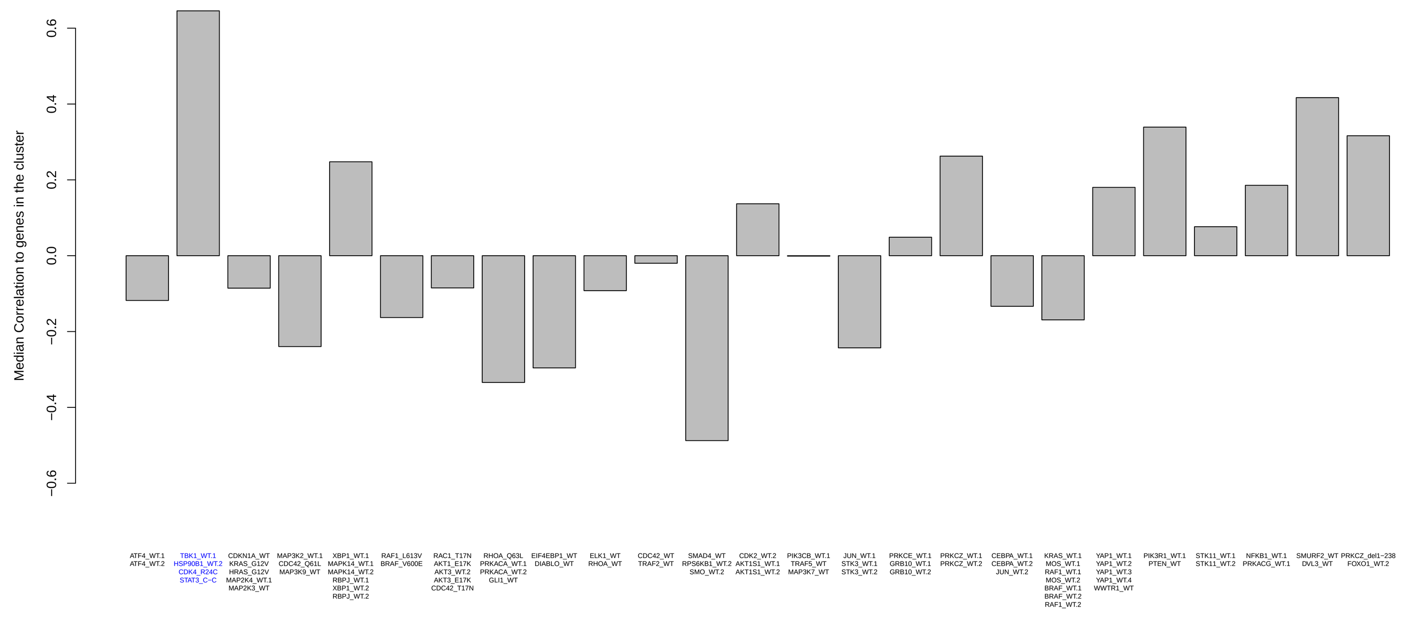

Top 5 genes/alleles negatively correlated to the cluster

	Expert Annotation			
Treatment	Pathway	Regulation Type	Mean Correlation	Standard Deviation
SMAD4_WT	Canonical SMAD	Activator	-0.59	0.06
RPS6KB1_WT.2	Canonical TOR	Activator	-0.43	0.12
PRKACA_WT.2	Canonical PKA	Activator	-0.40	0.09
STK3_WT.2	Canonical Hippo	Activator	-0.40	0.12
DDIT3_WT.2	Canonical ER Stress/UPR	Activator	-0.36	0.11

2

What groups of morphological features are distinguishing in the cluster relative to the untreated samples? (maximum of absolute m-score for the features belonging to the same category; m-score defined as median of a feature z-score across genes in the cluster) Black means no feature is available in the category

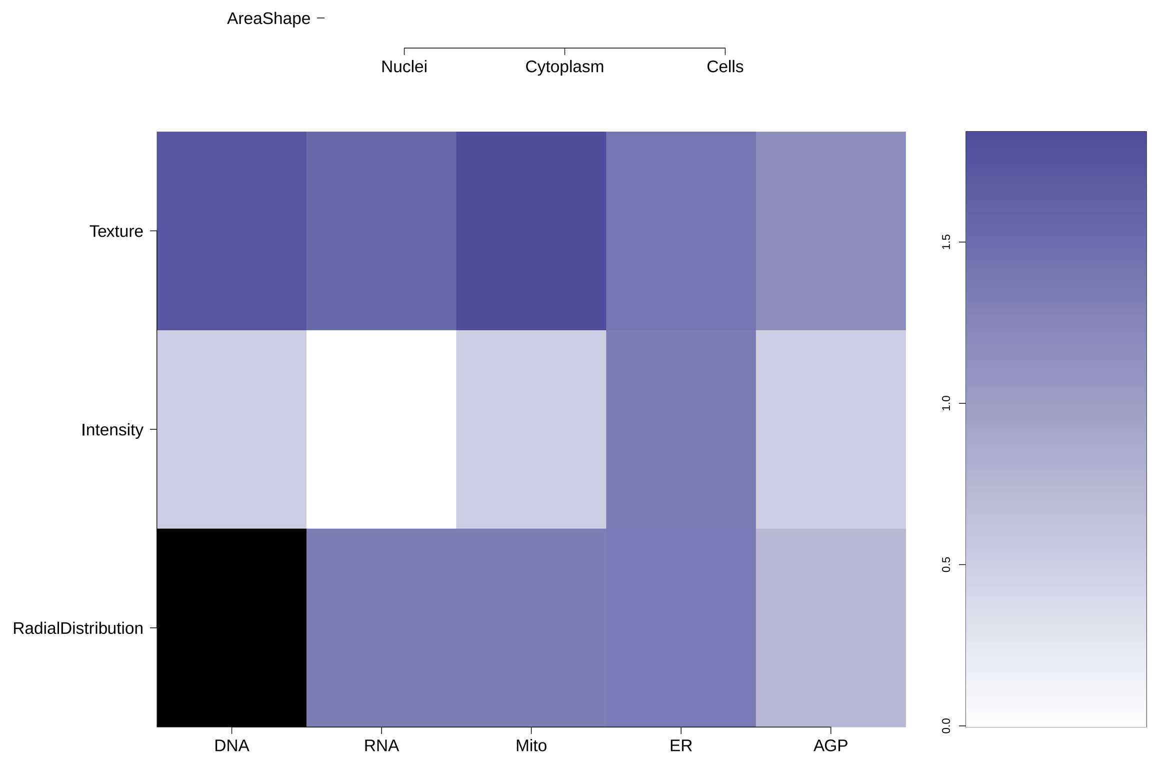

Which individual morphological features are distinguishing in the cluster relative to the untreated samples? Blue/Red means the feature has a positive/negative z-score. Size is proportional to the z-score value.

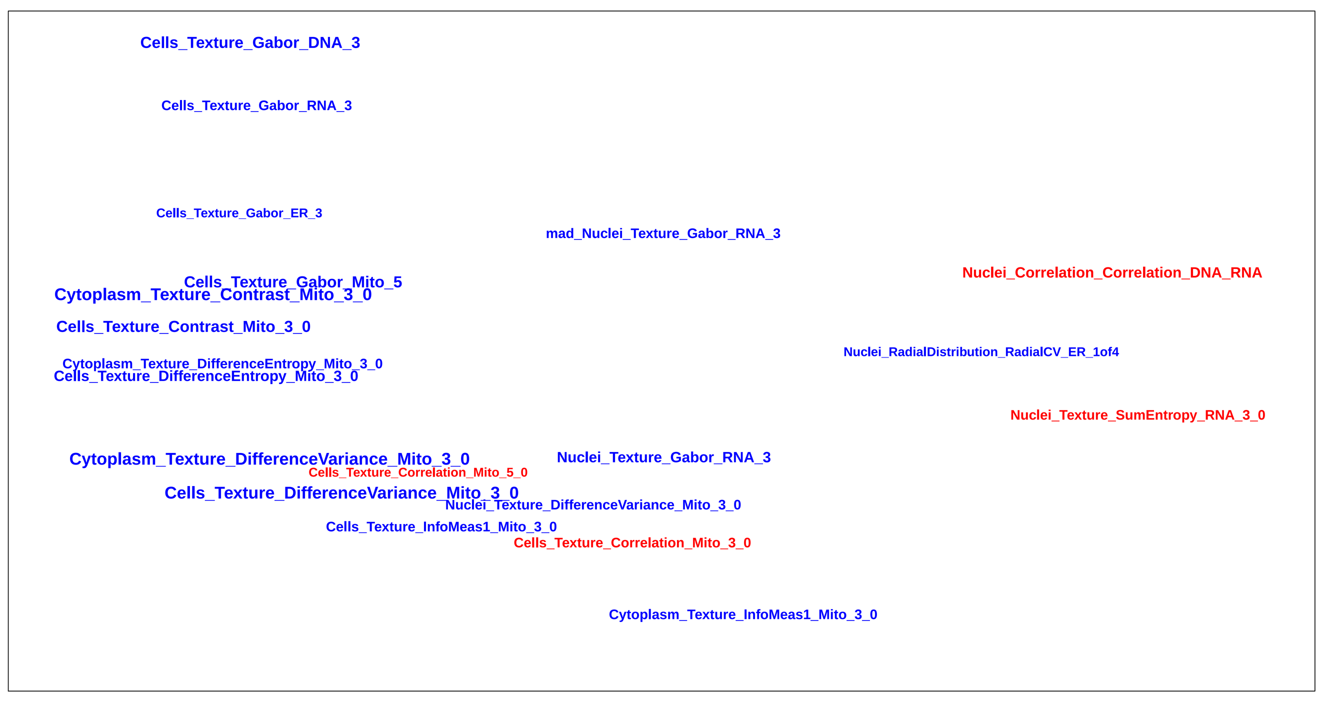

How strongly are genes within the cluster correlated?

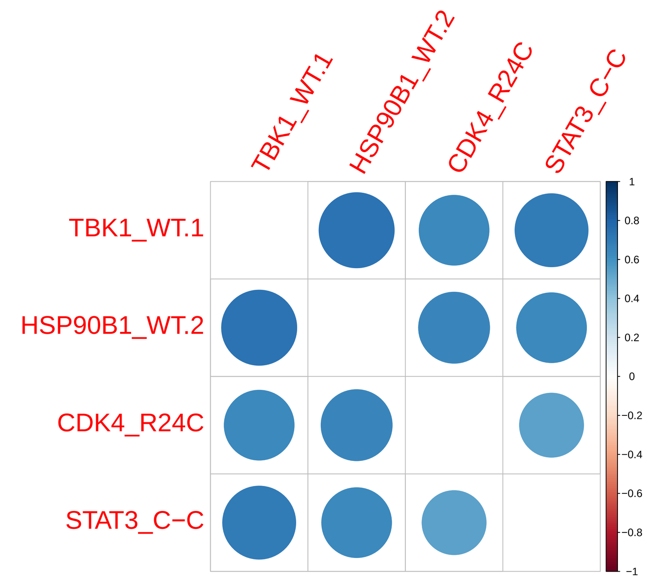

3

Images show 11% area of a well. Scale bar is 65.60 μm. Plate : 41744 - Genes in the Cluster (Channels are sorted based on their dominance in the grid plot)

Empty | TBK1_WT.1 | HSP90B1_WT.2 | CDK4_R24C | STAT3_C-C

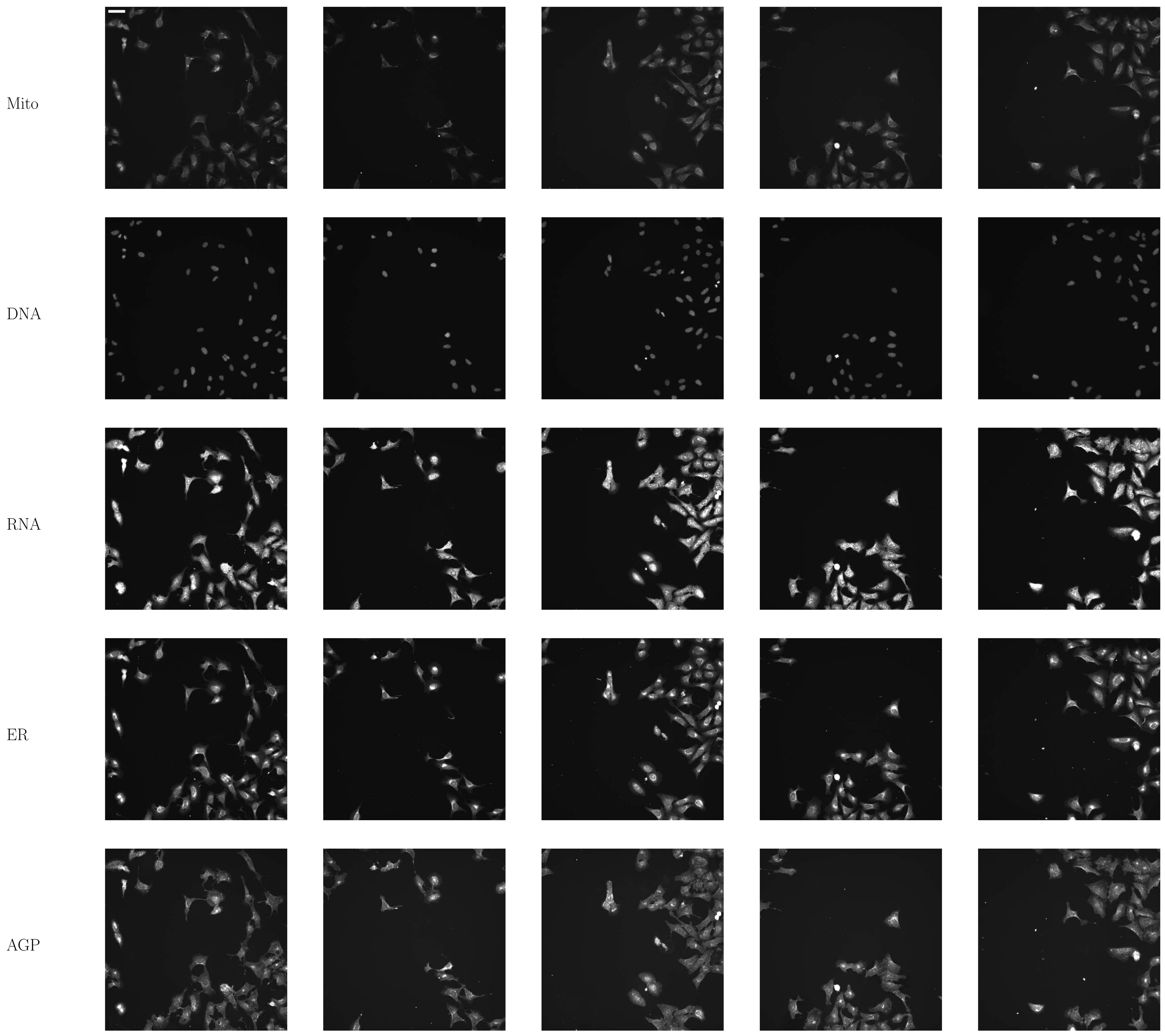

Supplement: Supplementary file 2. — The details of the contents have been described in Figure 5. DOI: http://dx.doi.org/10.7554/eLife.24060.017 [file elife-24060-supp2.zip › Supplementary file 2/type A/2A.pdf]
