# Supplementary material for: Systematic morphological profiling of human gene and allele function via Cell Painting
Source: eLife. 2017 Mar 18;6:e24060. doi: 10.7554/eLife.24060 (PMC5386591; doi:10.7554/eLife.24060)
Supplement: Supplementary file 2. — The details of the contents have been described in Figure 5. DOI: http://dx.doi.org/10.7554/eLife.24060.017 [file elife-24060-supp2.zip › Supplementary file 2/type B/20B.pdf]

| | Expert Annotation | |
|---|---|---|
| Treatment | Pathway | Regulation Type |
| WWTR1_WT | Canonical Hippo | Inhibitor |
| YAP1_WT.1 | Canonical Hippo | Inhibitor |
| YAP1_WT.2 | Canonical Hippo | Inhibitor |
| YAP1_WT.3 | Canonical Hippo | Inhibitor |
| YAP1_WT.4 | Canonical Hippo | Inhibitor |

Nuclei_Intensity_IntegratedIntensity_DNA

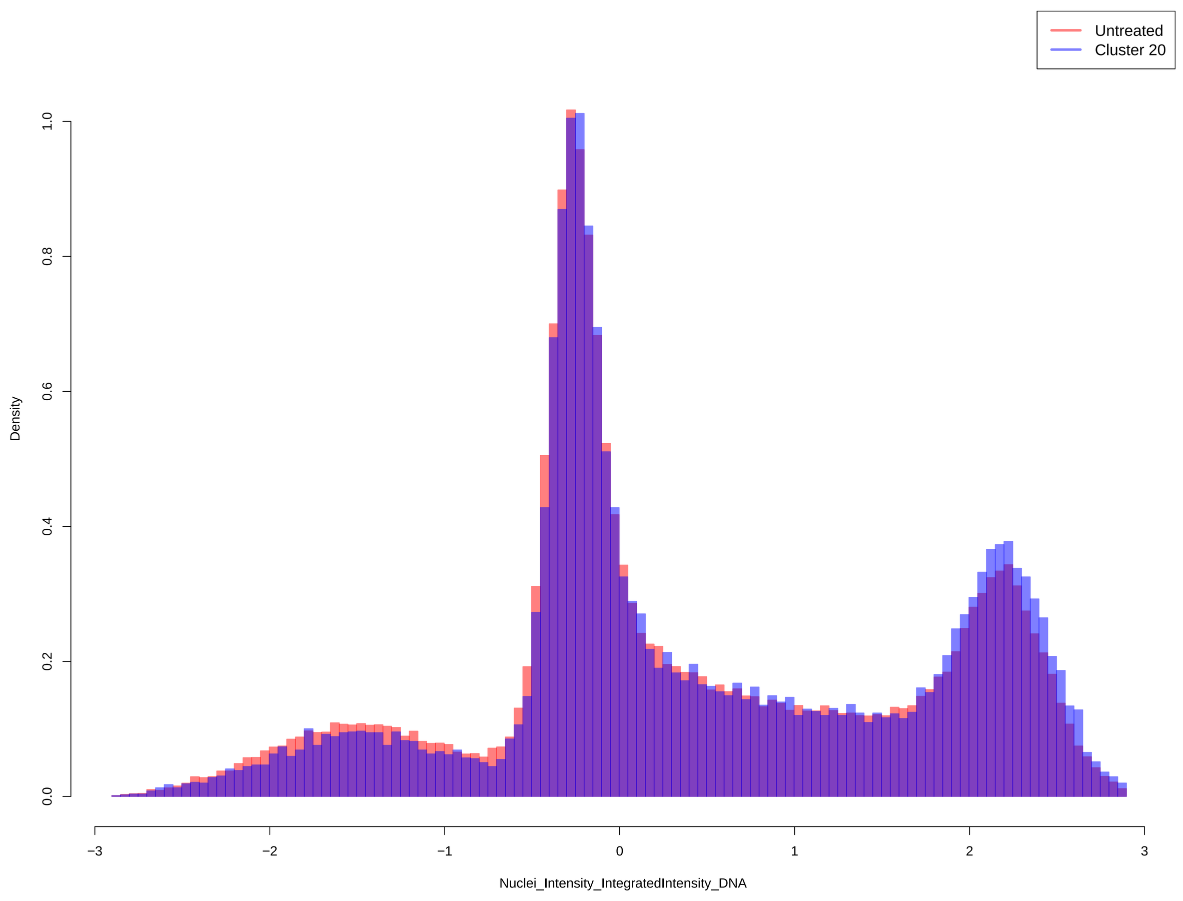


Different categories of cells in the cluster :

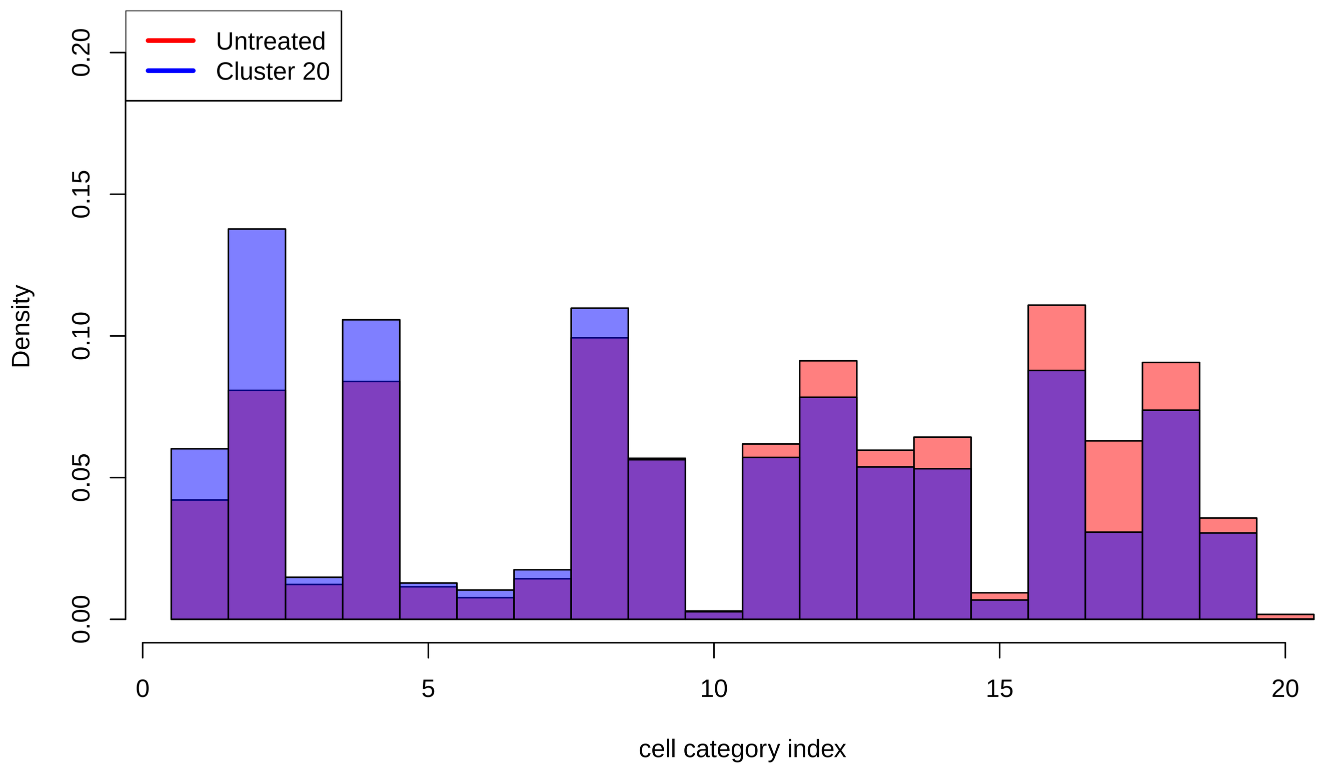


Different categories of cells in single genes in the cluster :

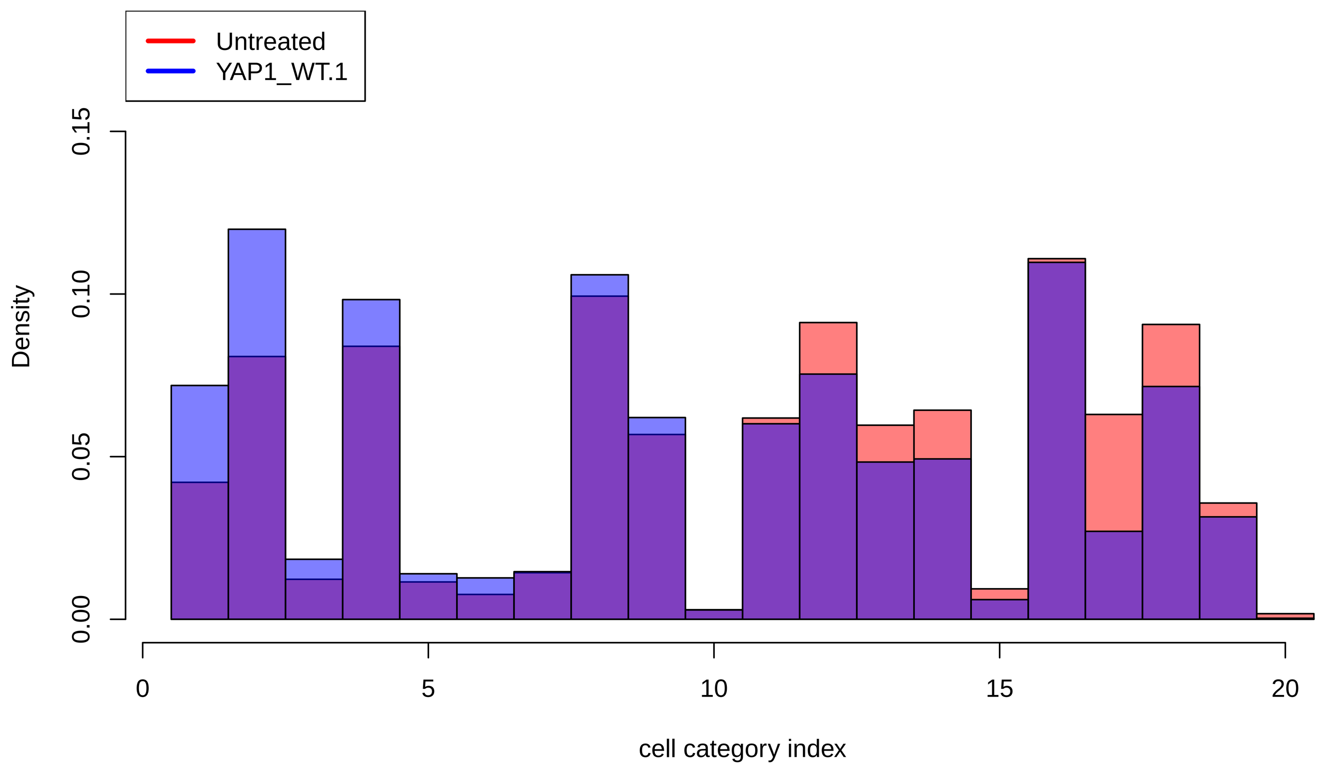


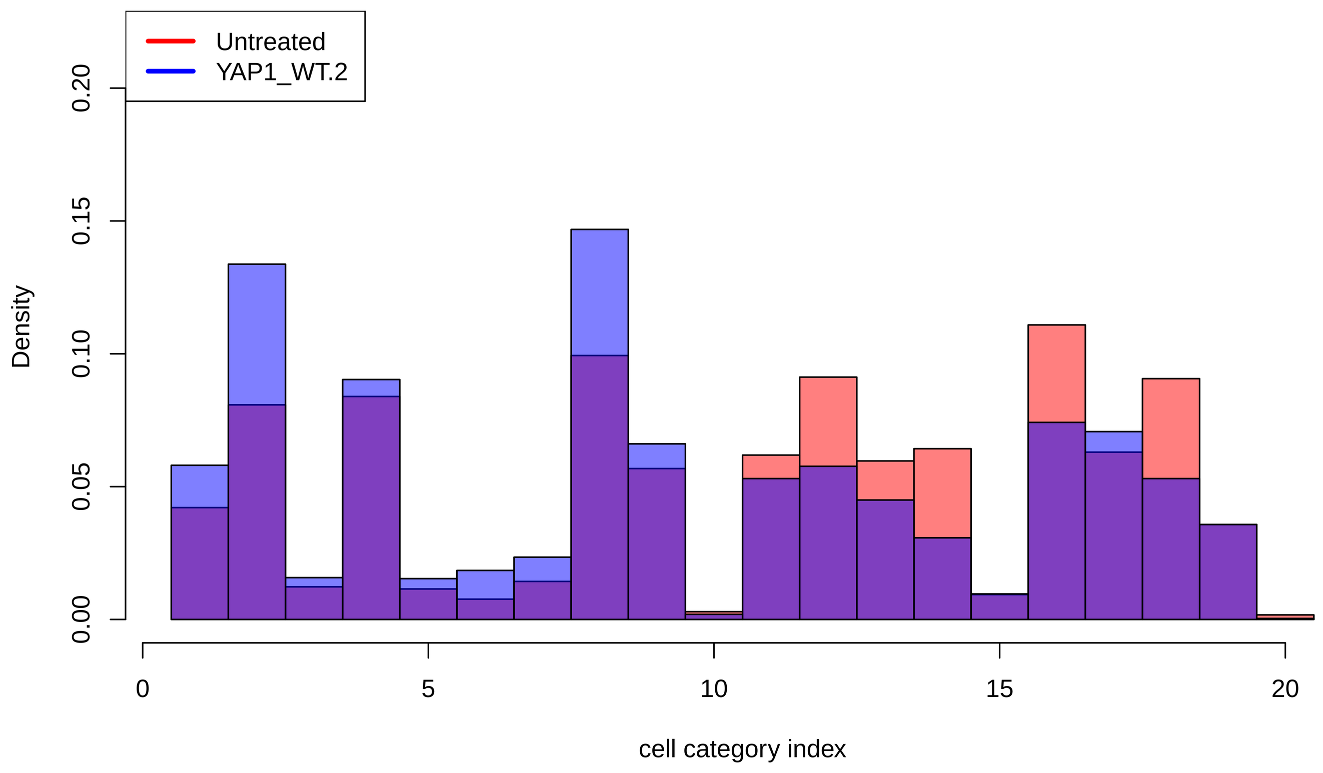


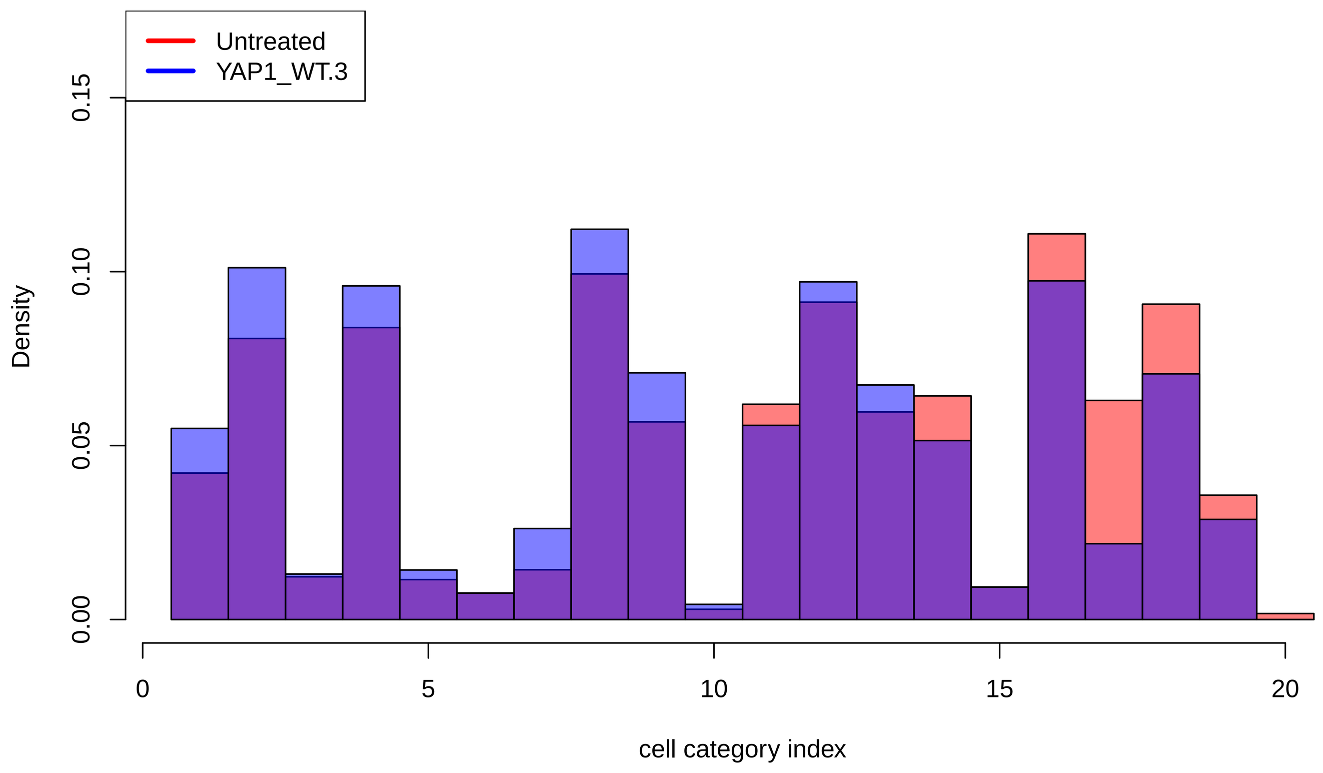

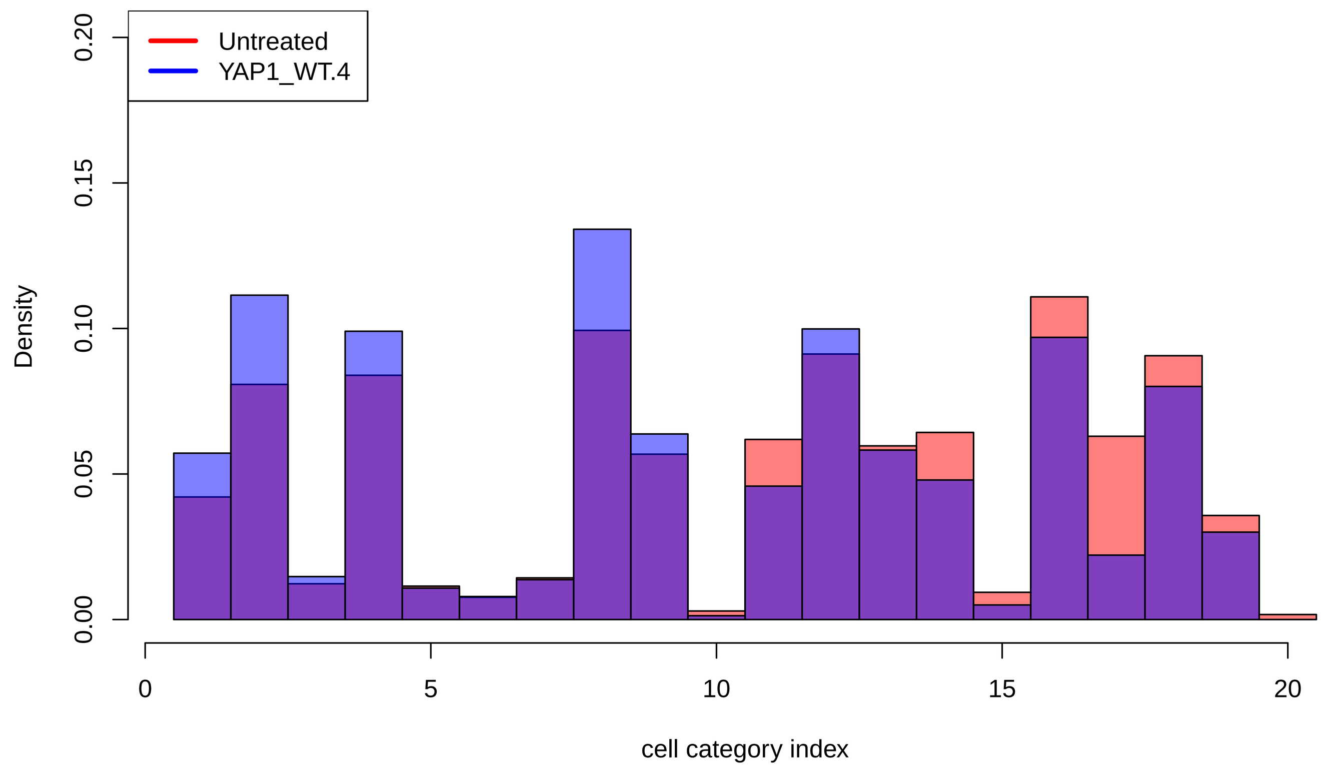
Untreated
YAP1_WT.4
Density
0.00
0.05
0.10
0.15
0.20
0
5
10
15
20
cell category index

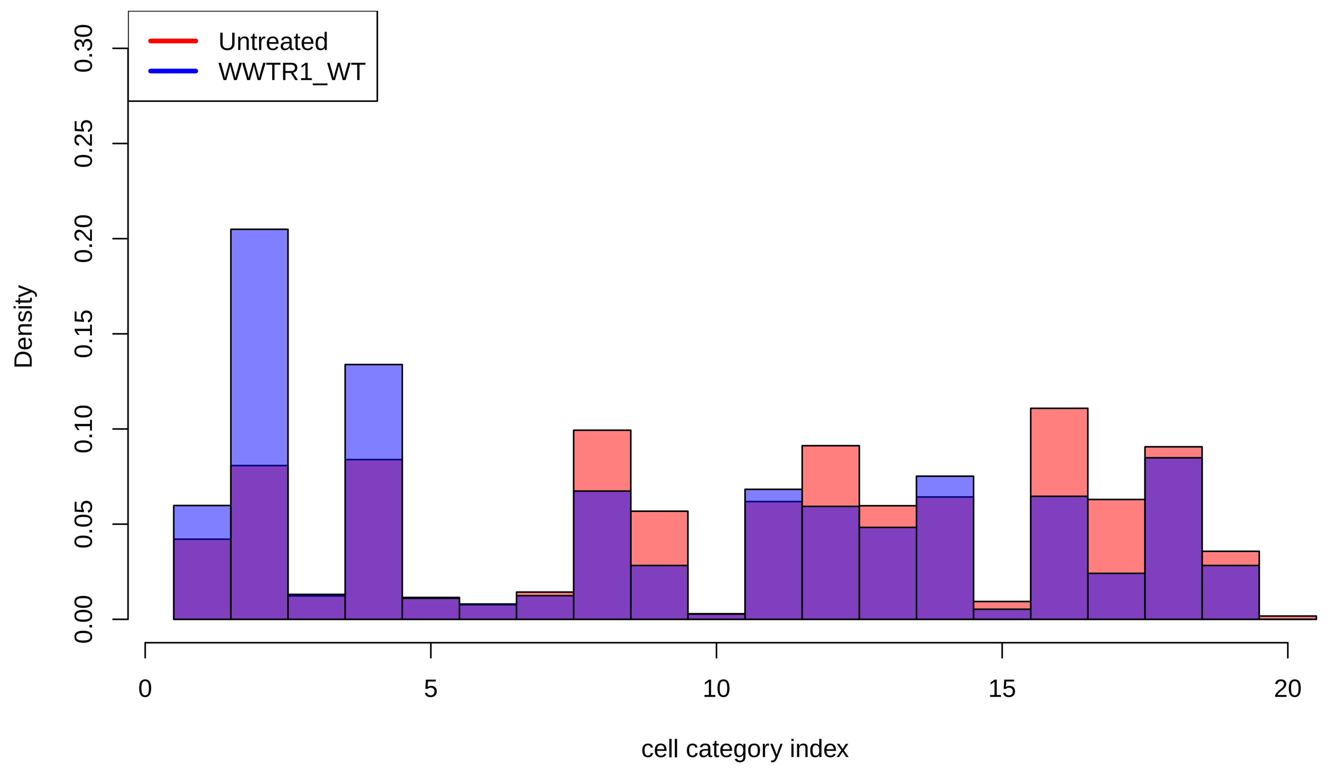
Untreated
WWTR1_WT
Density
0.00
0.05
0.10
0.15
0.20
0.25
0.30
0
5
10
15
20
cell category index

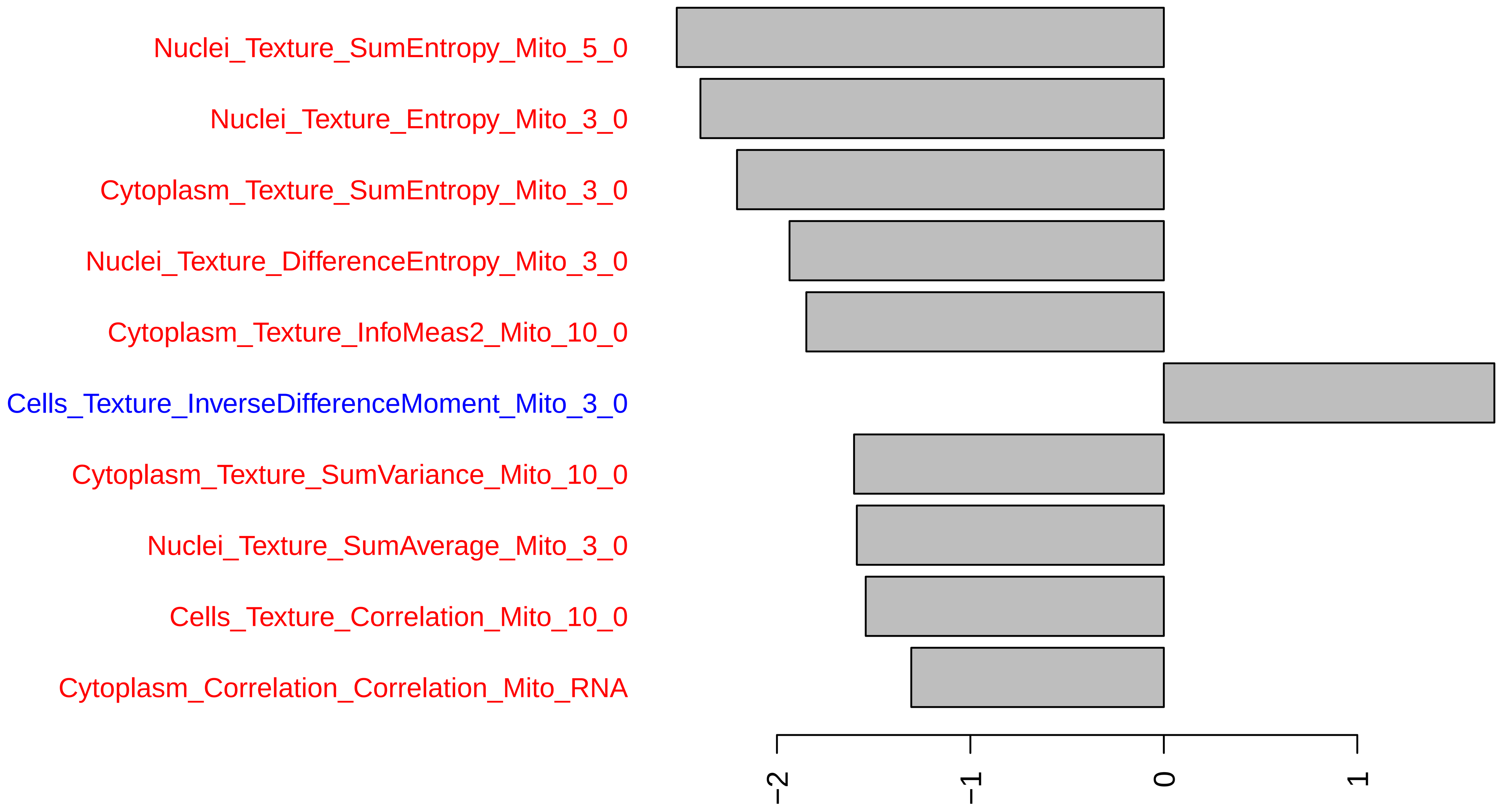


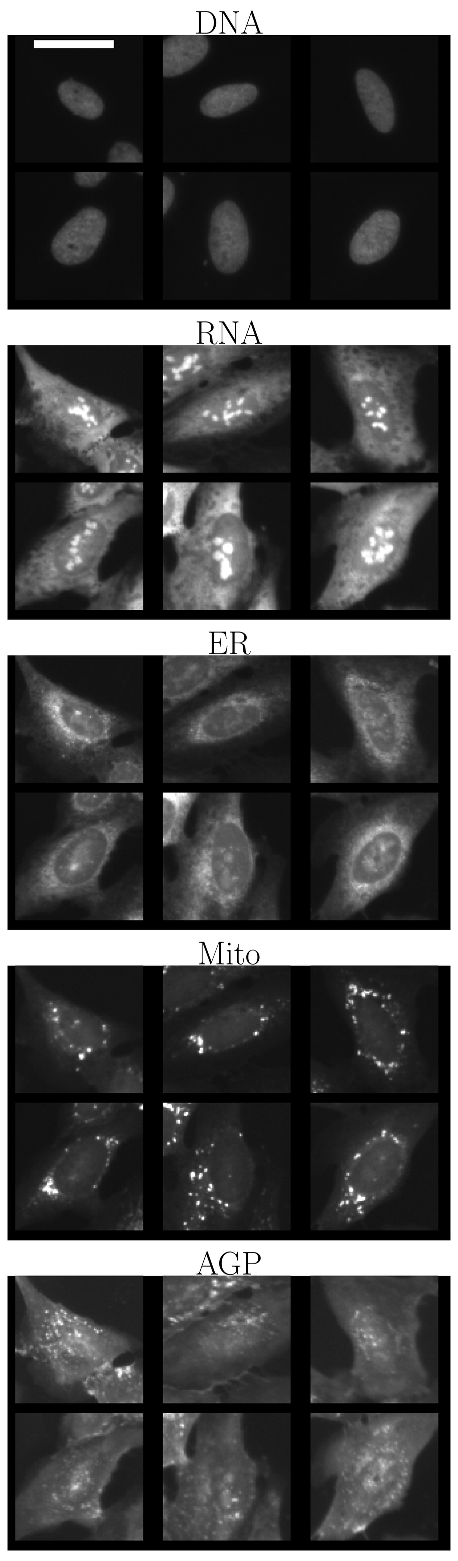


Note : Scale bar is 39.36 $\mu$m.

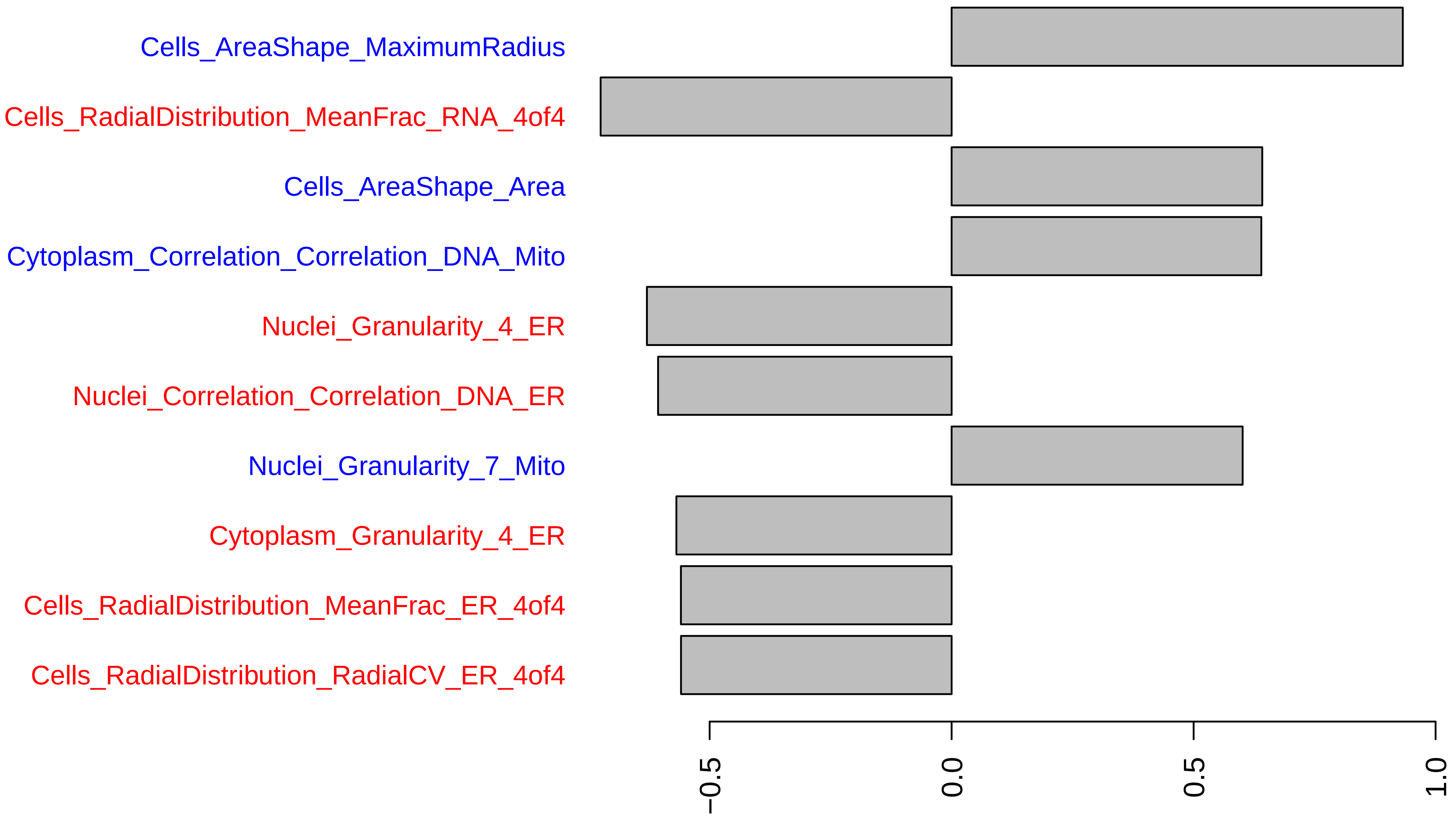


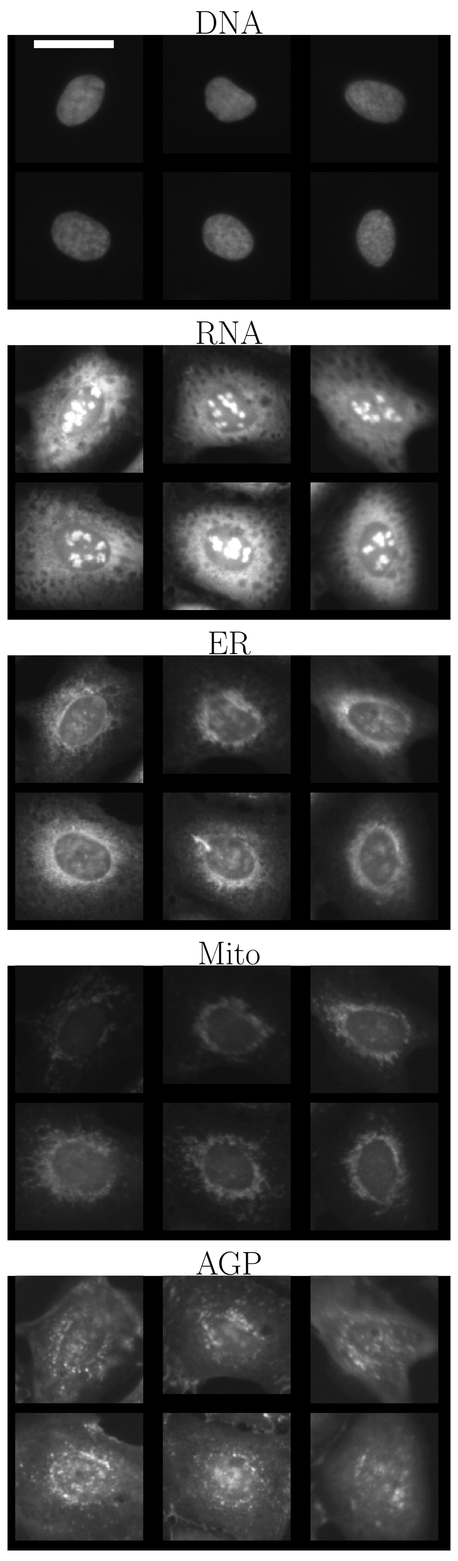


Note : Scale bar is 39.36 $\mu$m.

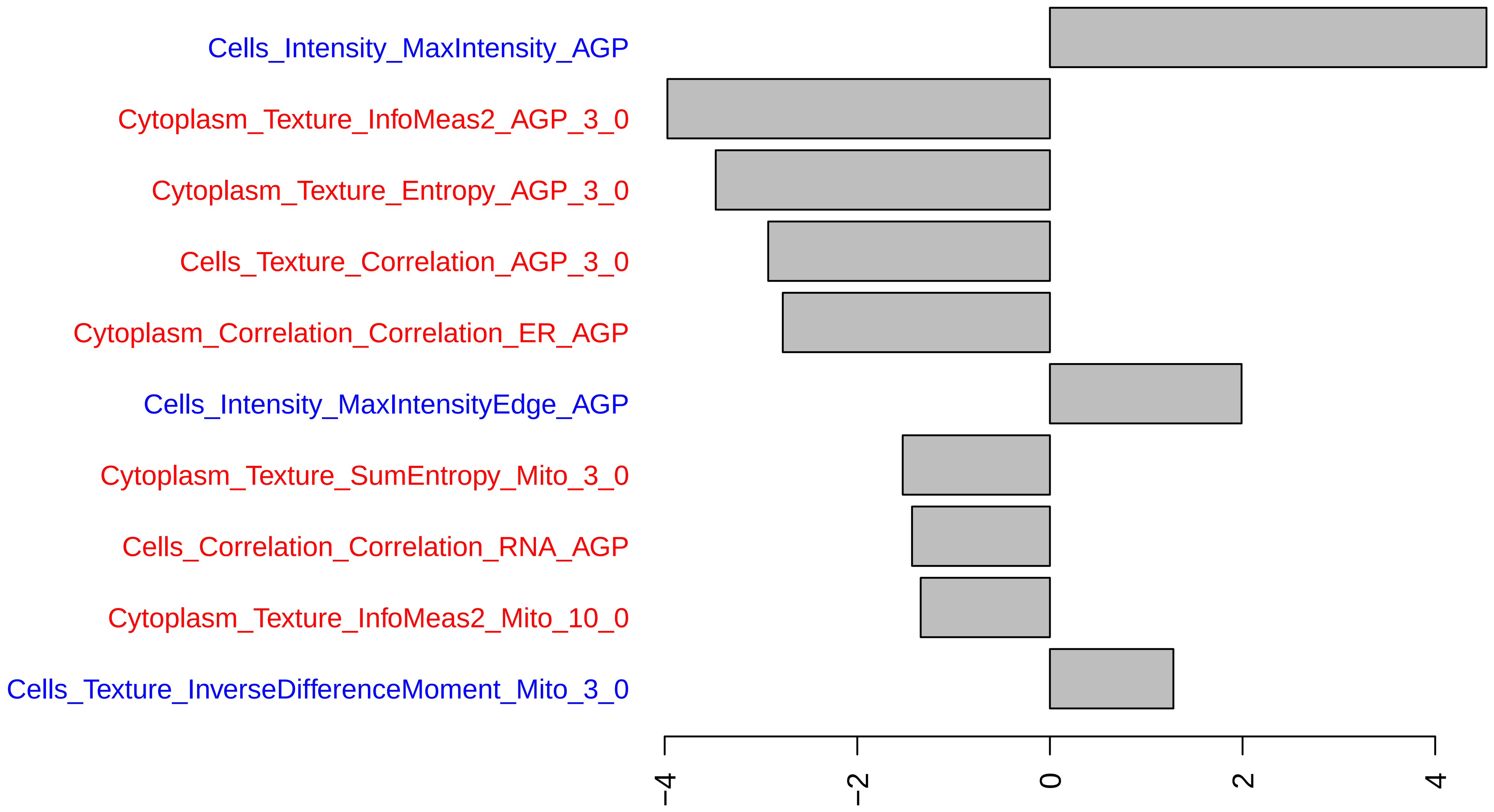


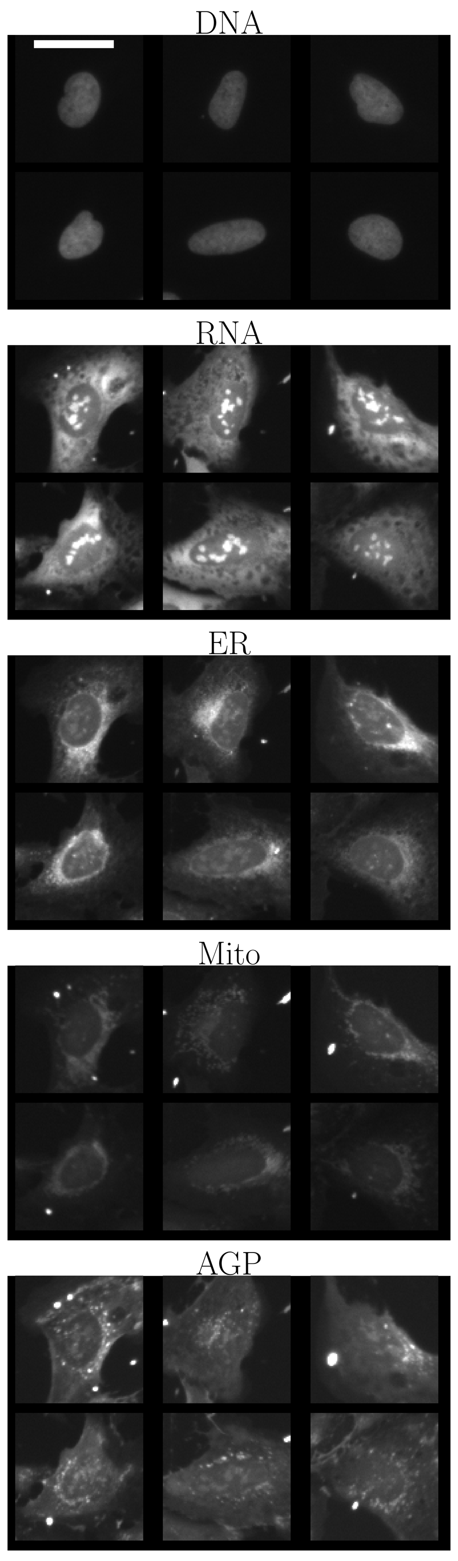


Note : Scale bar is 39.36 $\mu$m.

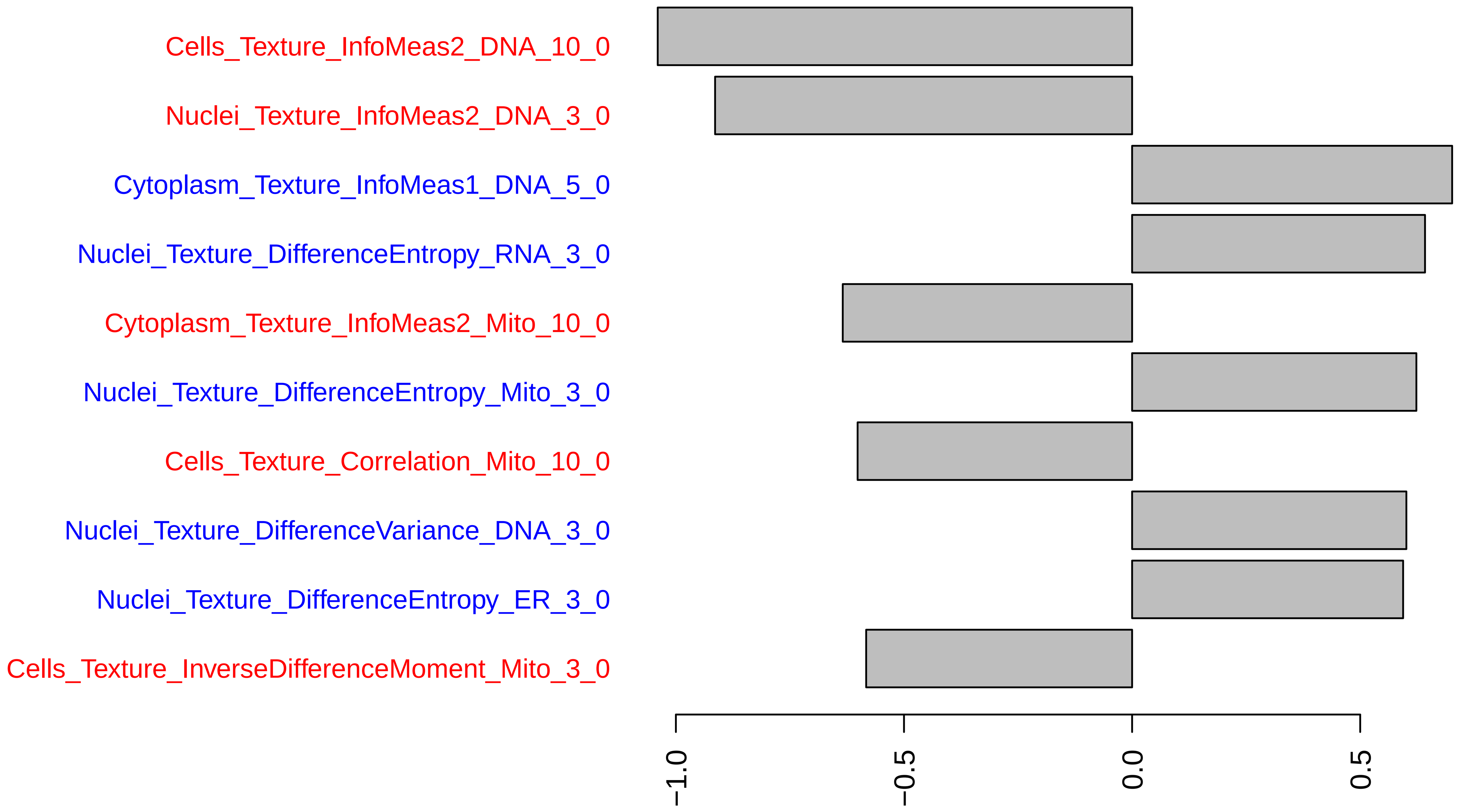


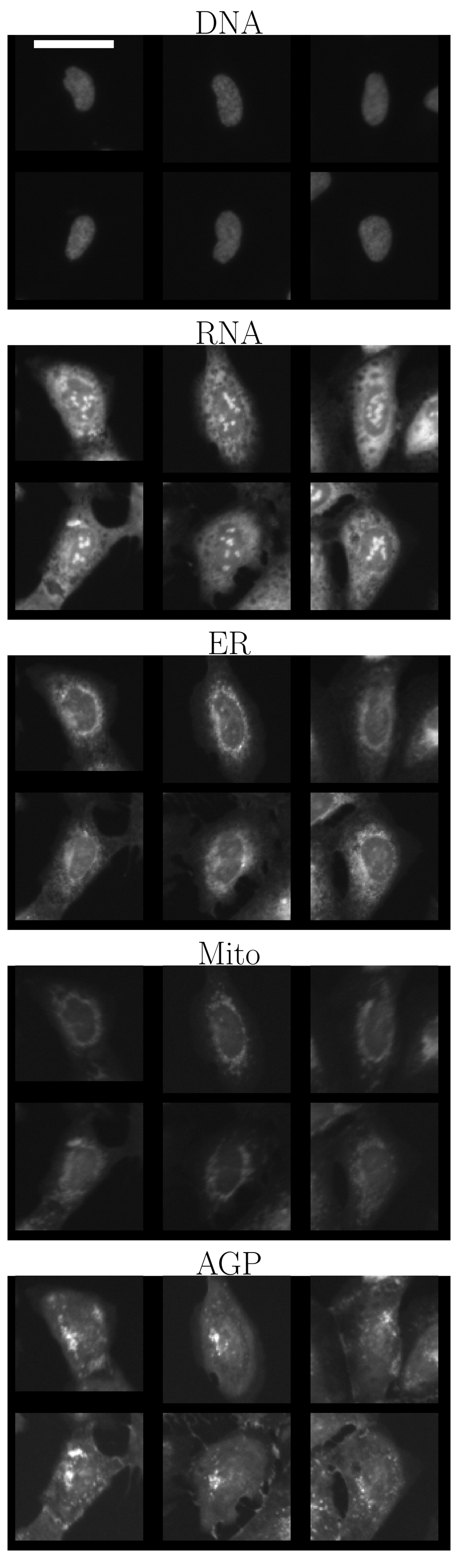


Note : Scale bar is 39.36 $\mu$m.

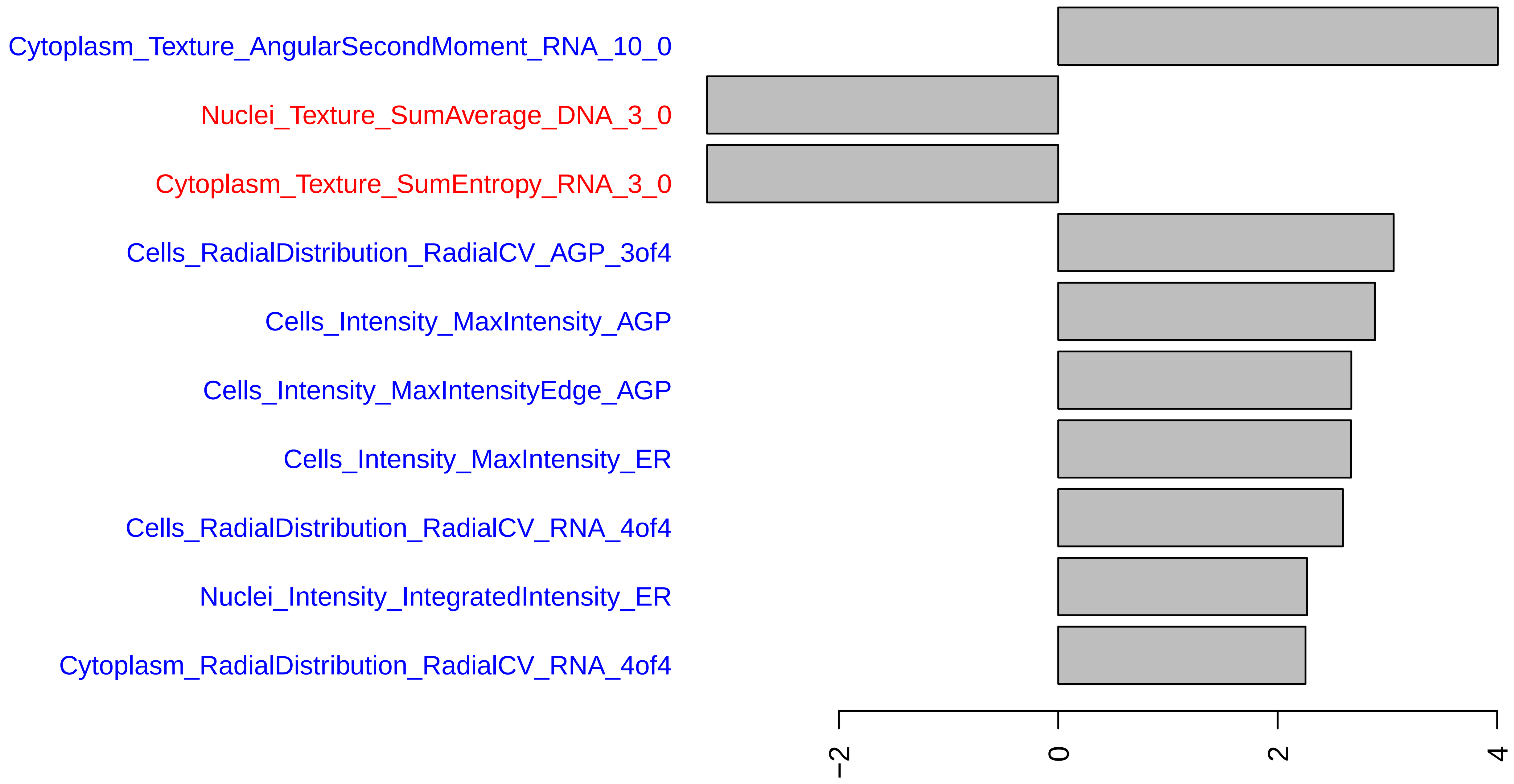


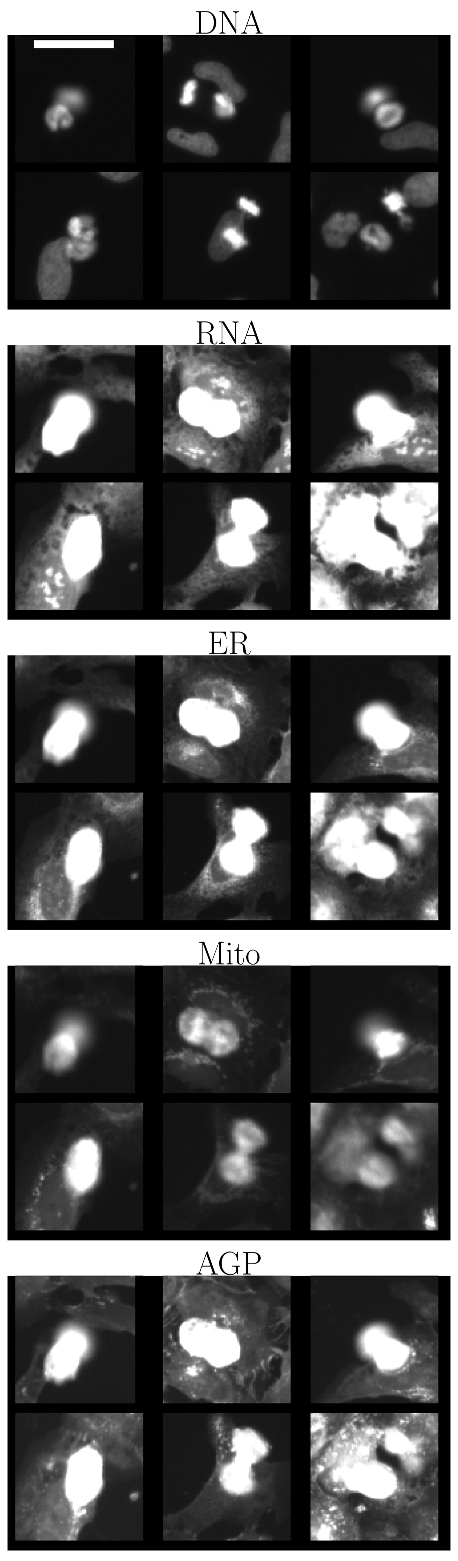


Note : Scale bar is 39.36 $\mu$m.

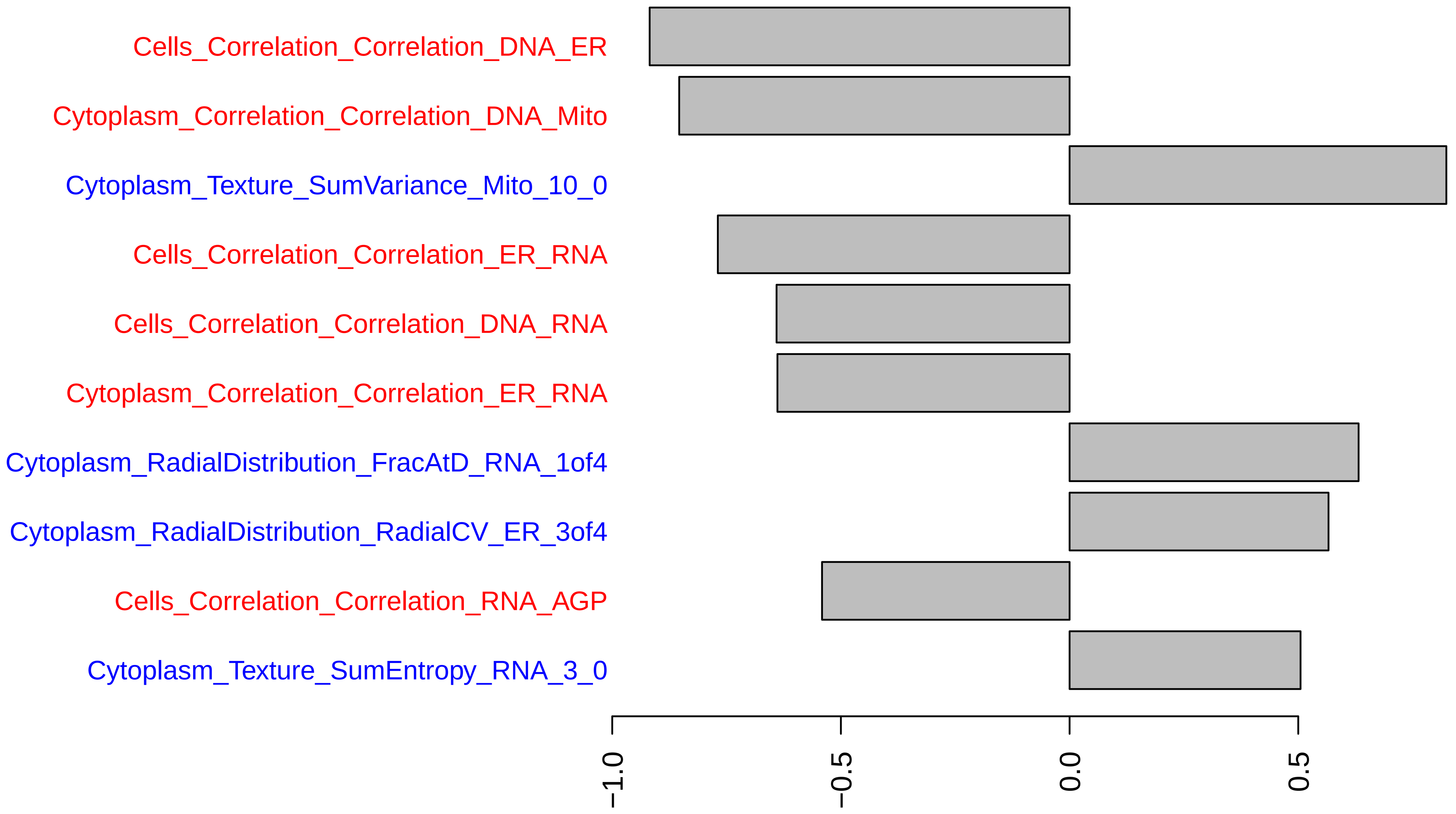


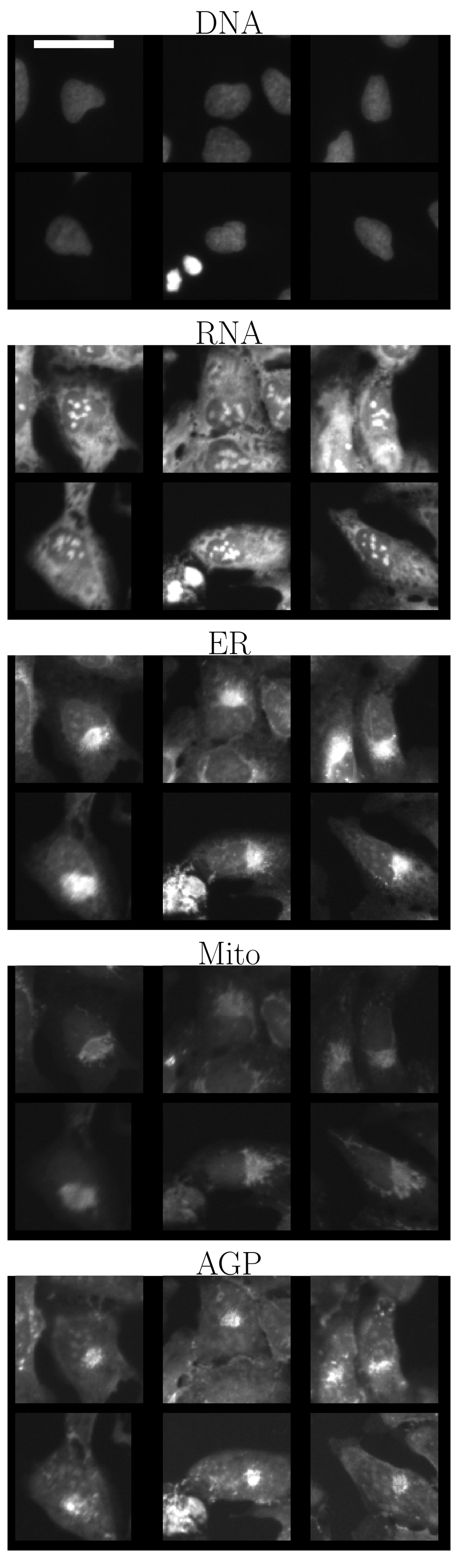


Note : Scale bar is 39.36 $\mu$m.

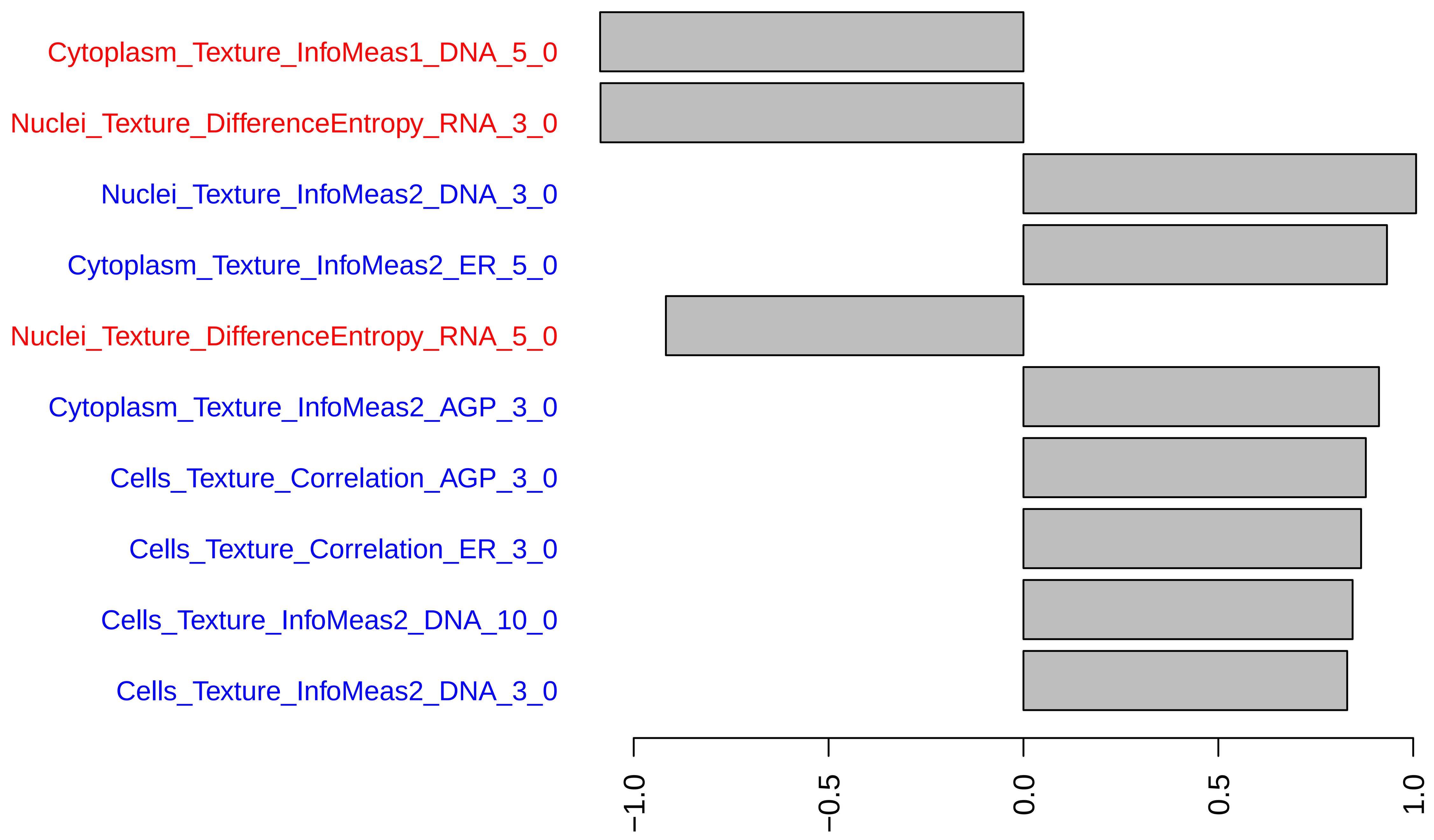


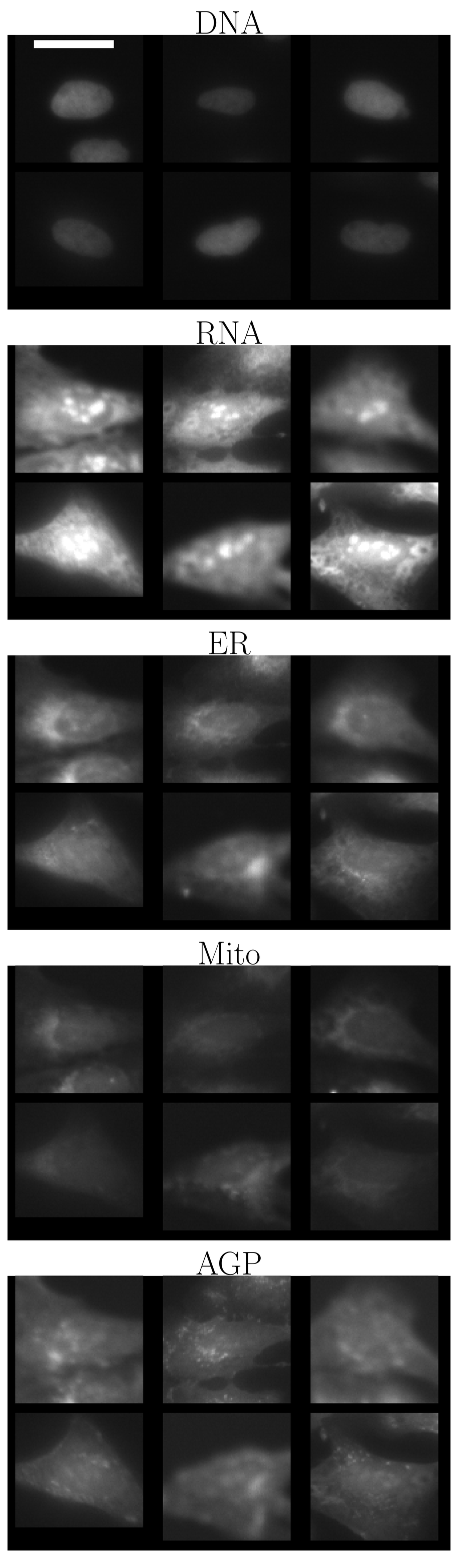


Note : Scale bar is 39.36 $\mu$m.

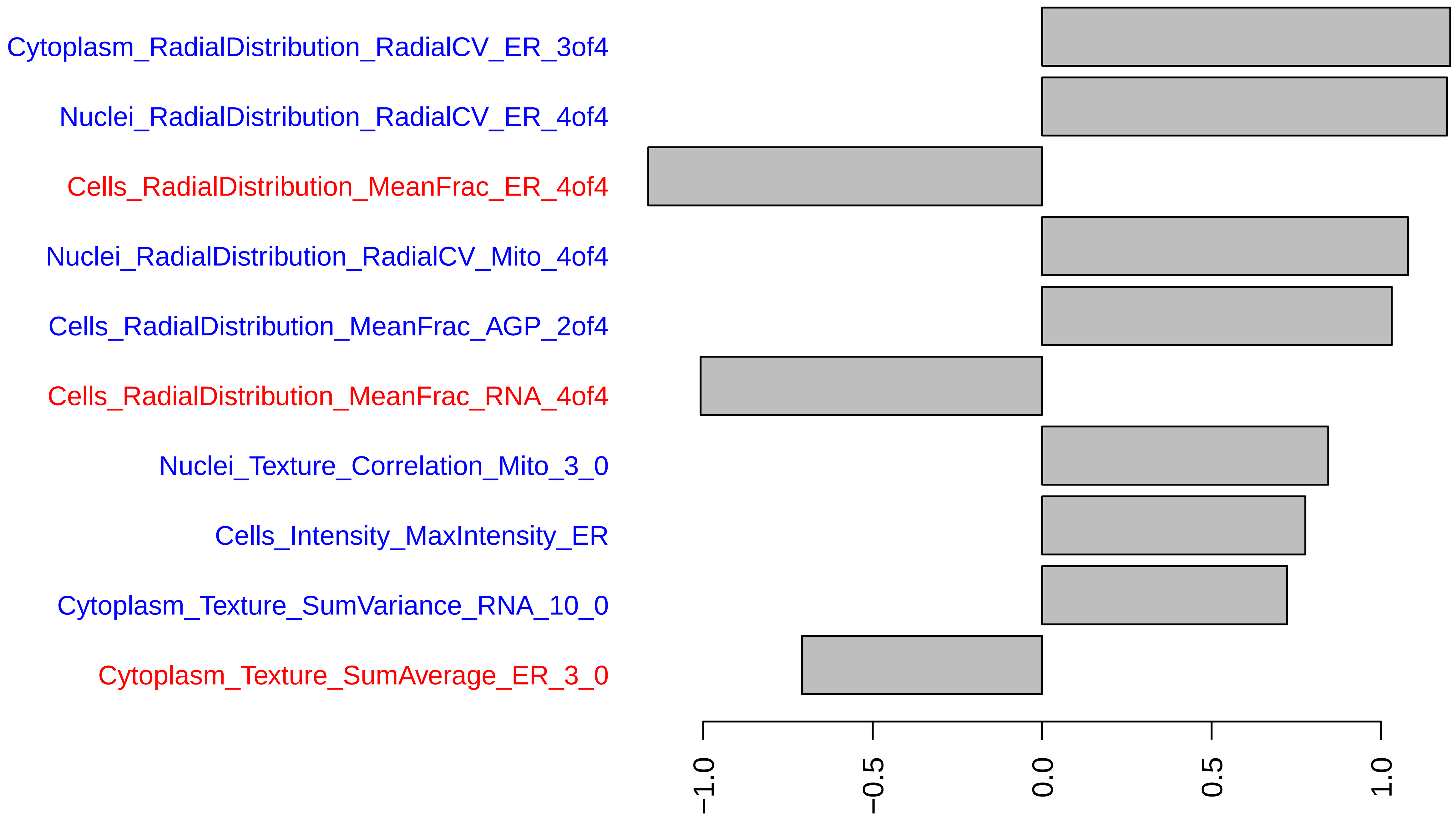


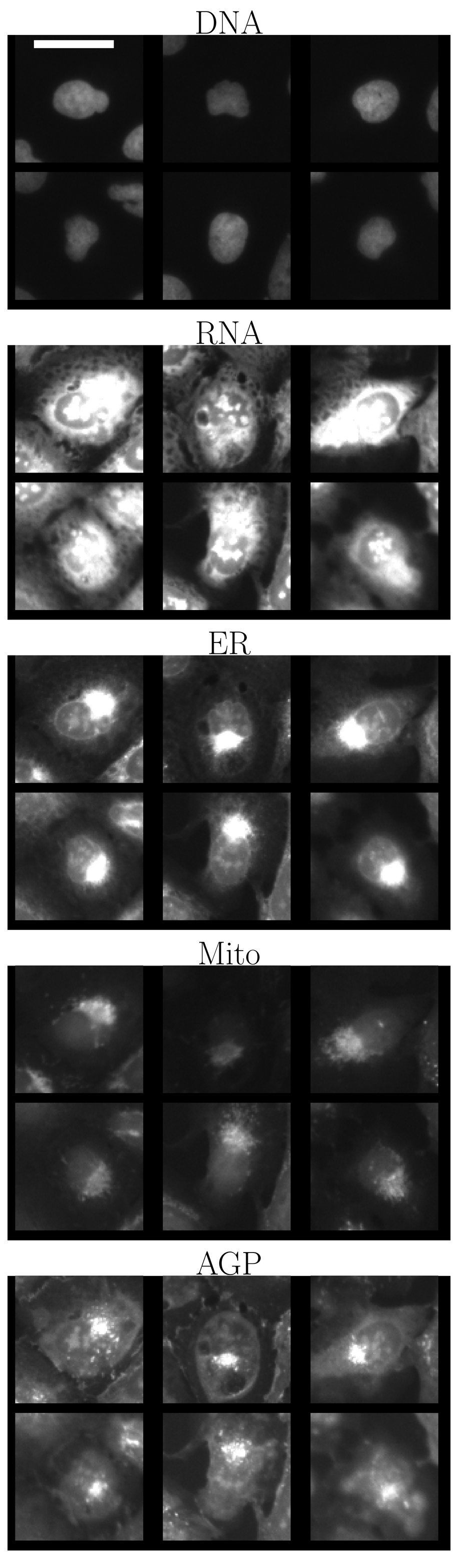


Note : Scale bar is 39.36 $\mu$m.

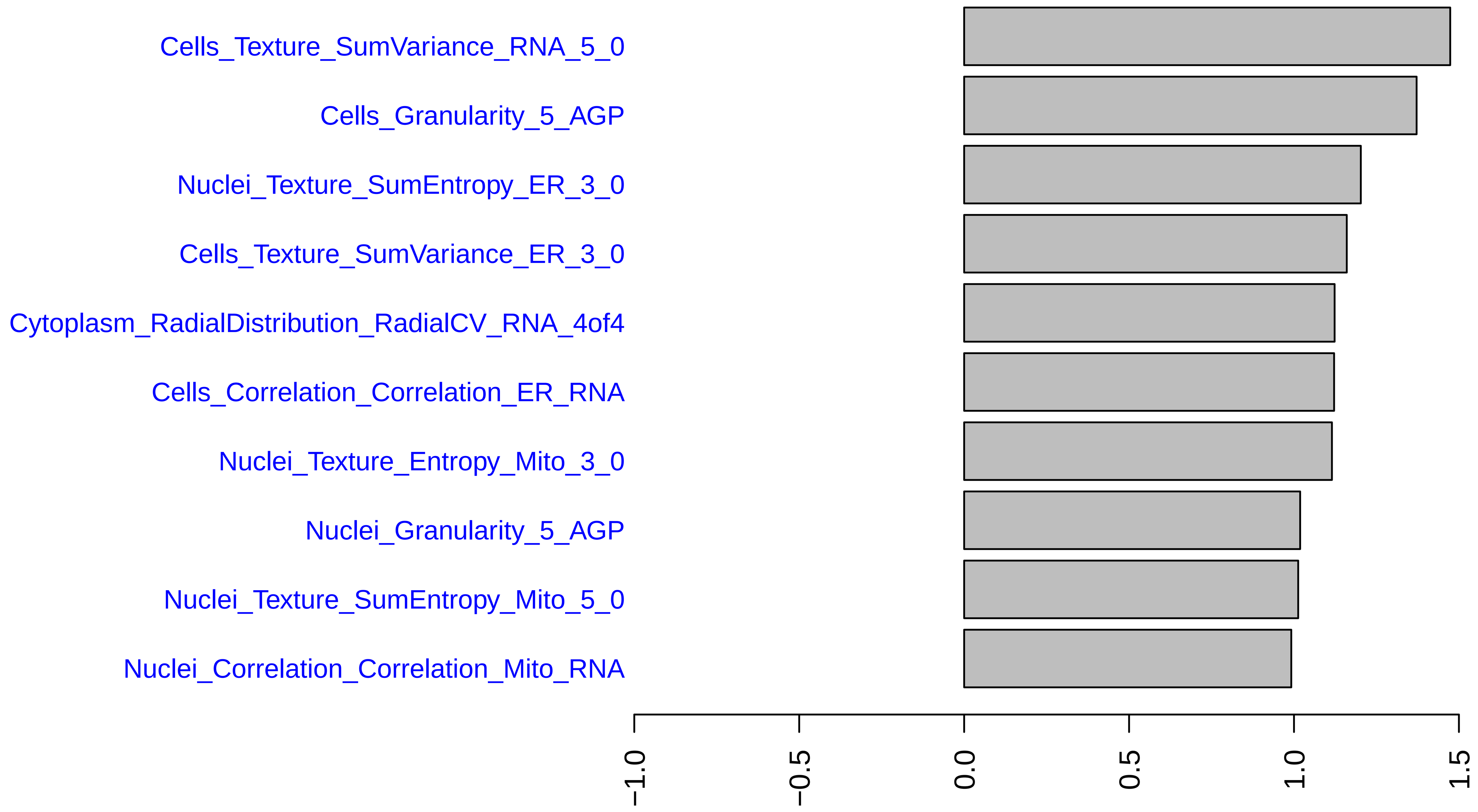


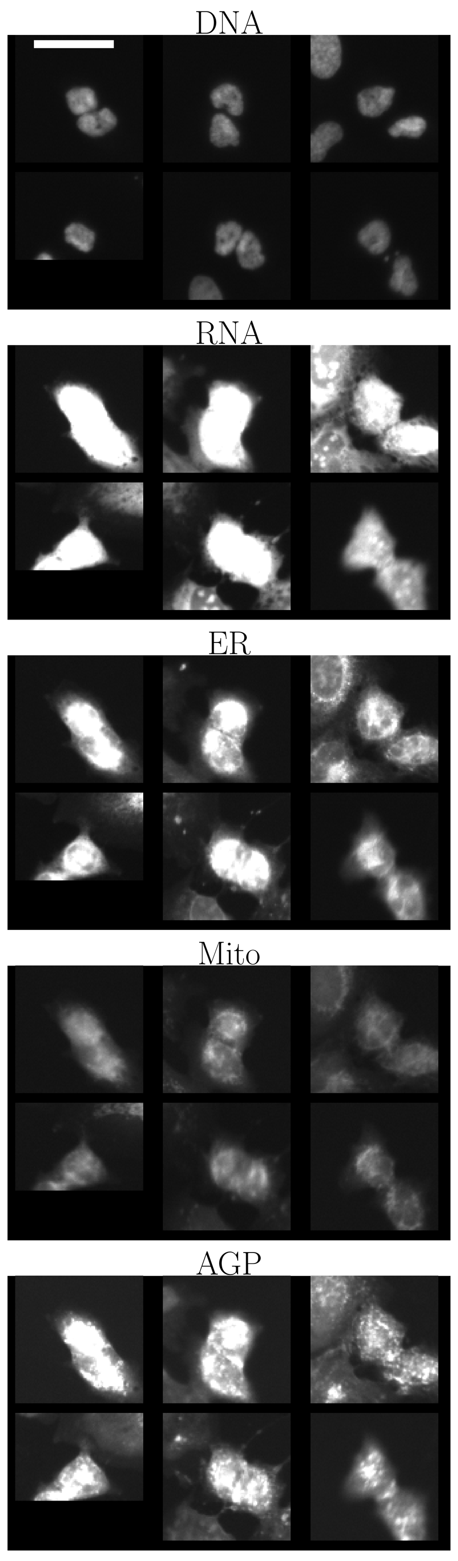


Note : Scale bar is 39.36 $\mu$m.
