# Supplementary material for: Systematic morphological profiling of human gene and allele function via Cell Painting
Source: eLife. 2017 Mar 18;6:e24060. doi: 10.7554/eLife.24060 (PMC5386591; doi:10.7554/eLife.24060)
Supplement: Supplementary file 2. — The details of the contents have been described in Figure 5. DOI: http://dx.doi.org/10.7554/eLife.24060.017 [file elife-24060-supp2.zip › Supplementary file 2/type B/2B.pdf]

Treatment	Expert Annotation	
	Pathway	Regulation Type
CDK4_R24C	Canonical Cell Cycle	Activator
HSP90B1_WT.2	Canonical ER Stress/UPR	Activator
STAT3_C-C	Canonical JAK/STAT	Activator
TBK1_WT.1	Canonical NFkB	Activator

Nuclei_Intensity_IntegratedIntensity_DNA

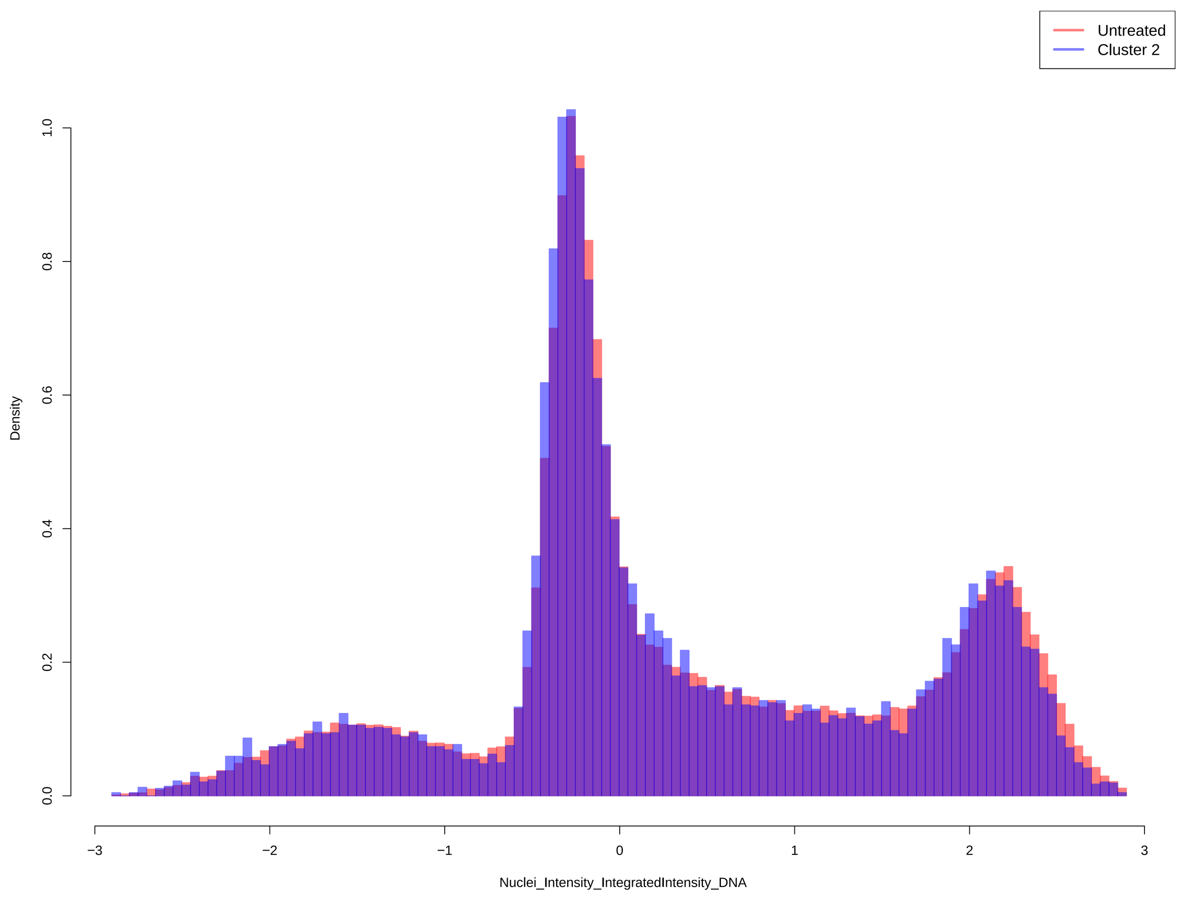

Different categories of cells in the cluster :

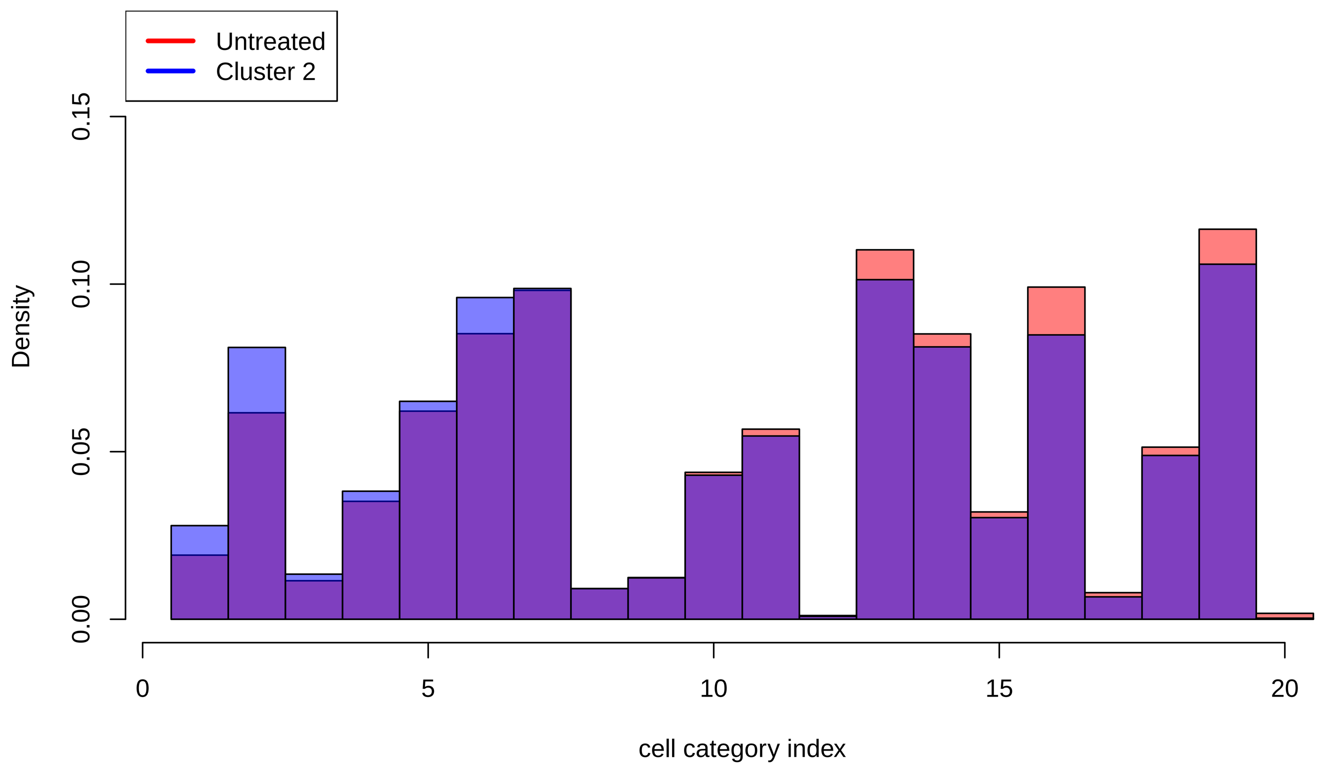

Different categories of cells in single genes in the cluster :

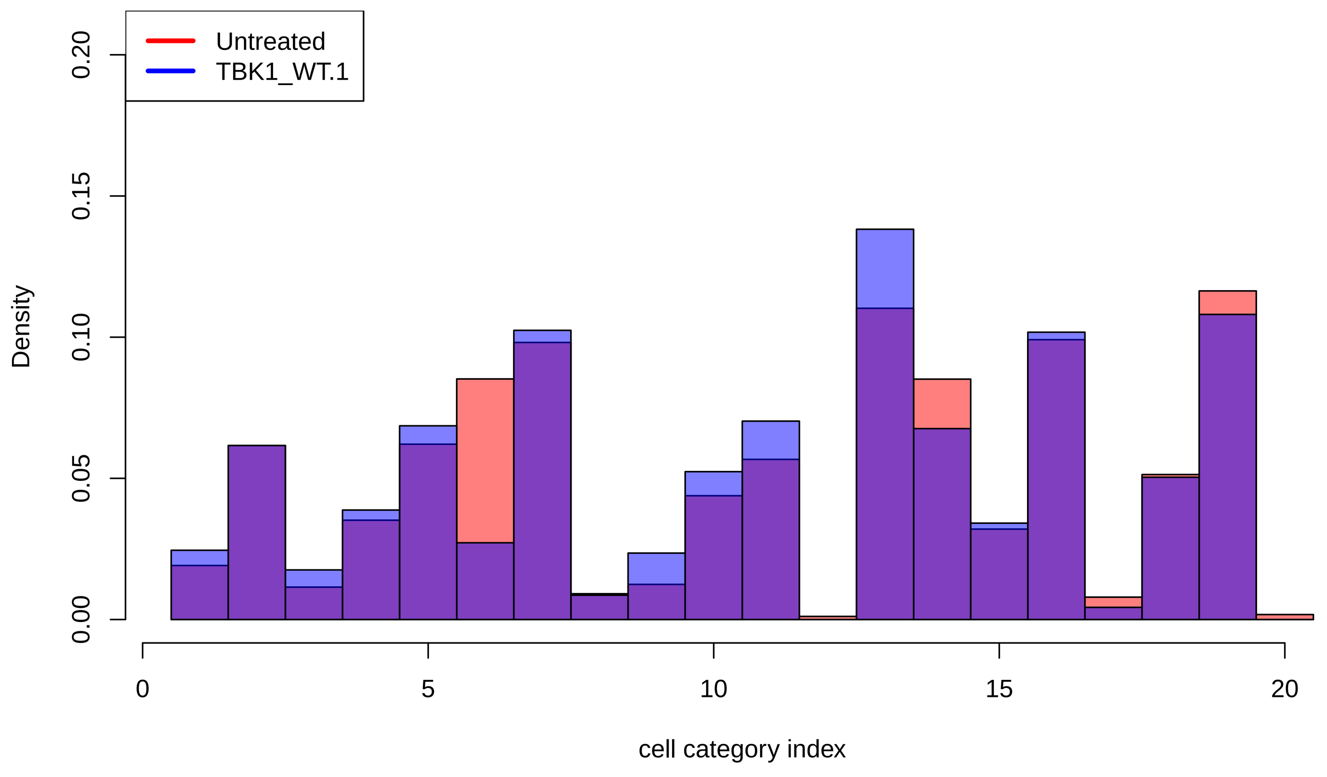

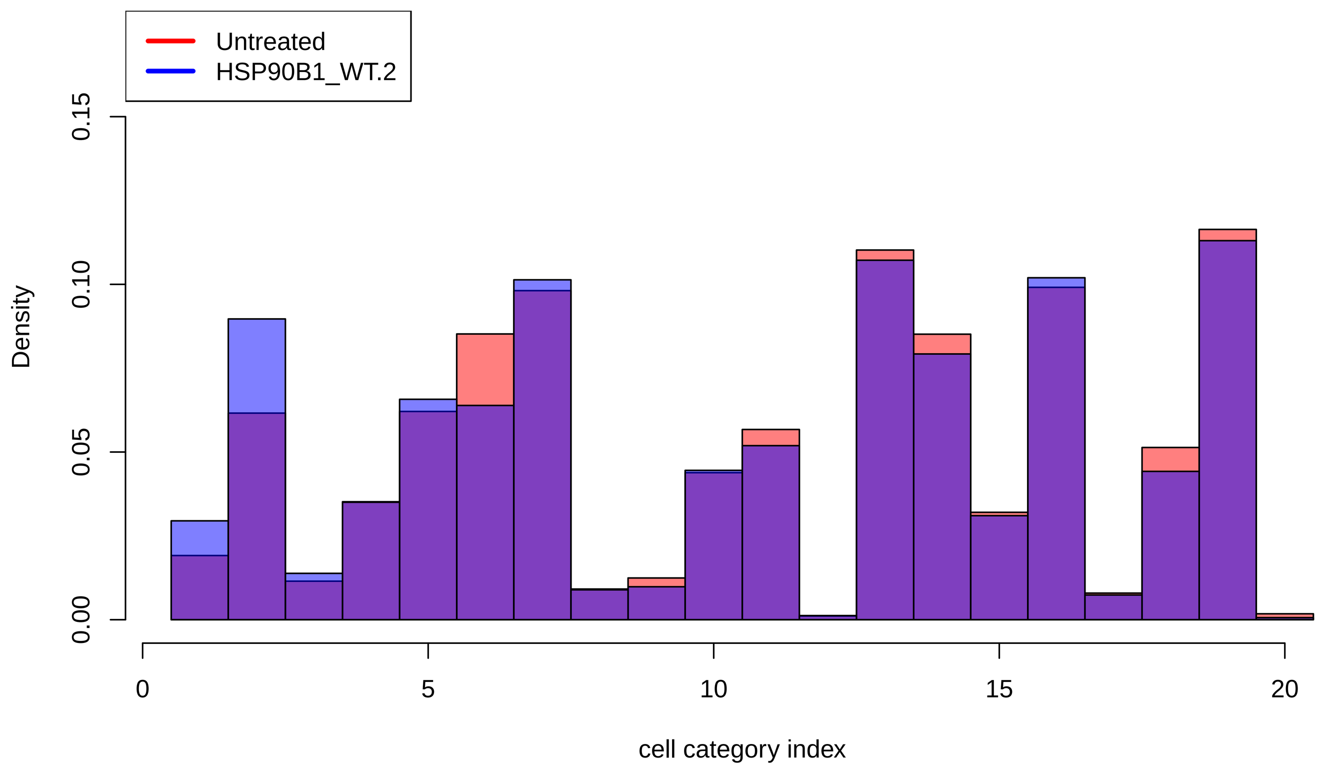

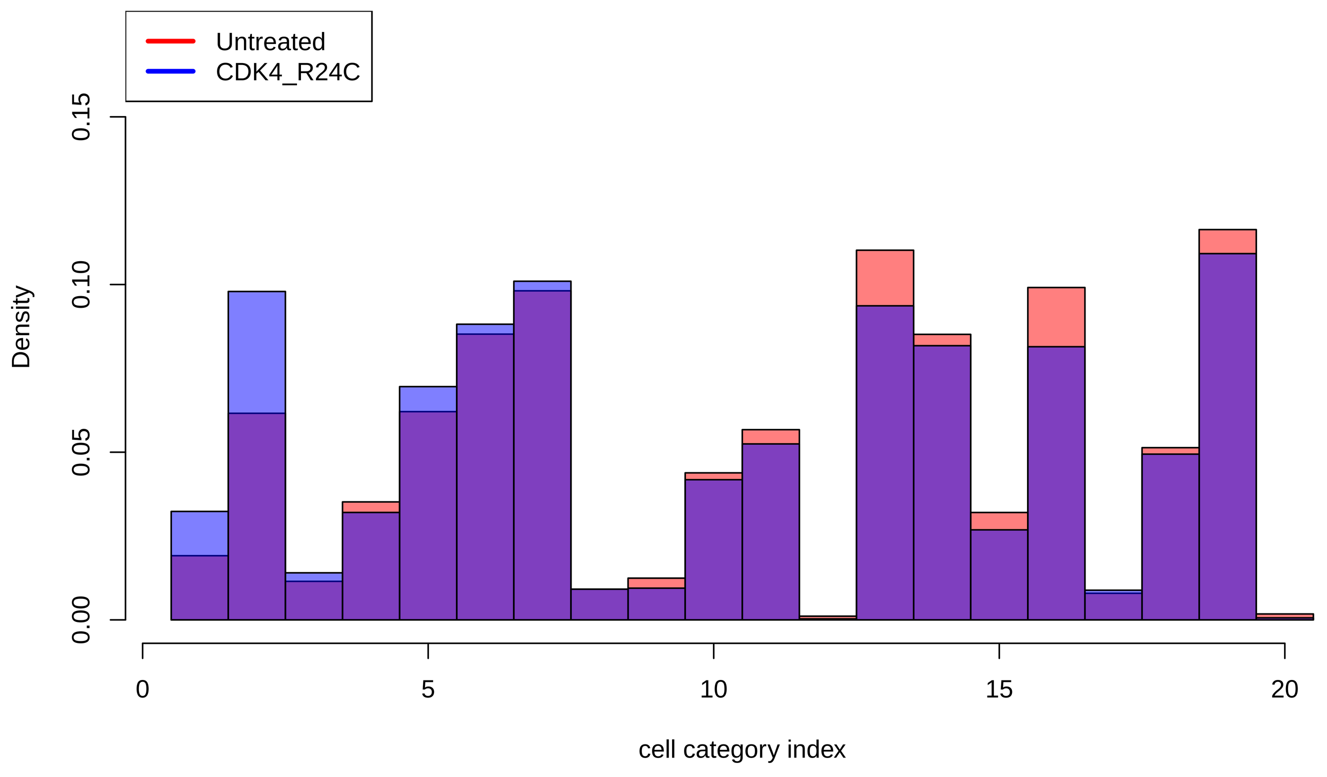

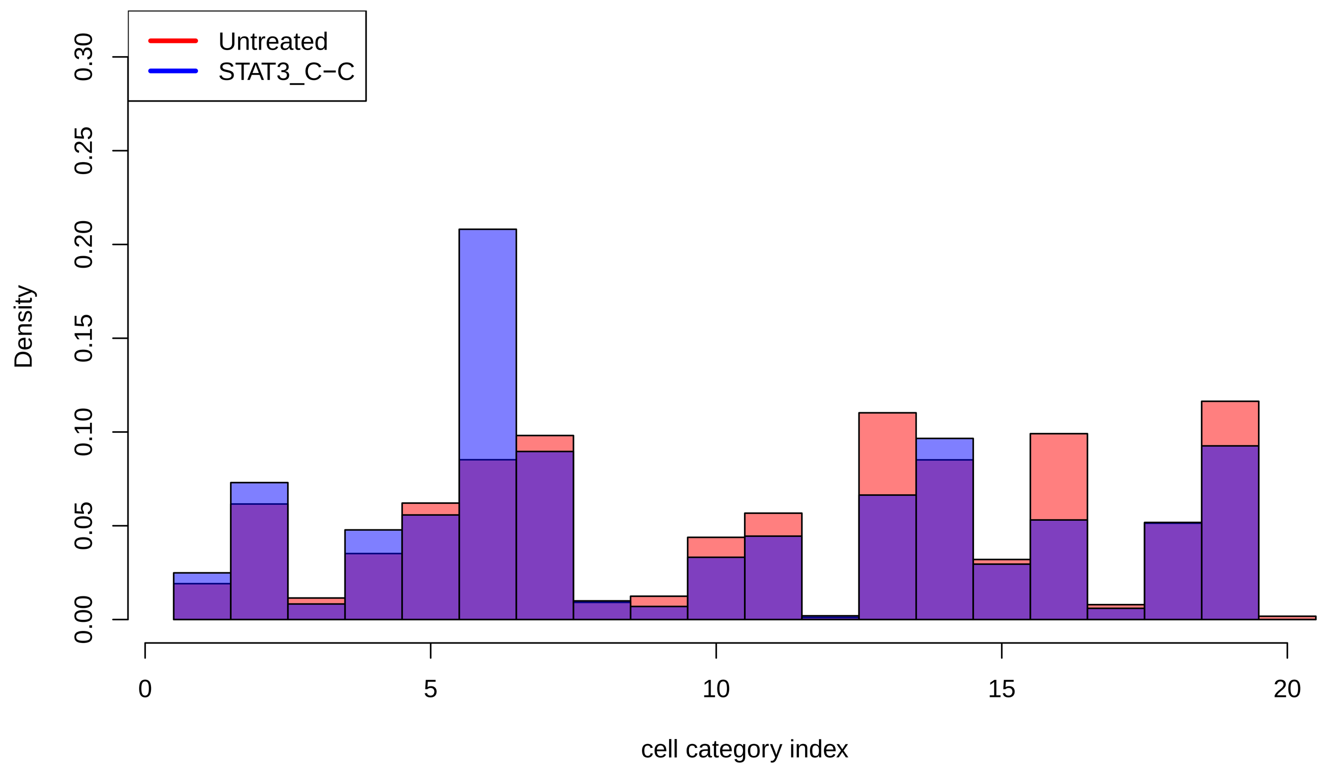
Untreated
STAT3_C−C
Density
0.00
0.05
0.10
0.15
0.20
0.25
0.30
0
5
10
15
20
cell category index

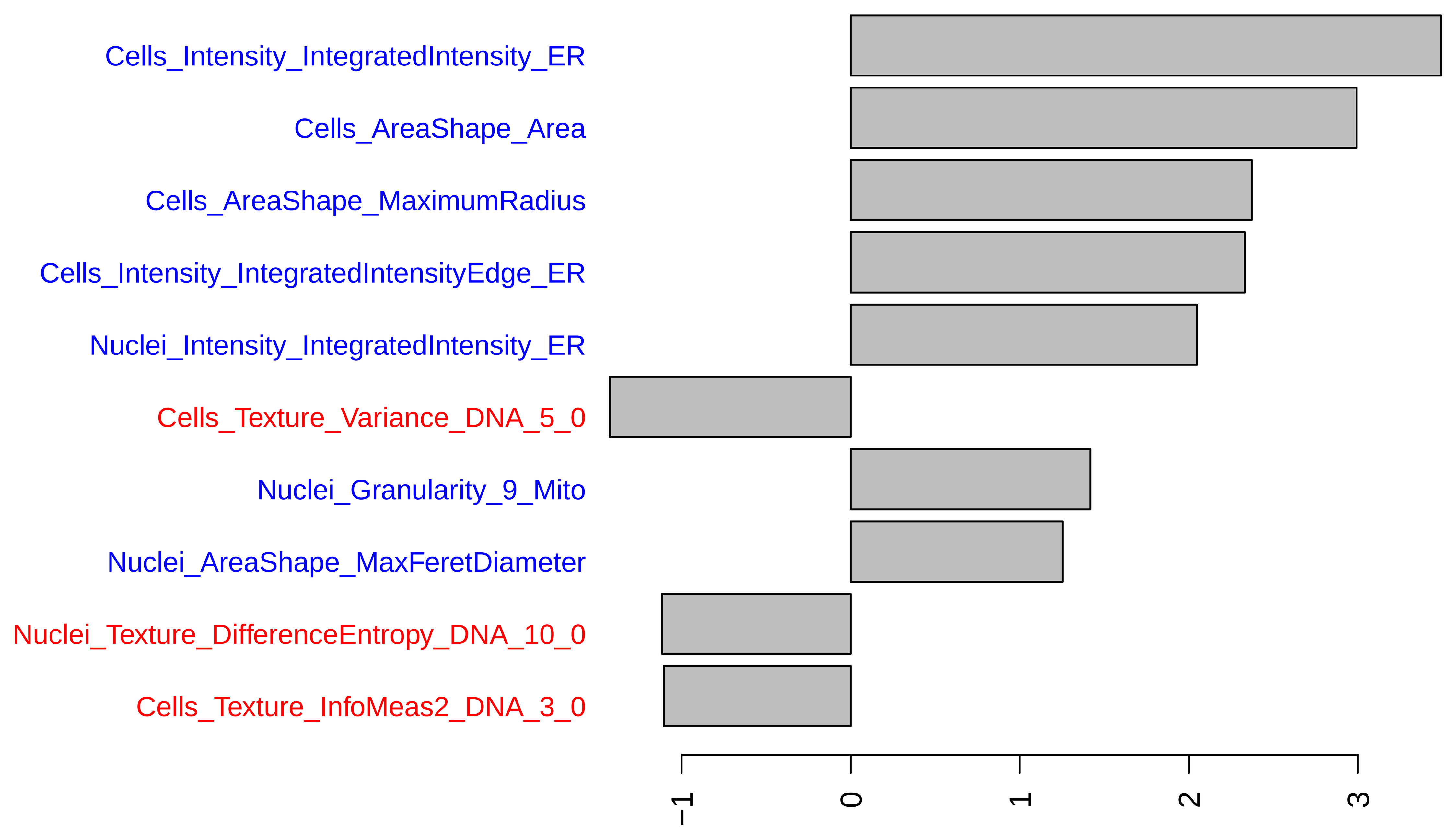

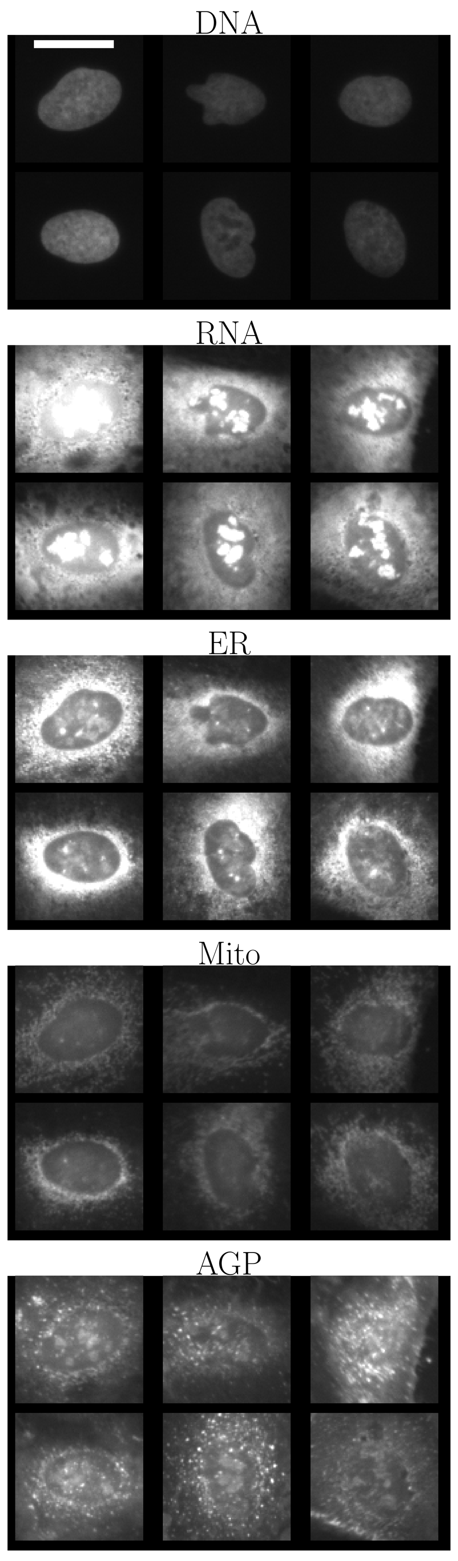

Note : Scale bar is 39.36 μm.

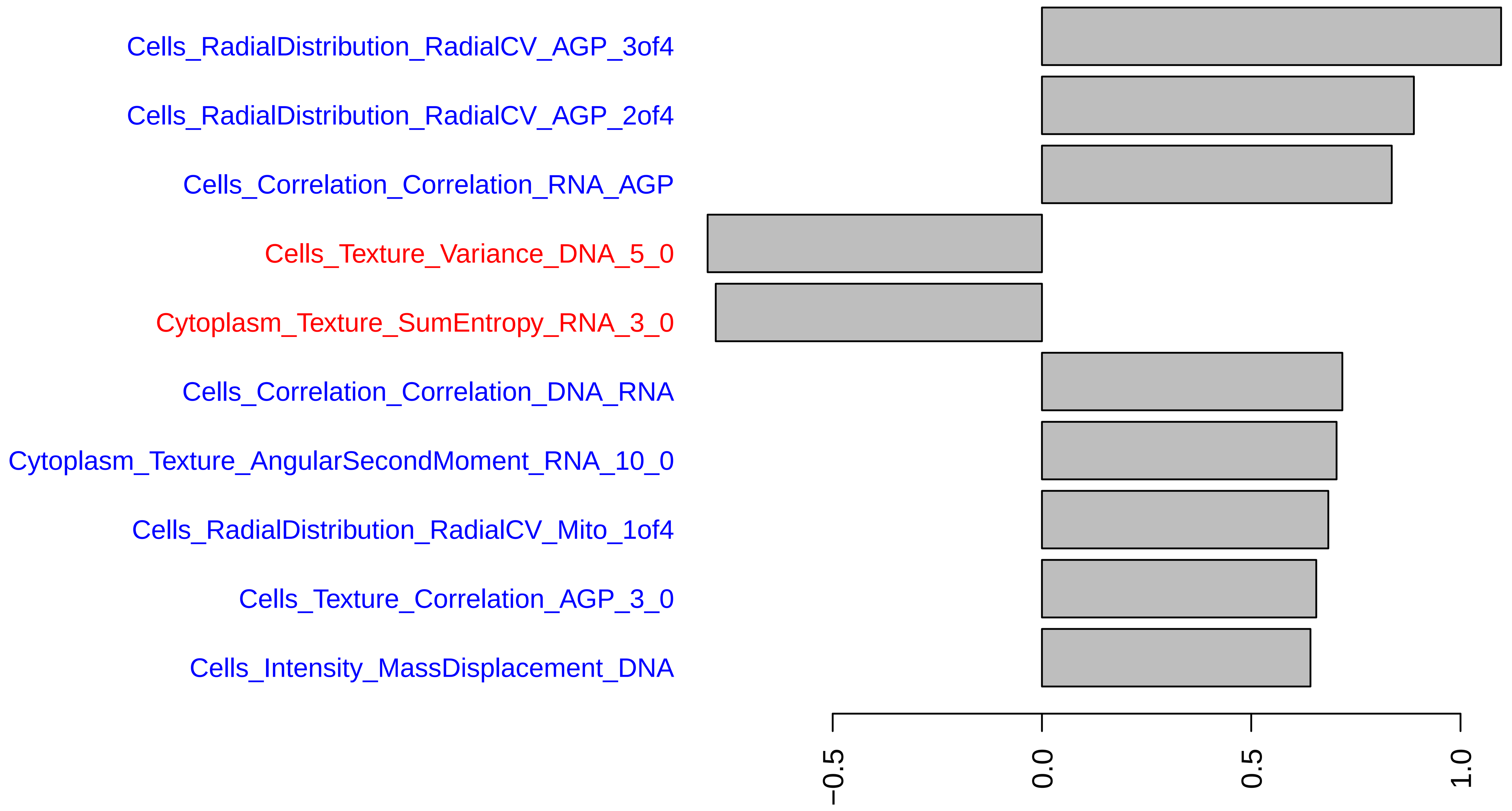

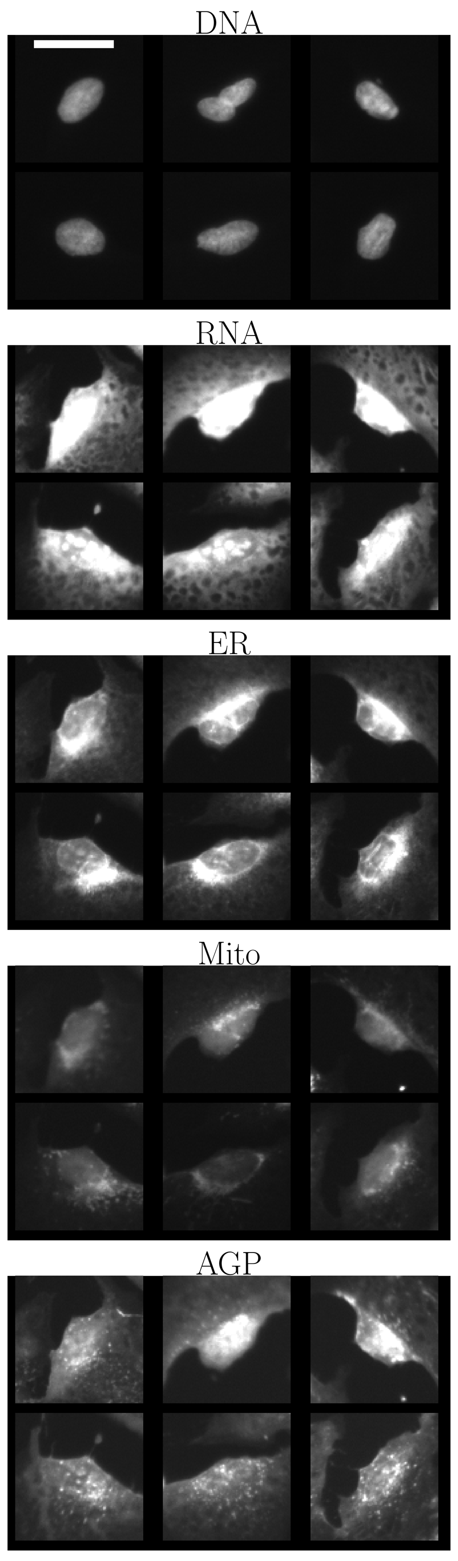

Note : Scale bar is 39.36 μm.

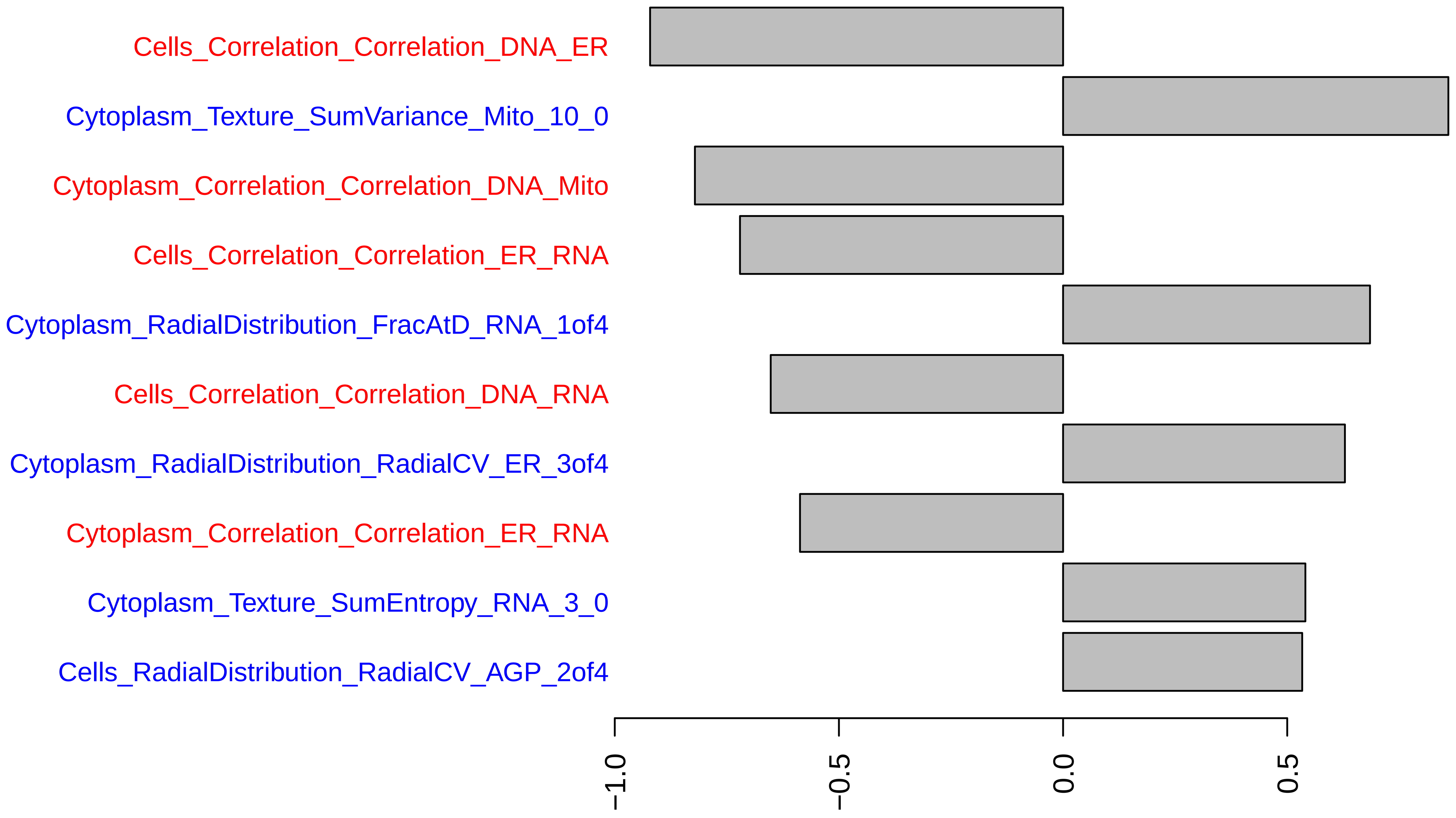

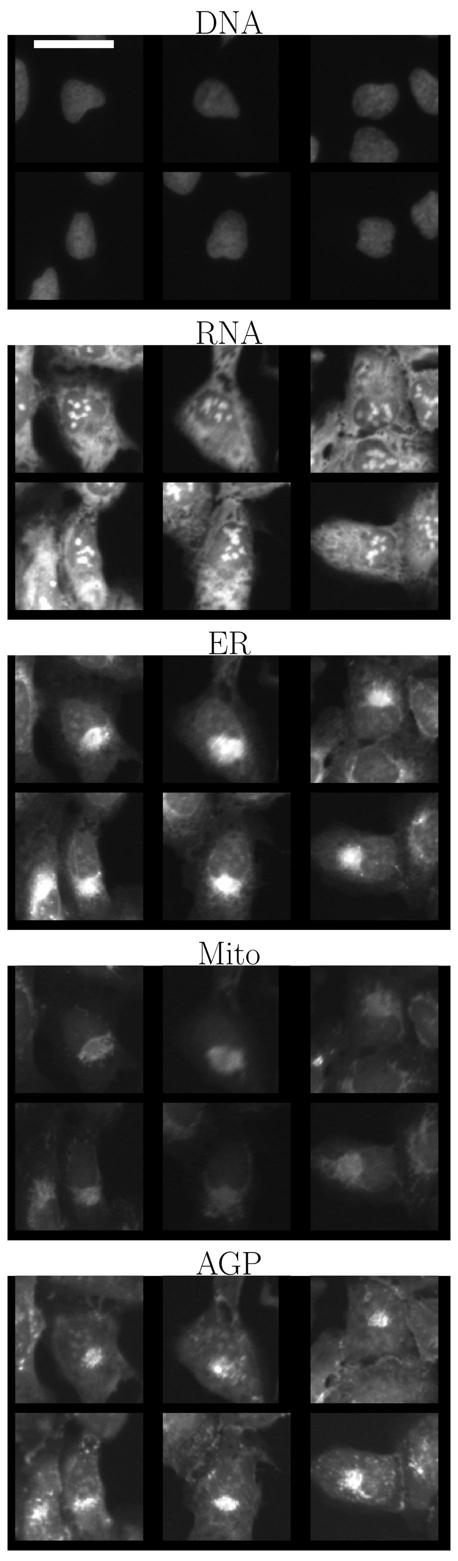

Note : Scale bar is 39.36 μm.
