# Supplementary material for: Systematic morphological profiling of human gene and allele function via Cell Painting
Source: eLife. 2017 Mar 18;6:e24060. doi: 10.7554/eLife.24060 (PMC5386591; doi:10.7554/eLife.24060)
Supplement: Supplementary file 2. — The details of the contents have been described in Figure 5. DOI: http://dx.doi.org/10.7554/eLife.24060.017 [file elife-24060-supp2.zip › Supplementary file 2/type B/5B.pdf]

	Expert Annotation	
Treatment	Pathway	Regulation Type
MAPK14_WT.1	Canonical MAPK	Activator
MAPK14_WT.2	Canonical MAPK	Activator
RBPJ_WT.1	NOTCH	Activator
RBPJ_WT.2	NOTCH	Activator
XBP1_WT.1	Canonical ER Stress/UPR	Activator
XBP1_WT.2	Canonical ER Stress/UPR	Activator

Nuclei_Intensity_IntegratedIntensity_DNA

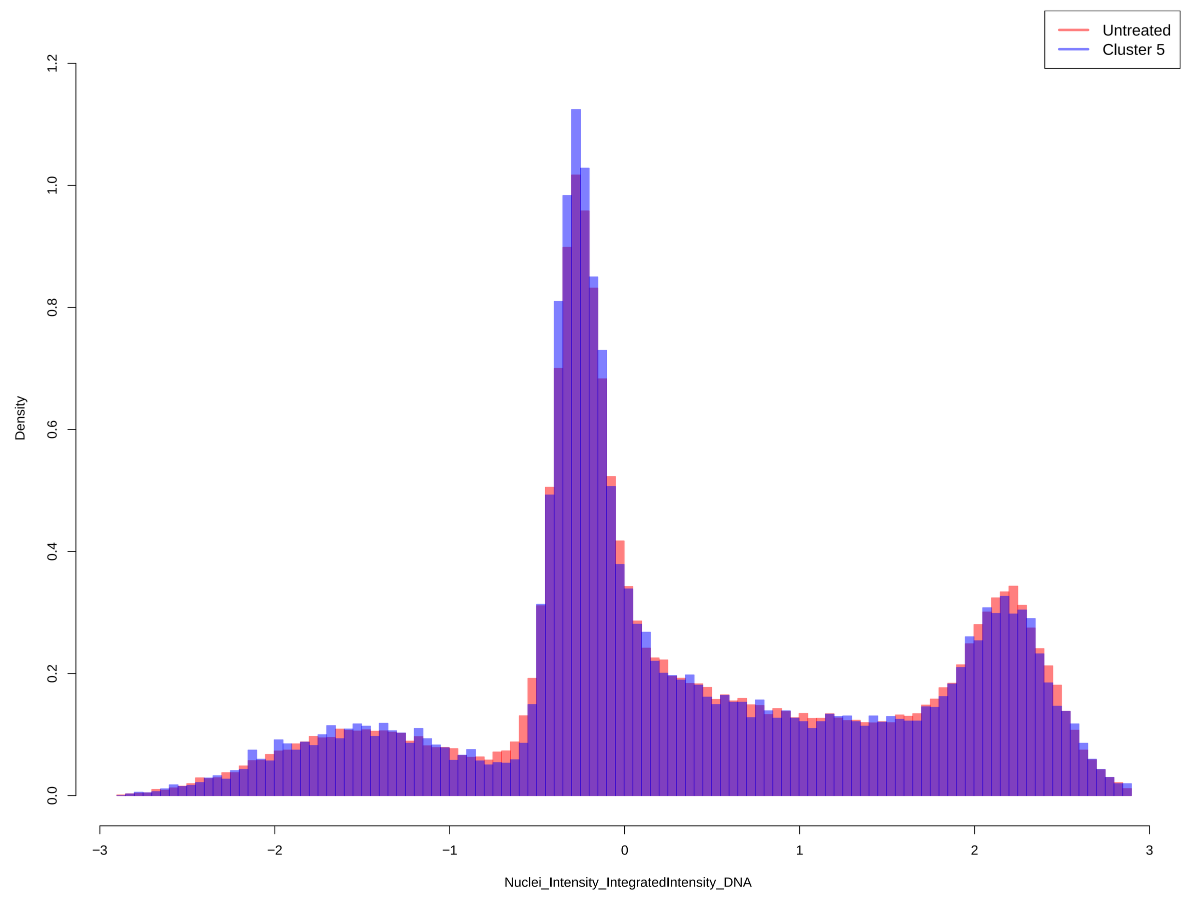

Different categories of cells in the cluster :

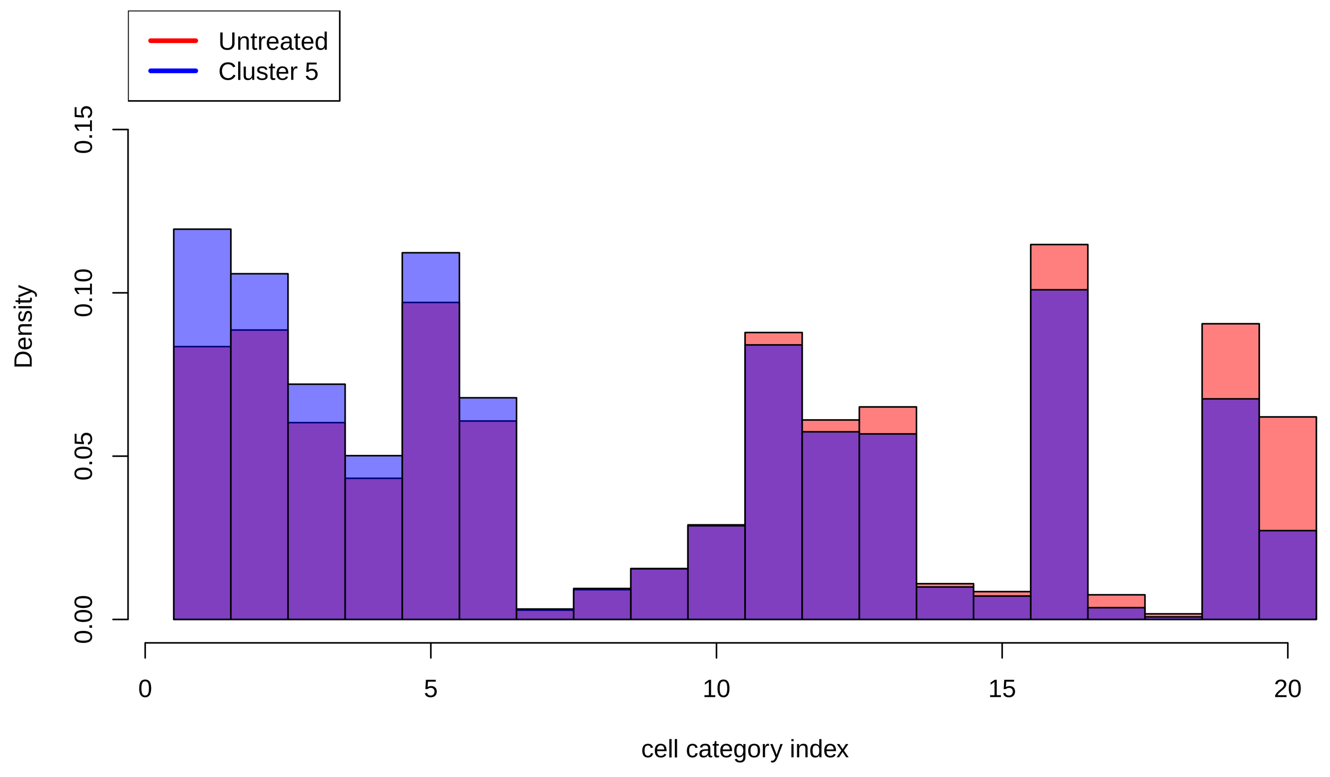

Different categories of cells in single genes in the cluster :

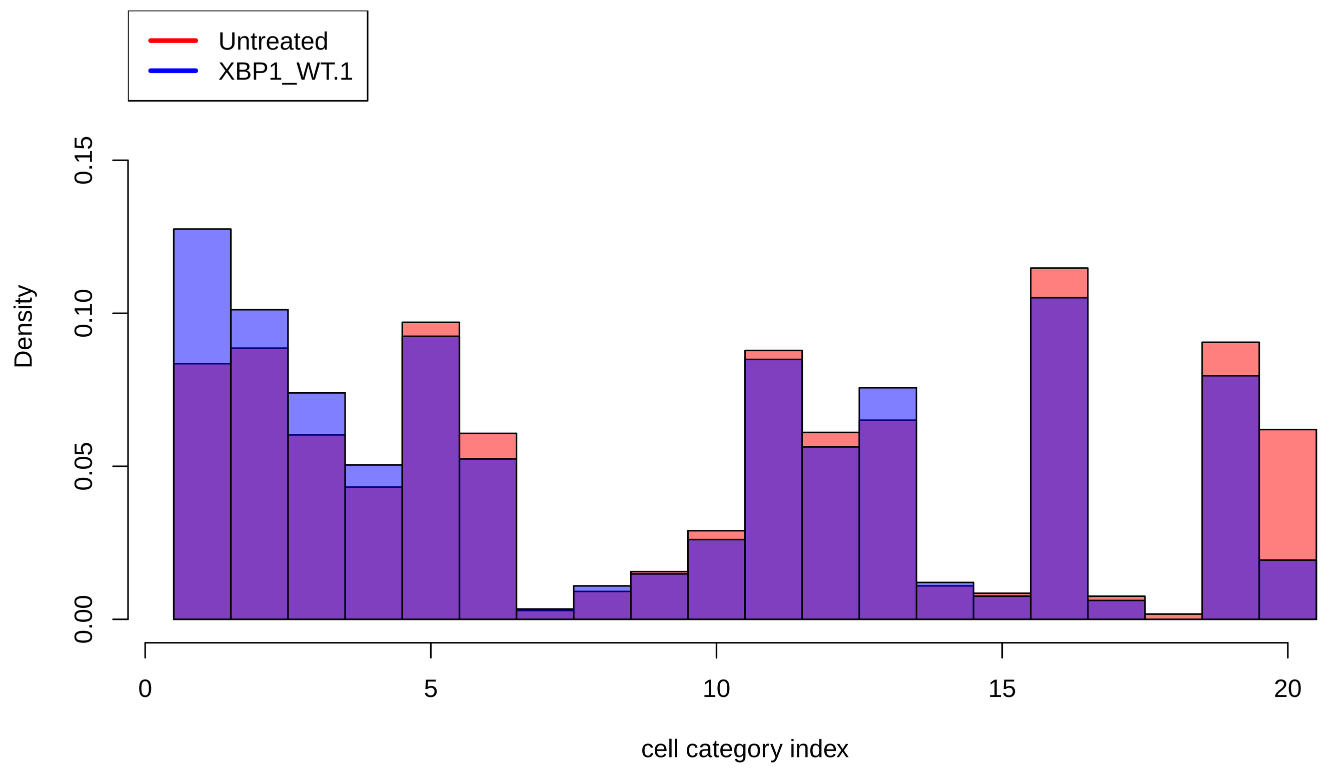

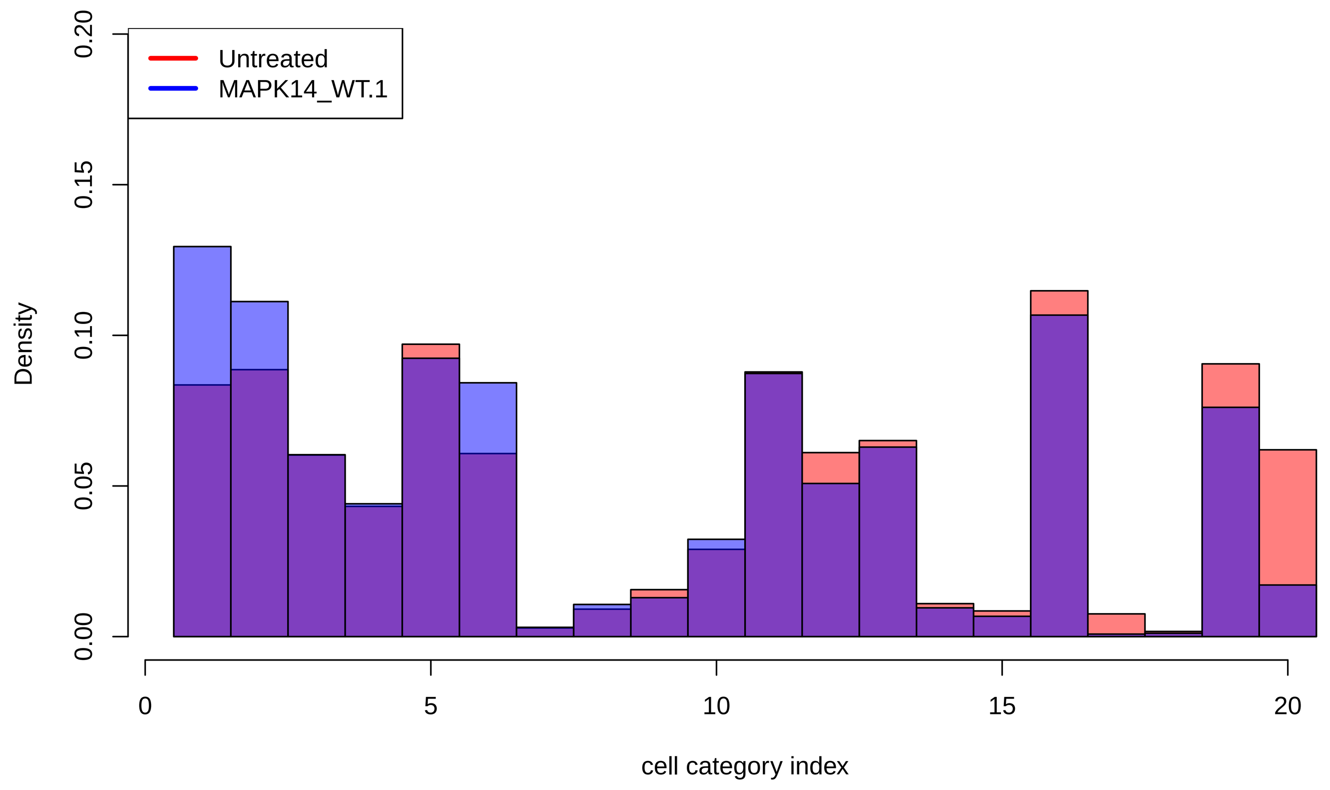

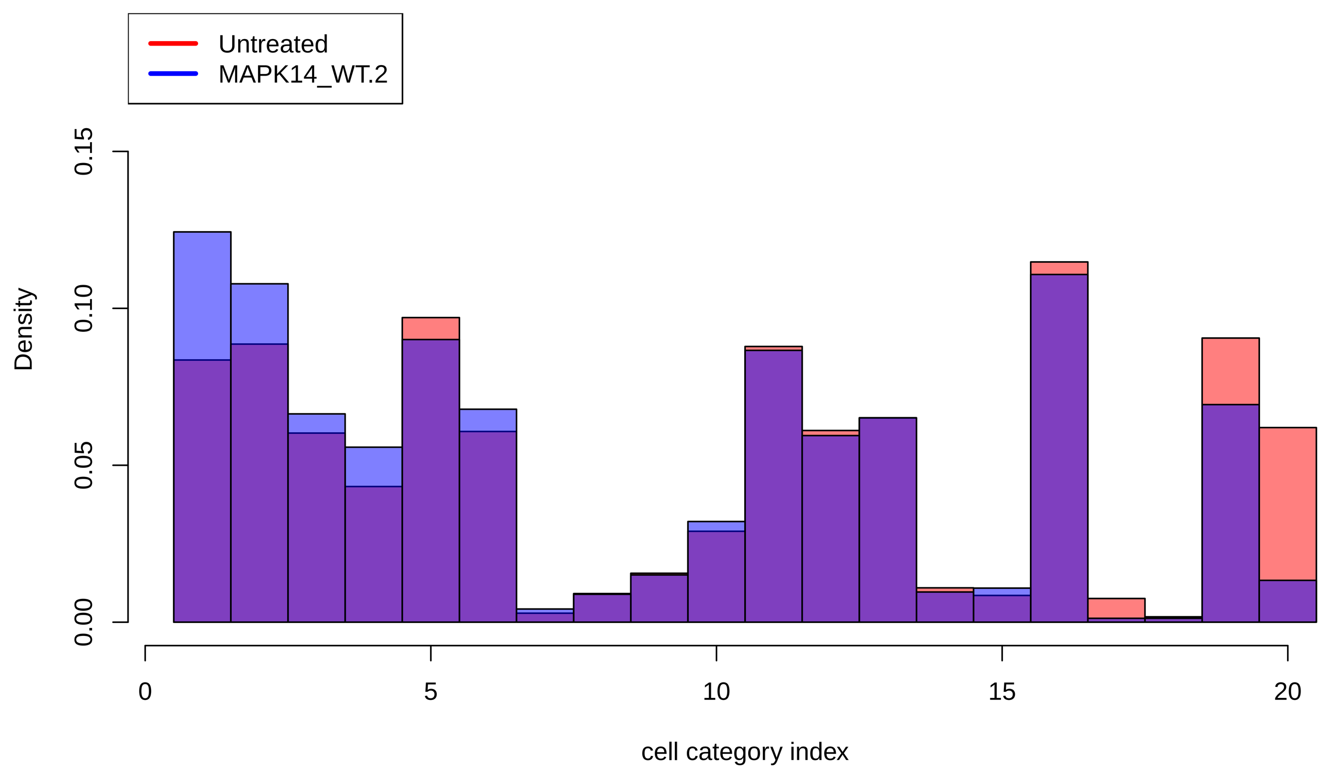

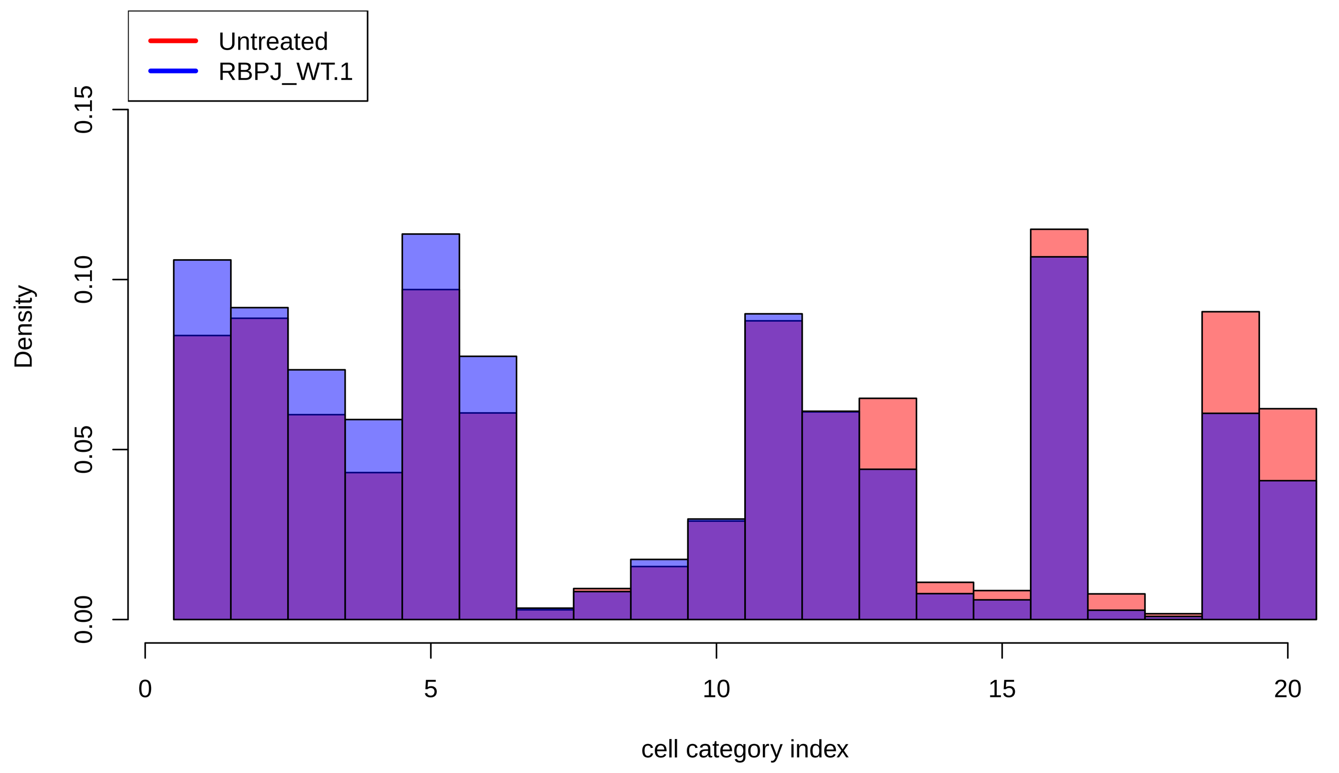
Untreated
RBPJ_WT.1
Density
0.00
0.05
0.10
0.15
0
5
10
15
20
cell category index

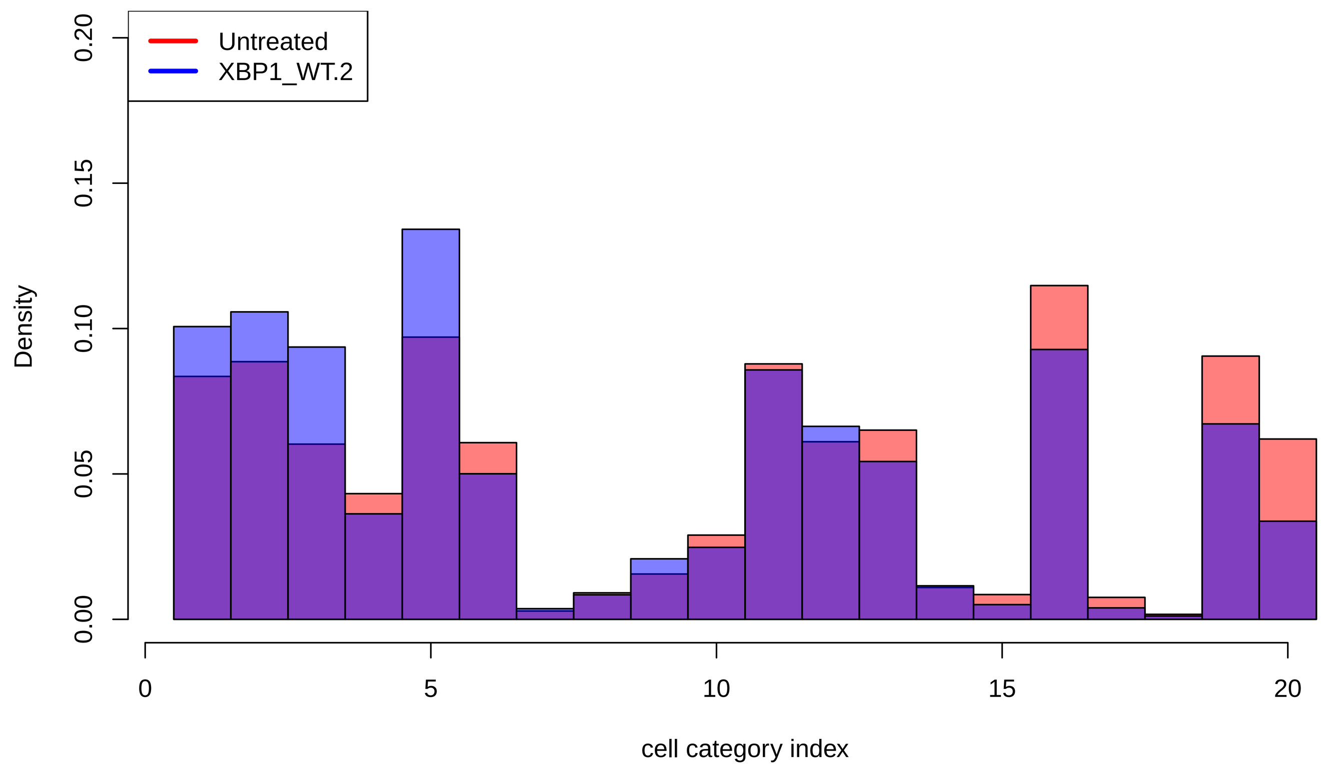
Untreated
XBP1_WT.2
Density
0.00
0.05
0.10
0.15
0.20
0
5
10
15
20
cell category index

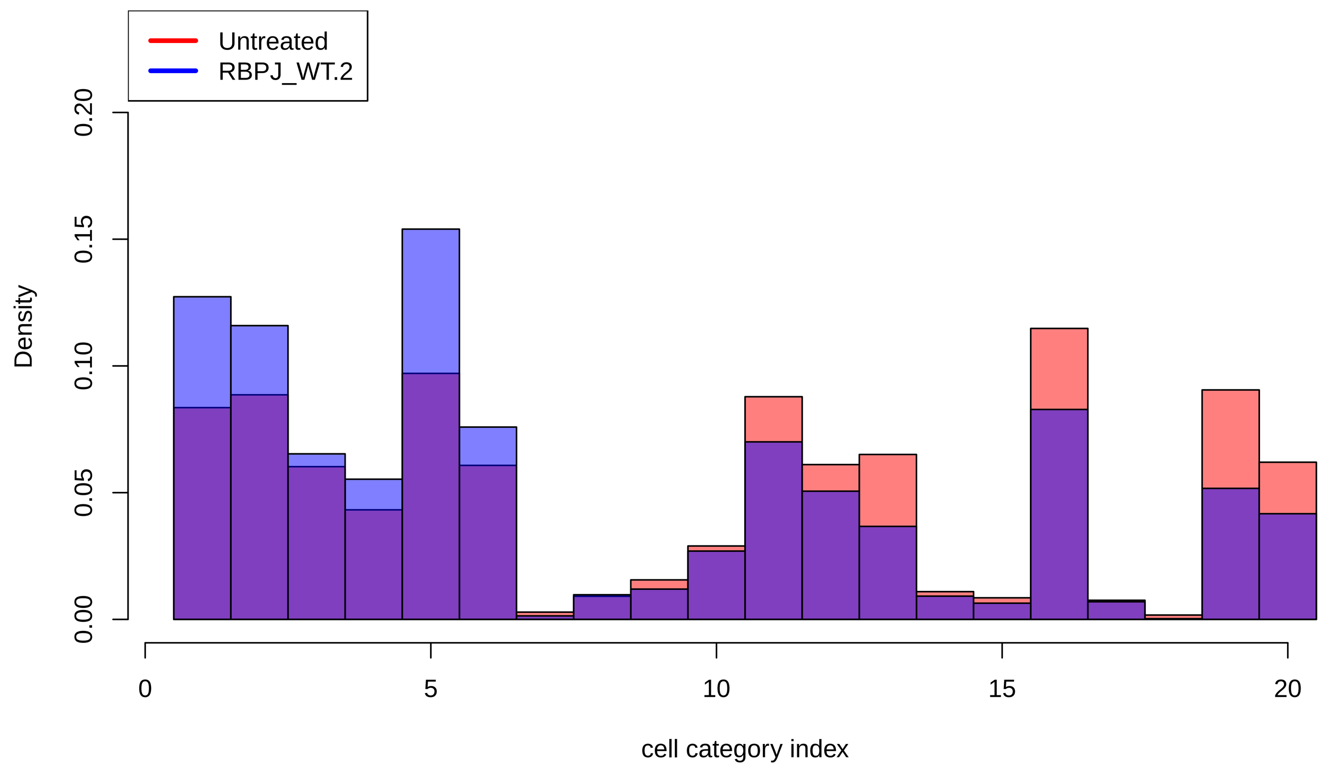
Untreated
RBPJ_WT.2
Density
0.00
0.05
0.10
0.15
0.20
0
5
10
15
20
cell category index

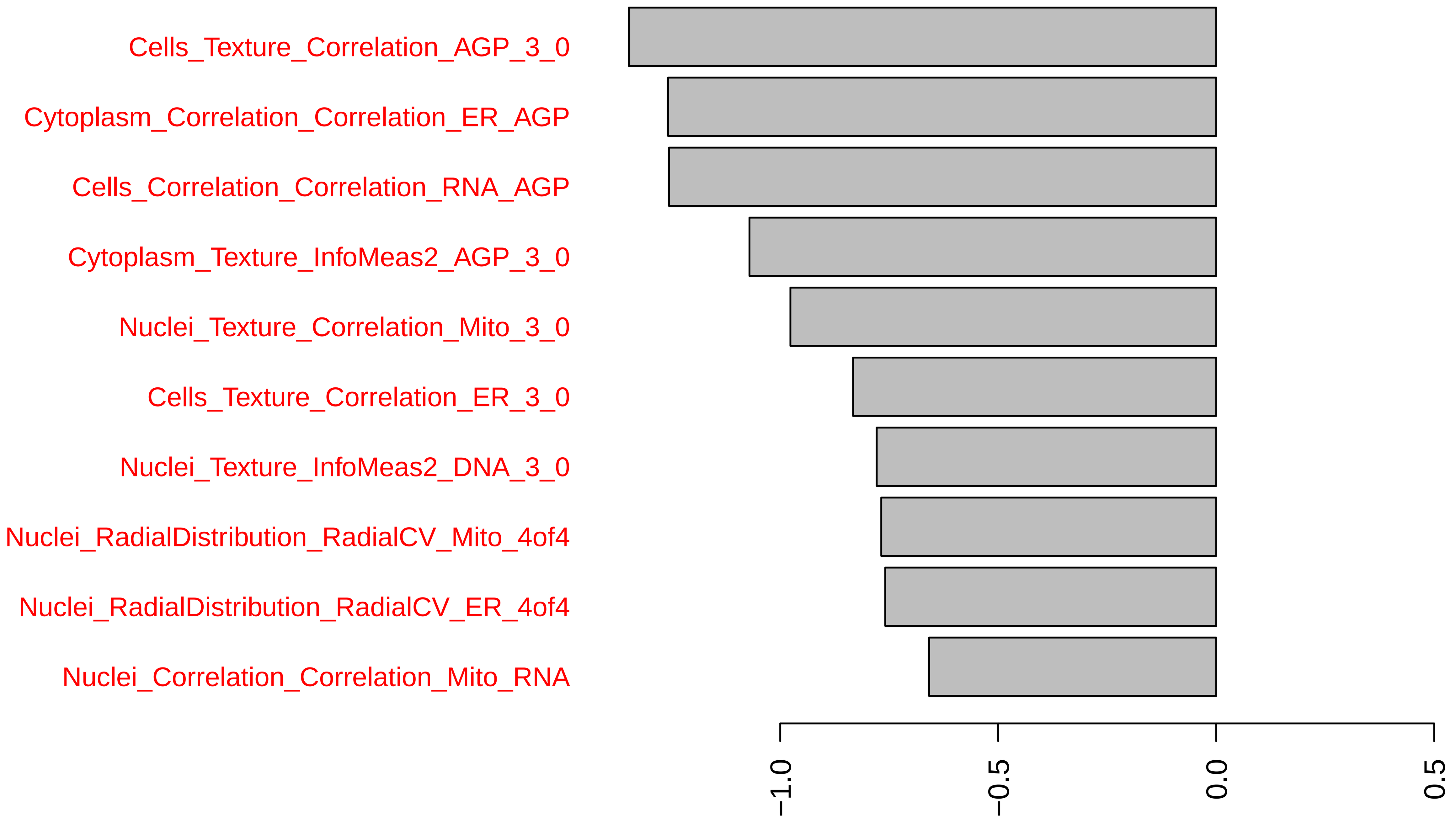

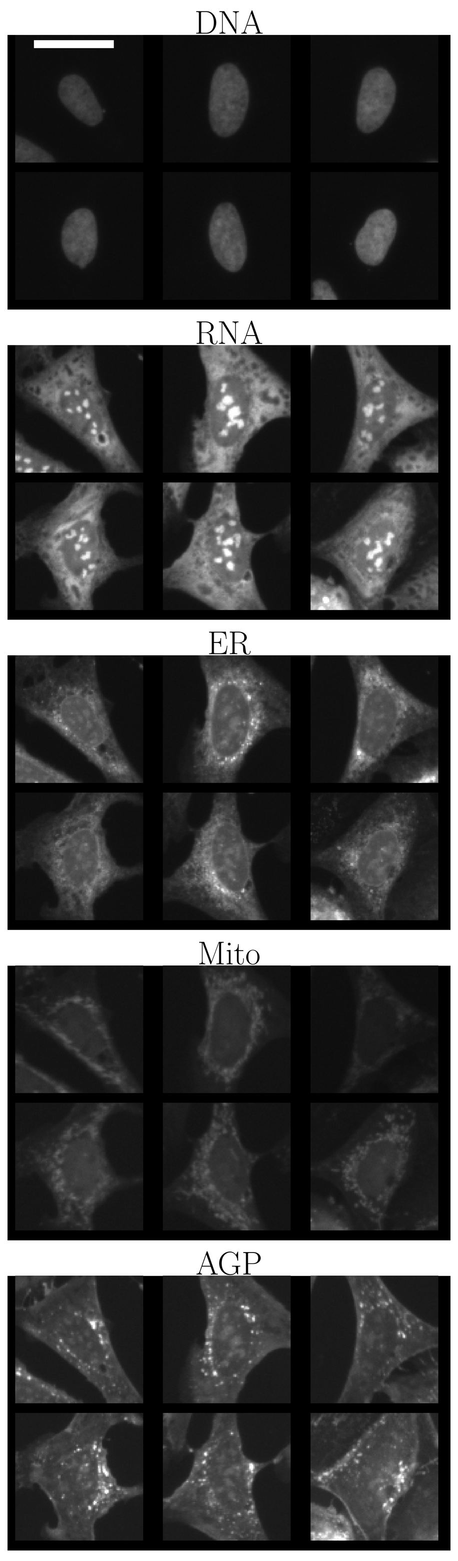

Note : Scale bar is 39.36 μm.

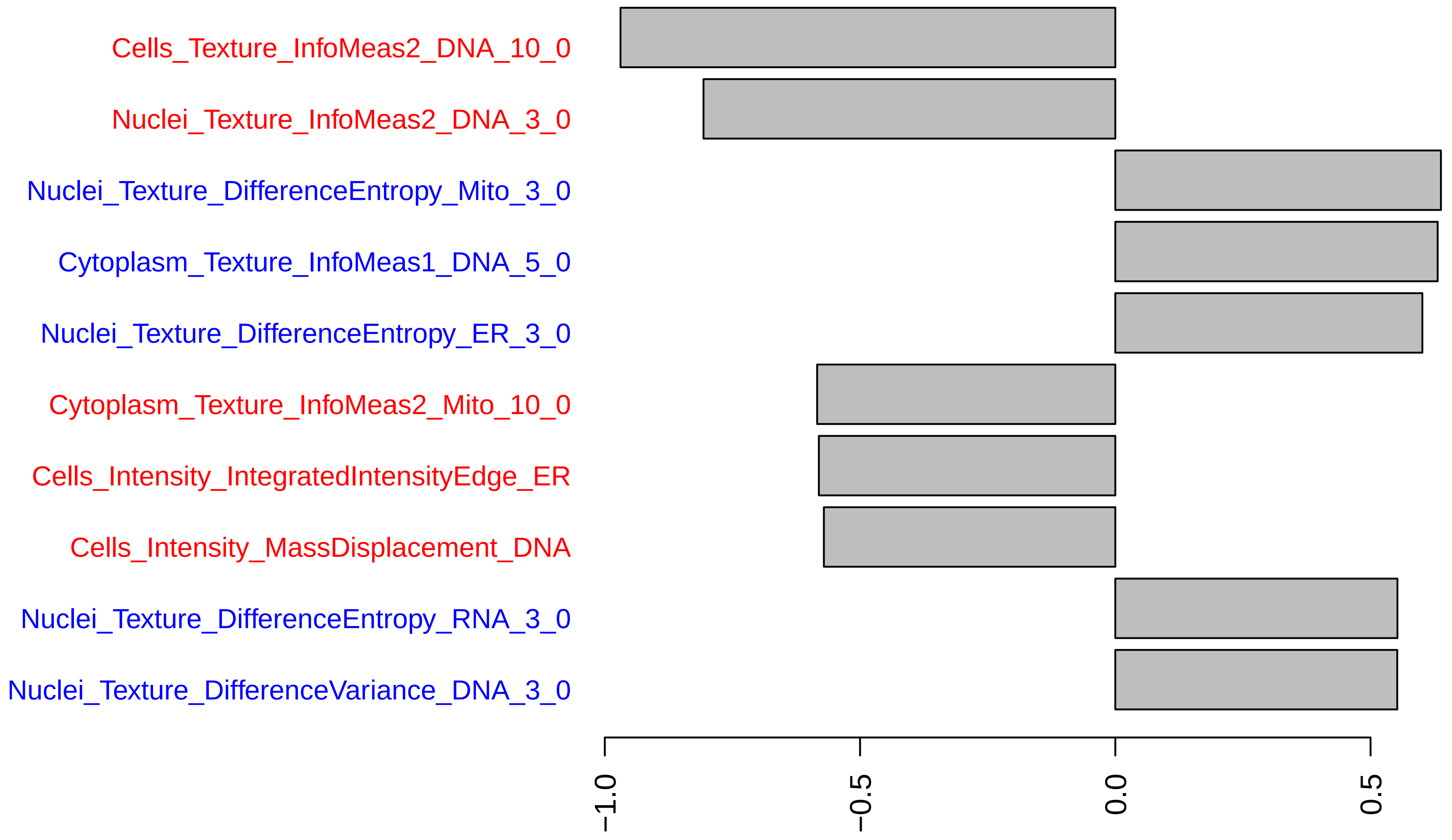

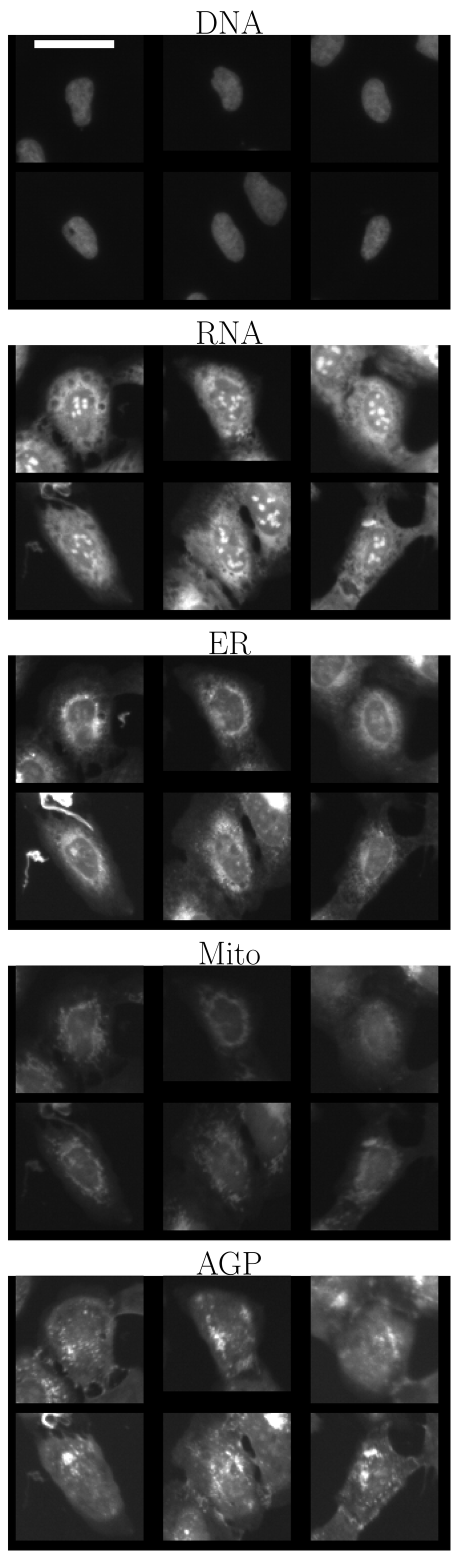

Note : Scale bar is 39.36 μm.

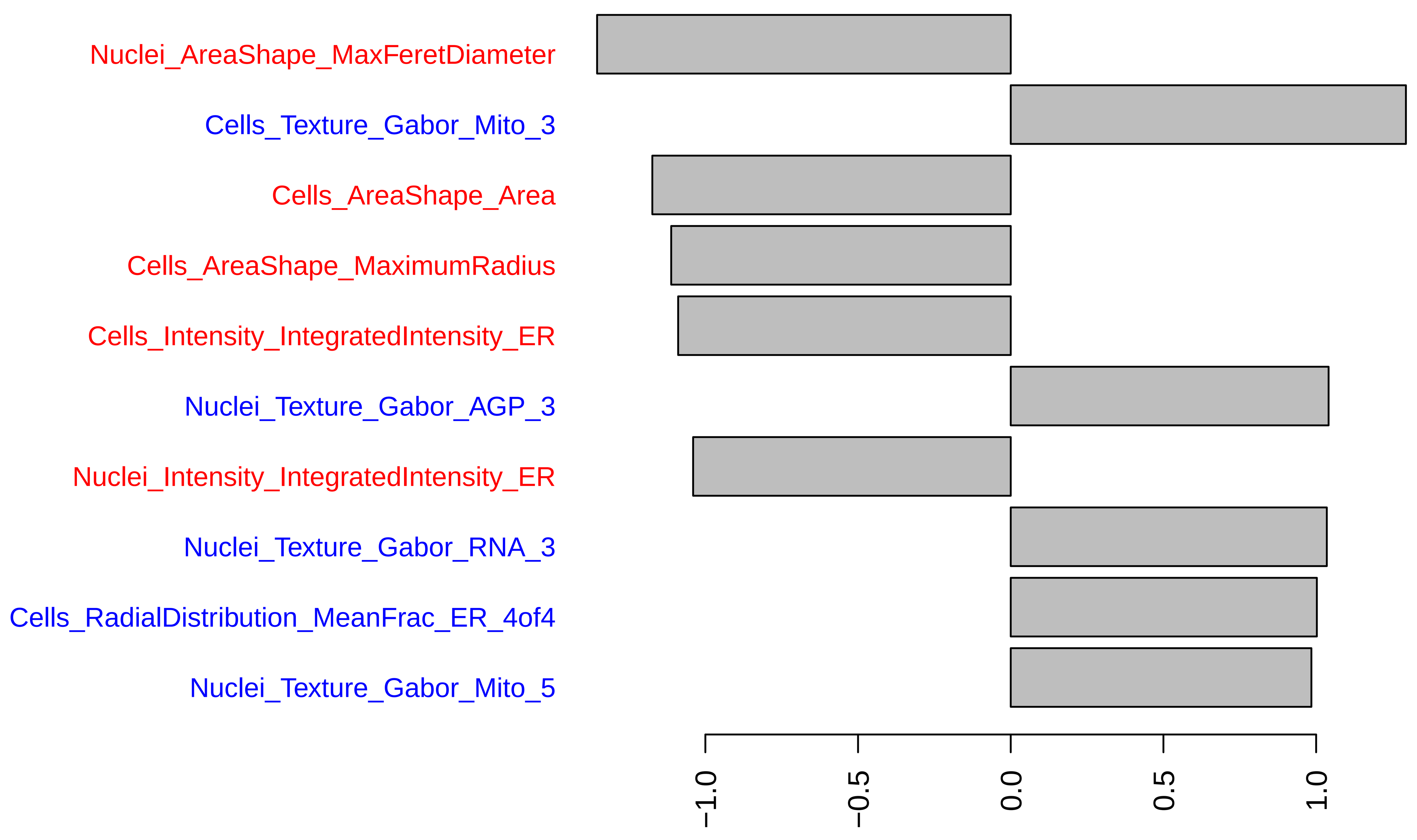

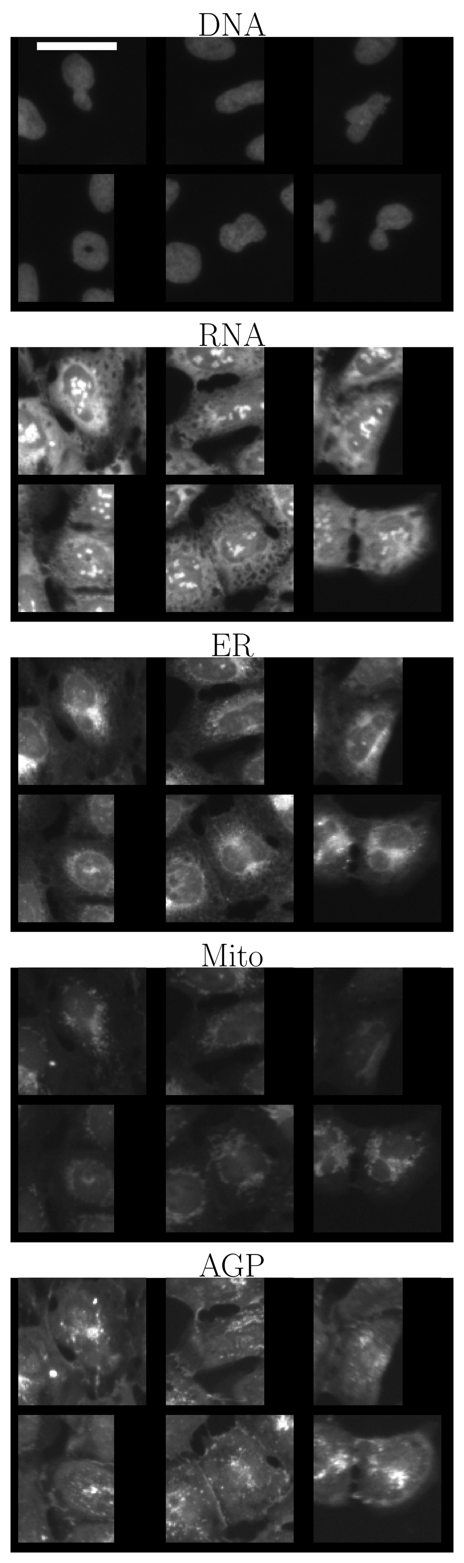

Note : Scale bar is 39.36 μm.

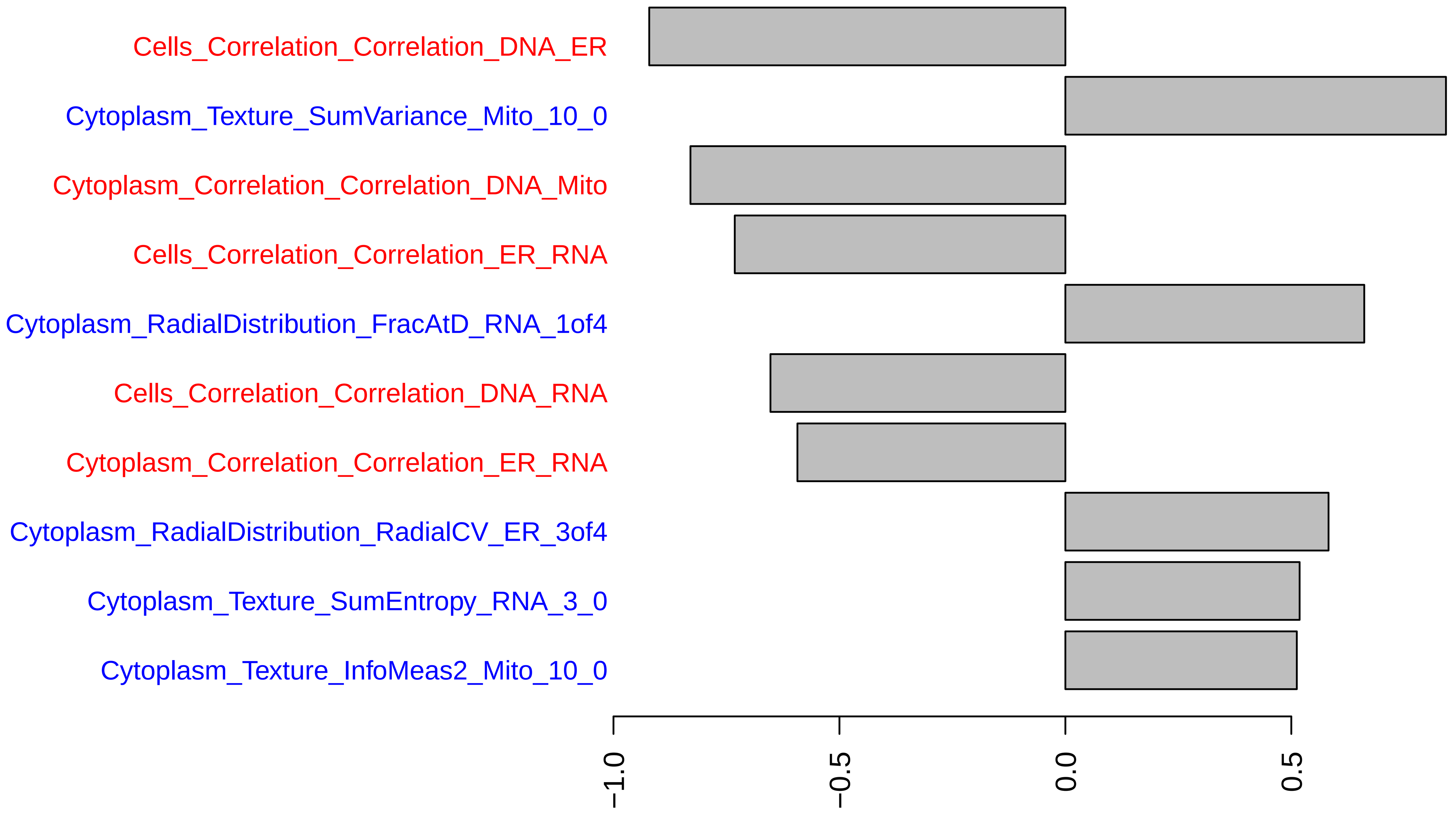

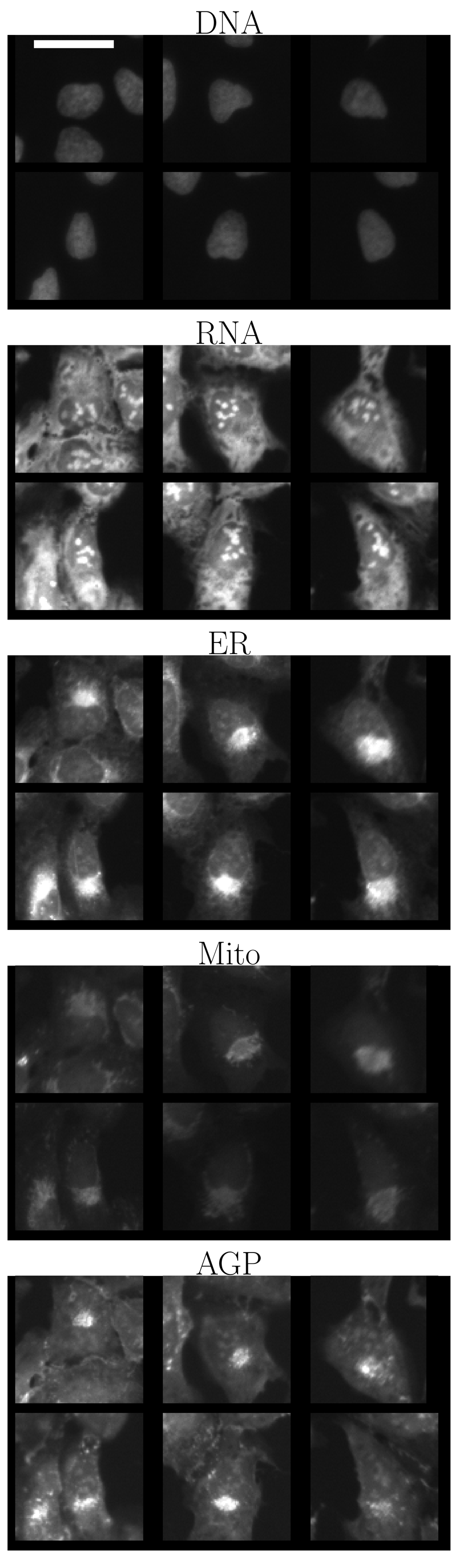

Note : Scale bar is 39.36 μm.

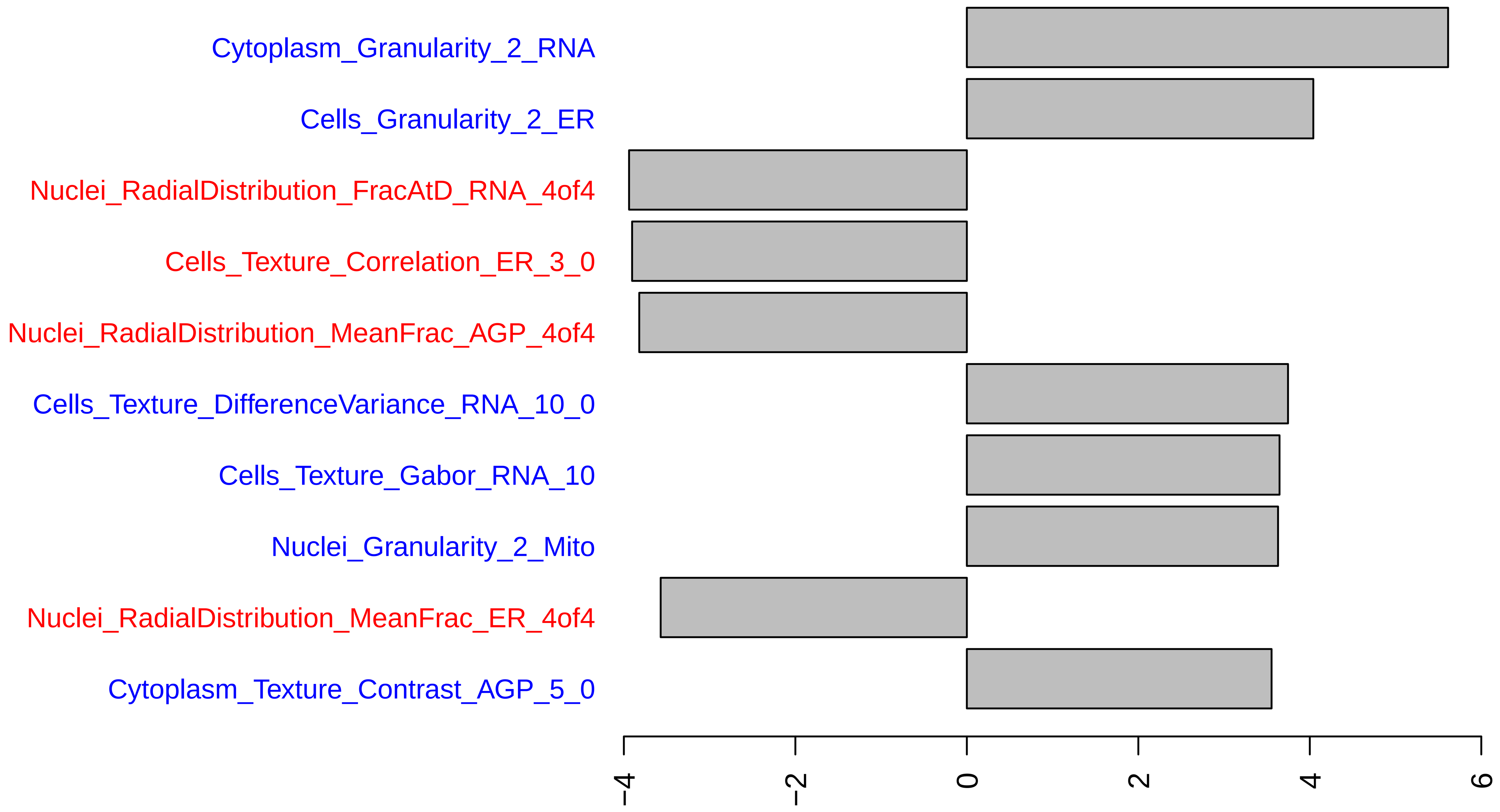

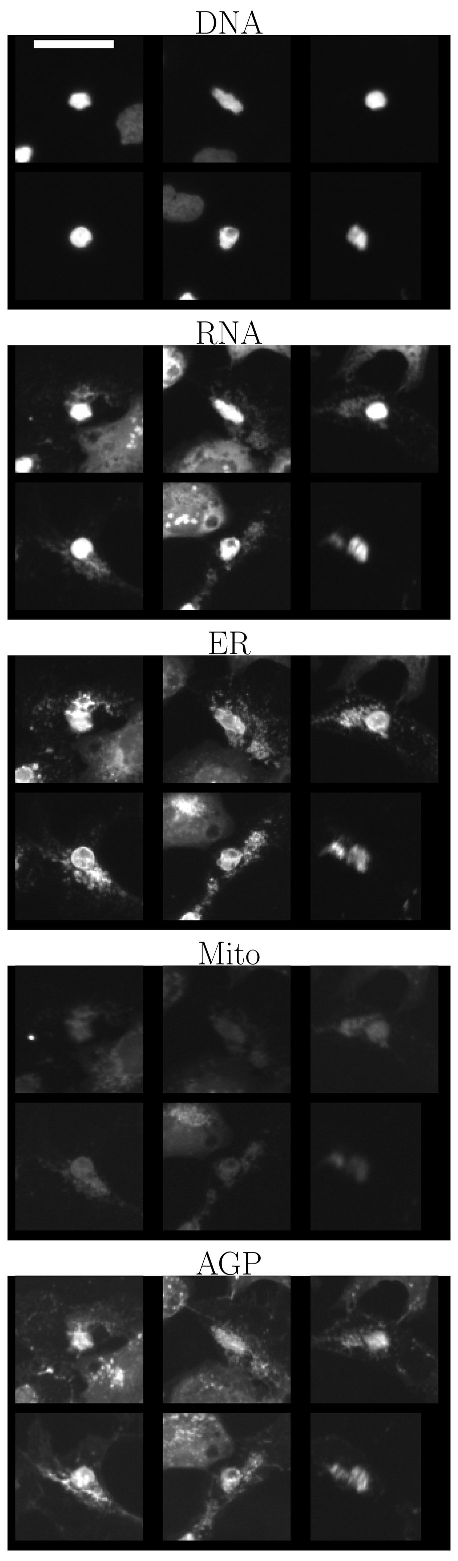

Note : Scale bar is 39.36 μm.

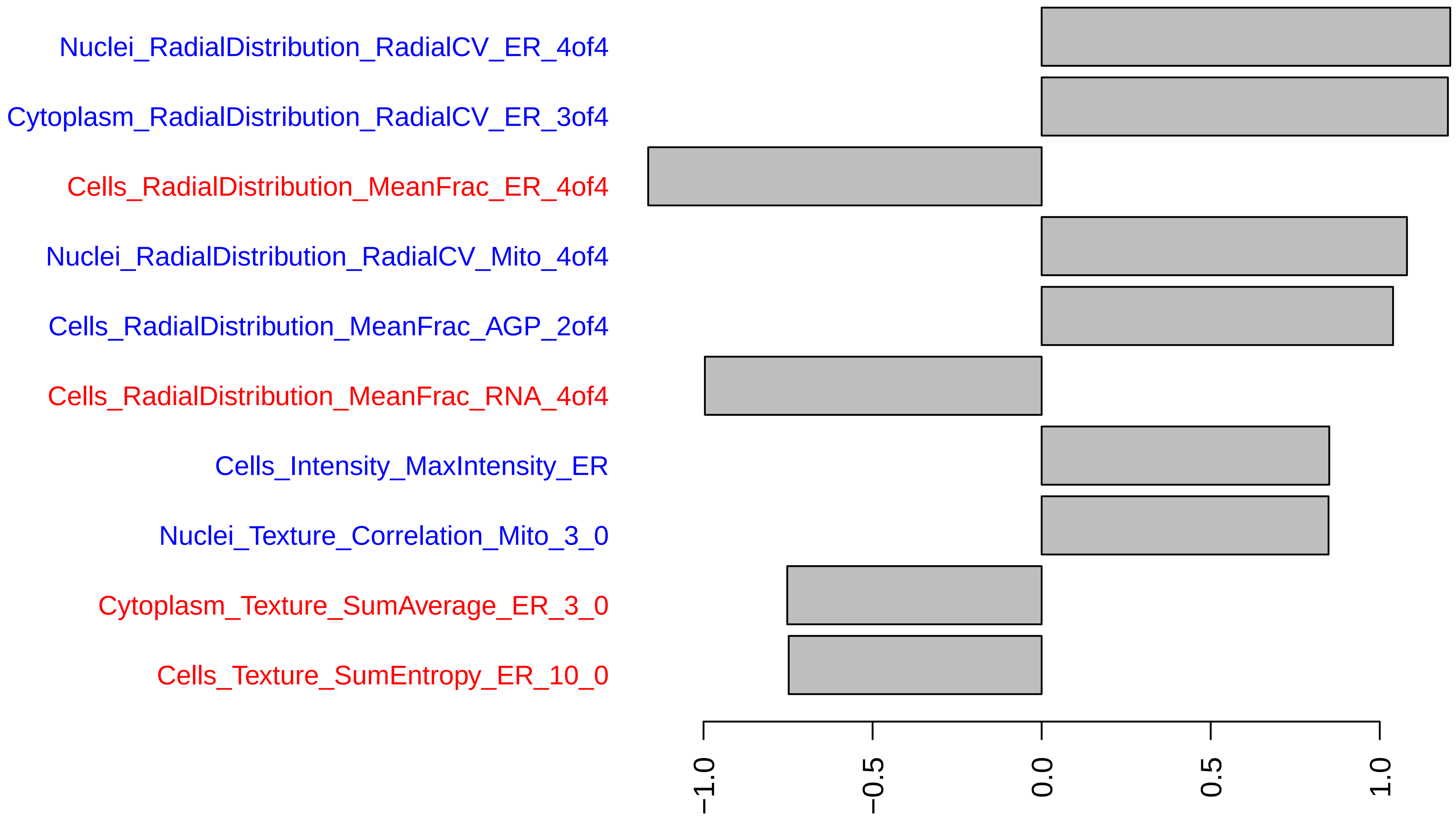

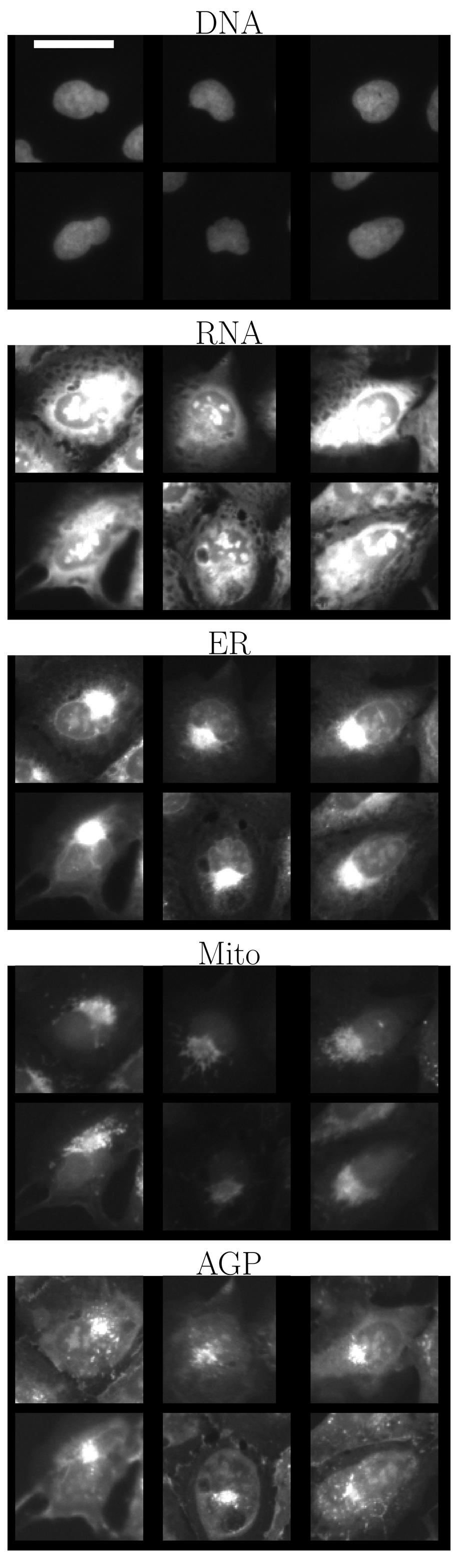

Note : Scale bar is 39.36 μm.

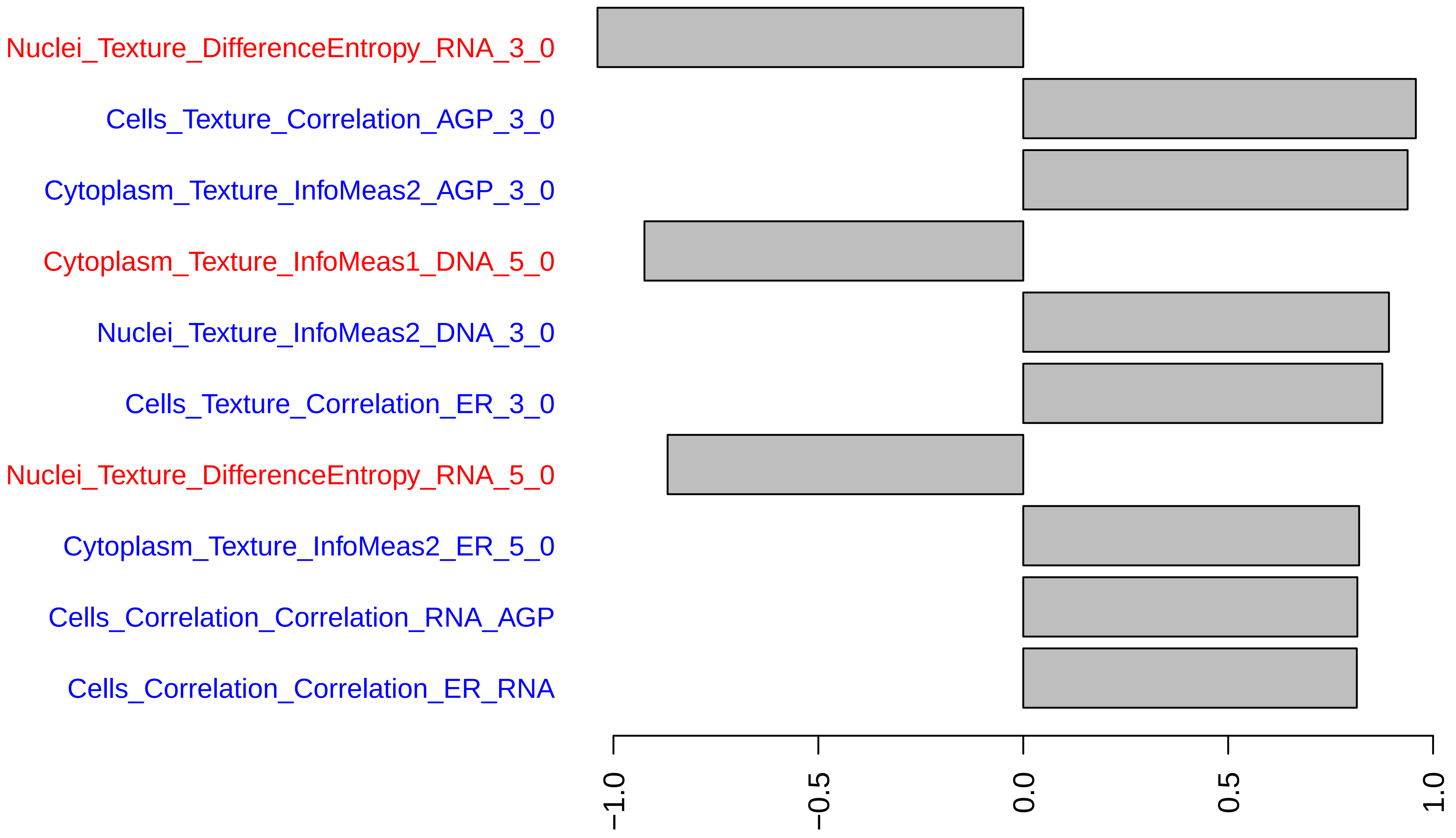

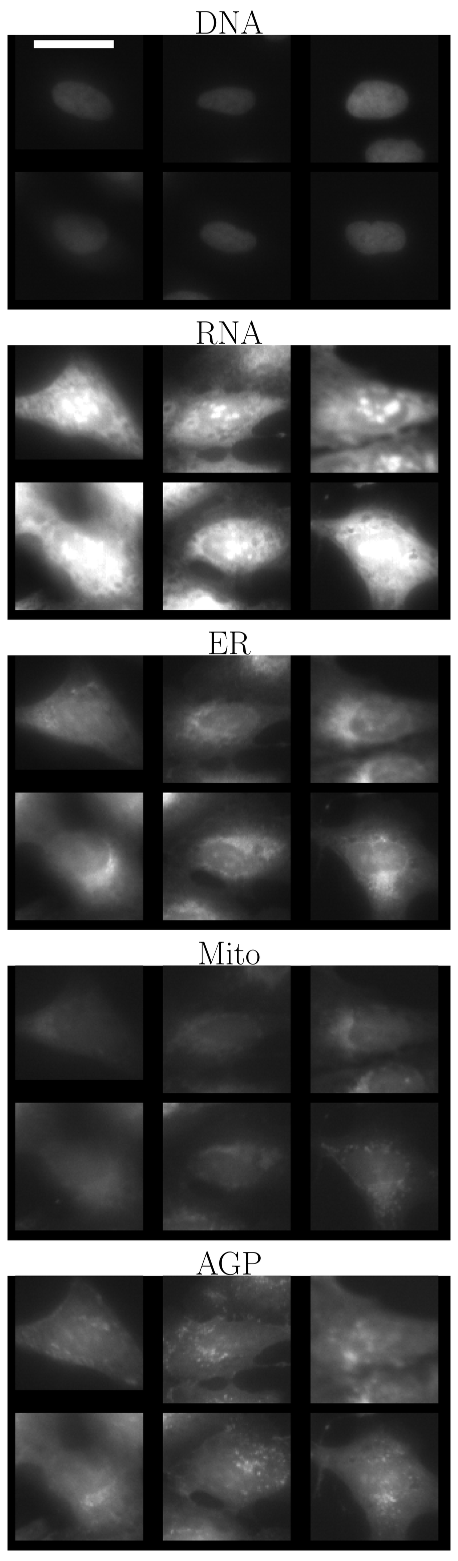

Note : Scale bar is 39.36 μm.
