# Supplementary material for: Systematic morphological profiling of human gene and allele function via Cell Painting
Source: eLife. 2017 Mar 18;6:e24060. doi: 10.7554/eLife.24060 (PMC5386591; doi:10.7554/eLife.24060)
Supplement: Supplementary file 2. — The details of the contents have been described in Figure 5. DOI: http://dx.doi.org/10.7554/eLife.24060.017 [file elife-24060-supp2.zip › Supplementary file 2/type B/7B.pdf]

	Expert Annotation	
Treatment	Pathway	Regulation Type
AKT1_E17K	Canonical PI3K/AKT	Activator
AKT3_E17K	Canonical PI3K/AKT	Activator
AKT3_WT.2	Canonical PI3K/AKT	Activator
CDC42_T17N	Canonical Cytoskeletal Re-org	Inhibitor
RAC1_T17N	Canonical Cytoskeletal Re-org	Inhibitor

Nuclei_Intensity_IntegratedIntensity_DNA

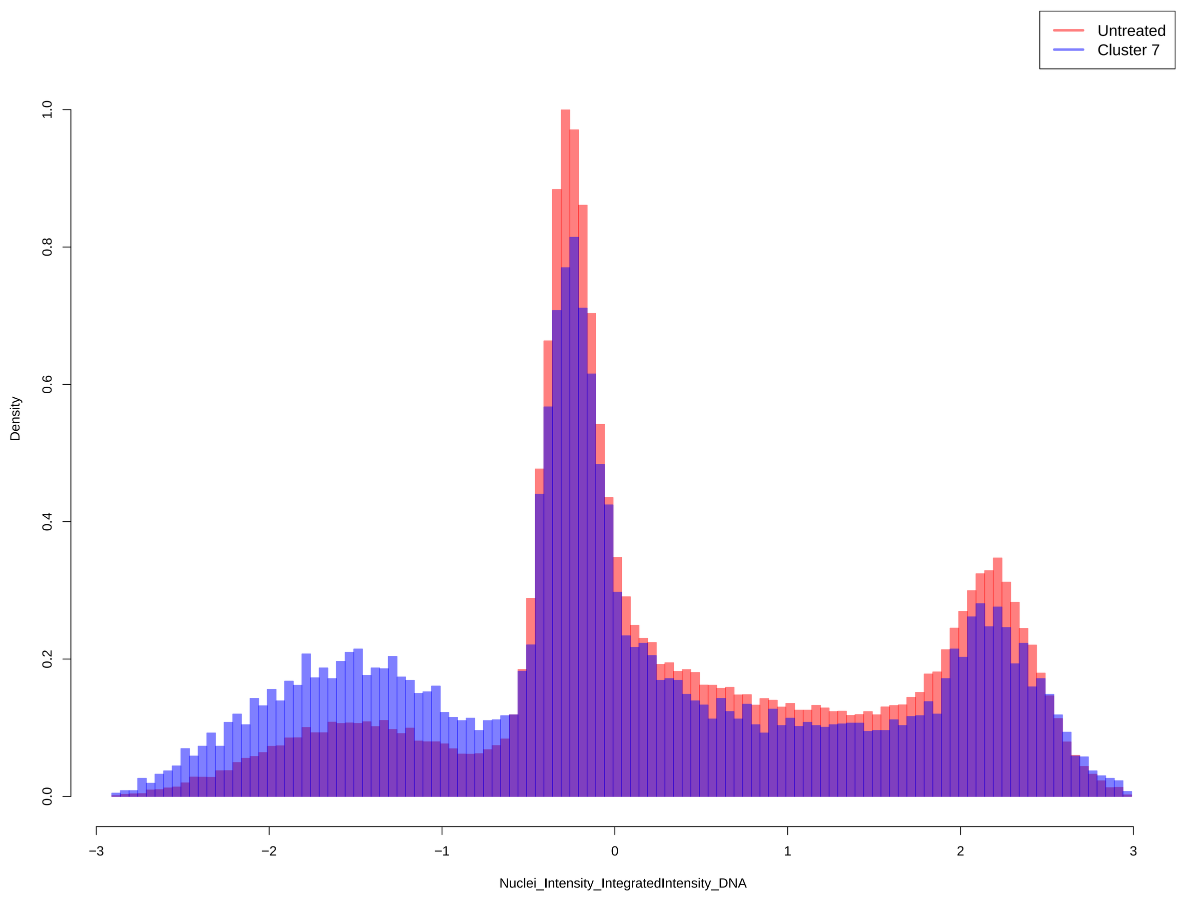

Different categories of cells in the cluster :

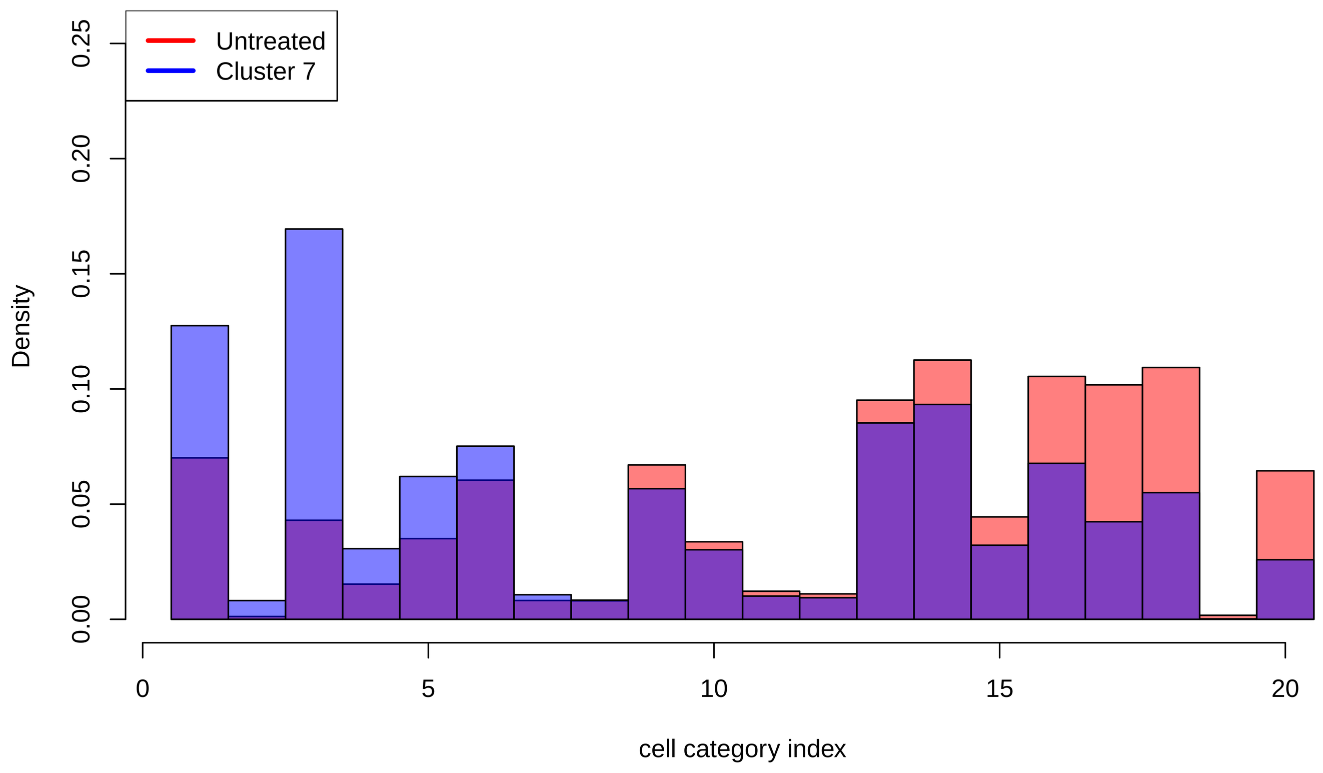

Different categories of cells in single genes in the cluster :

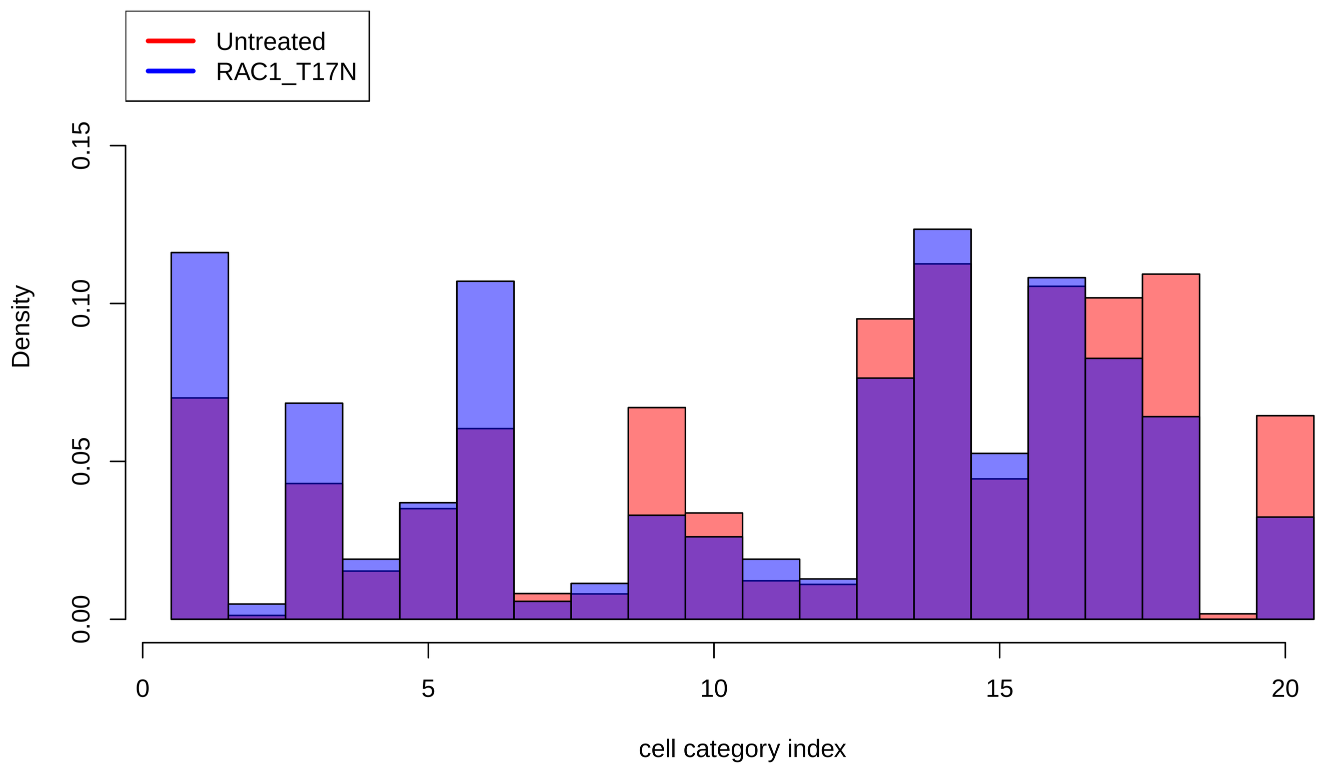

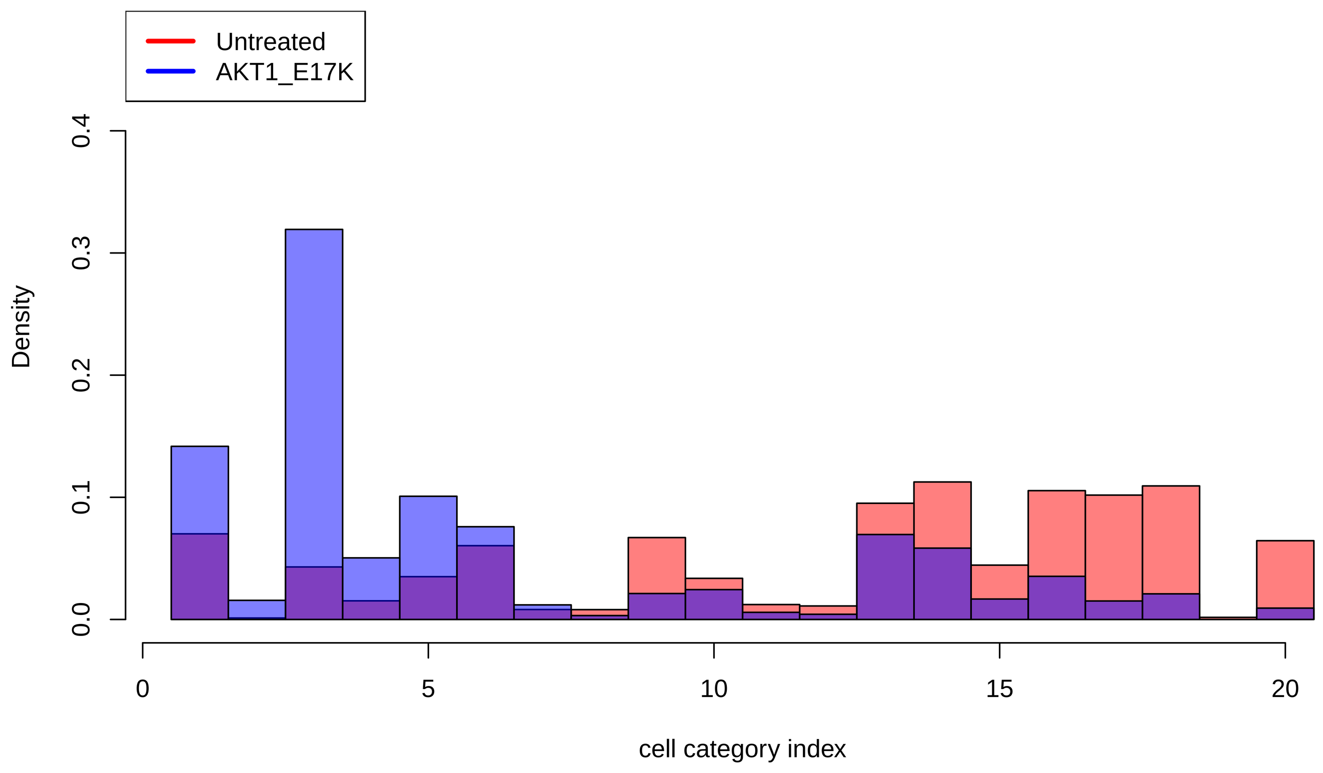

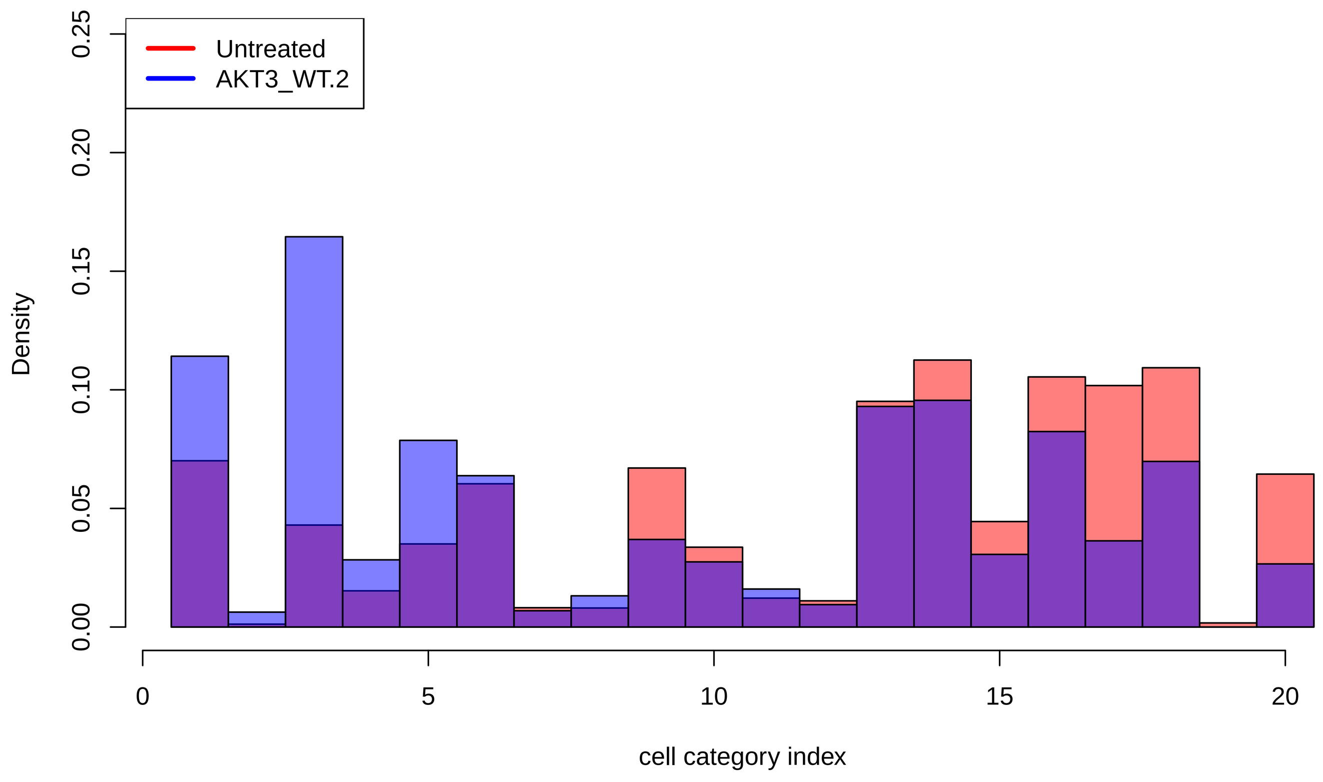

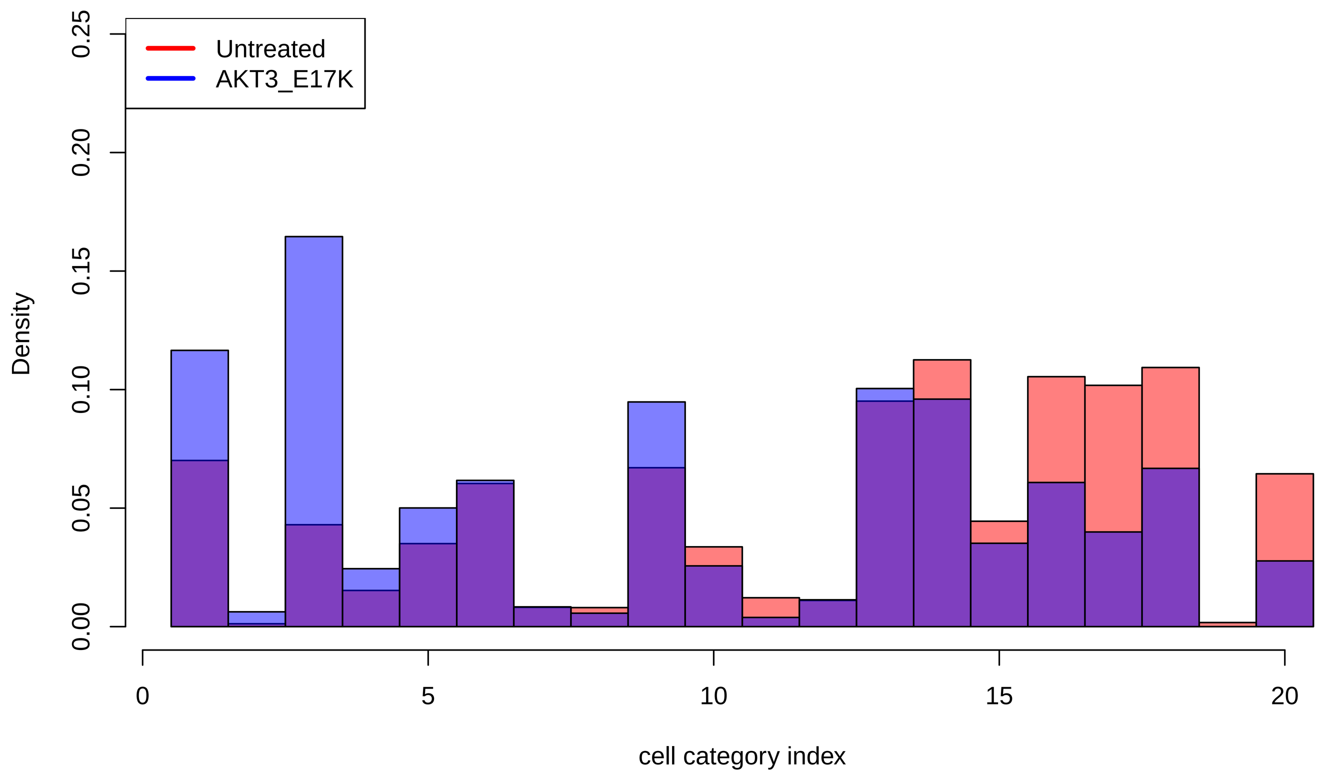
Untreated
AKT3_E17K
Density
0.00
0.05
0.10
0.15
0.20
0.25
0
5
10
15
20
cell category index

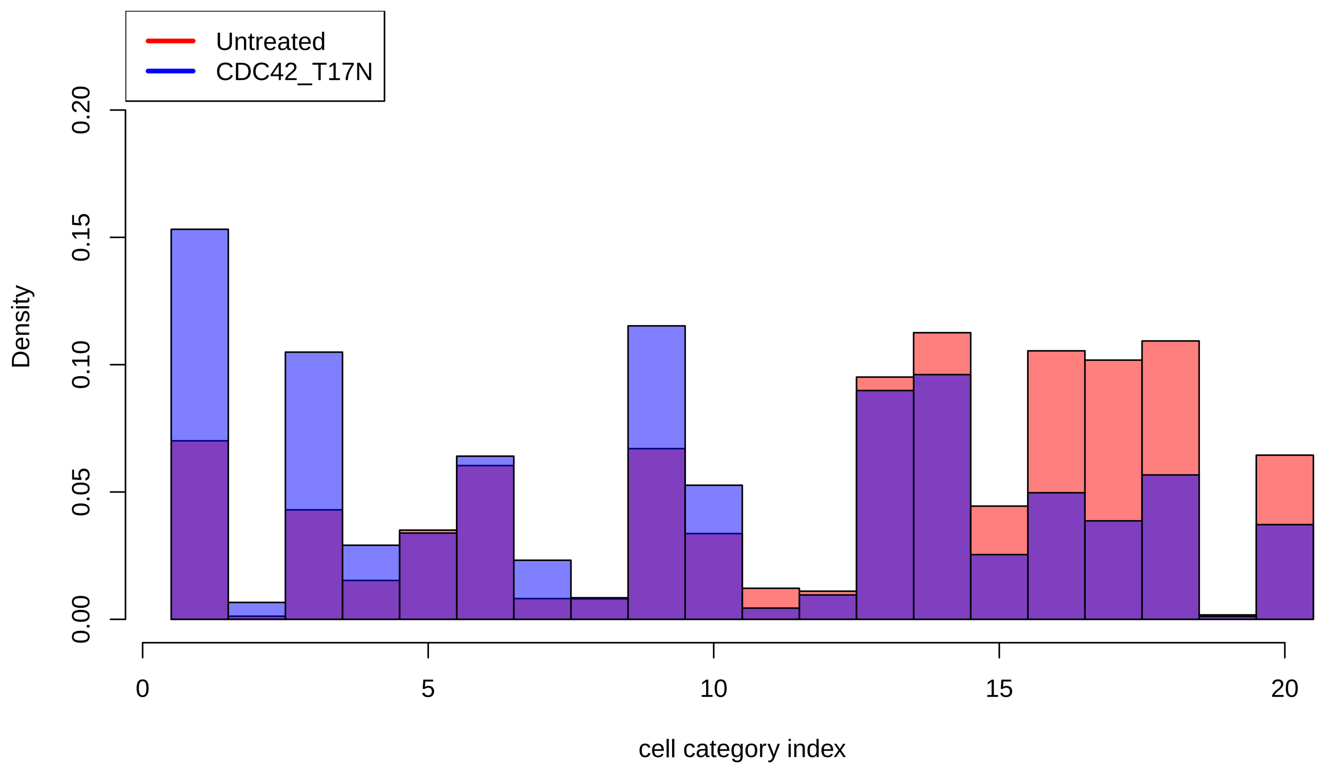
Untreated
CDC42_T17N
Density
0.00
0.05
0.10
0.15
0.20
0
5
10
15
20
cell category index

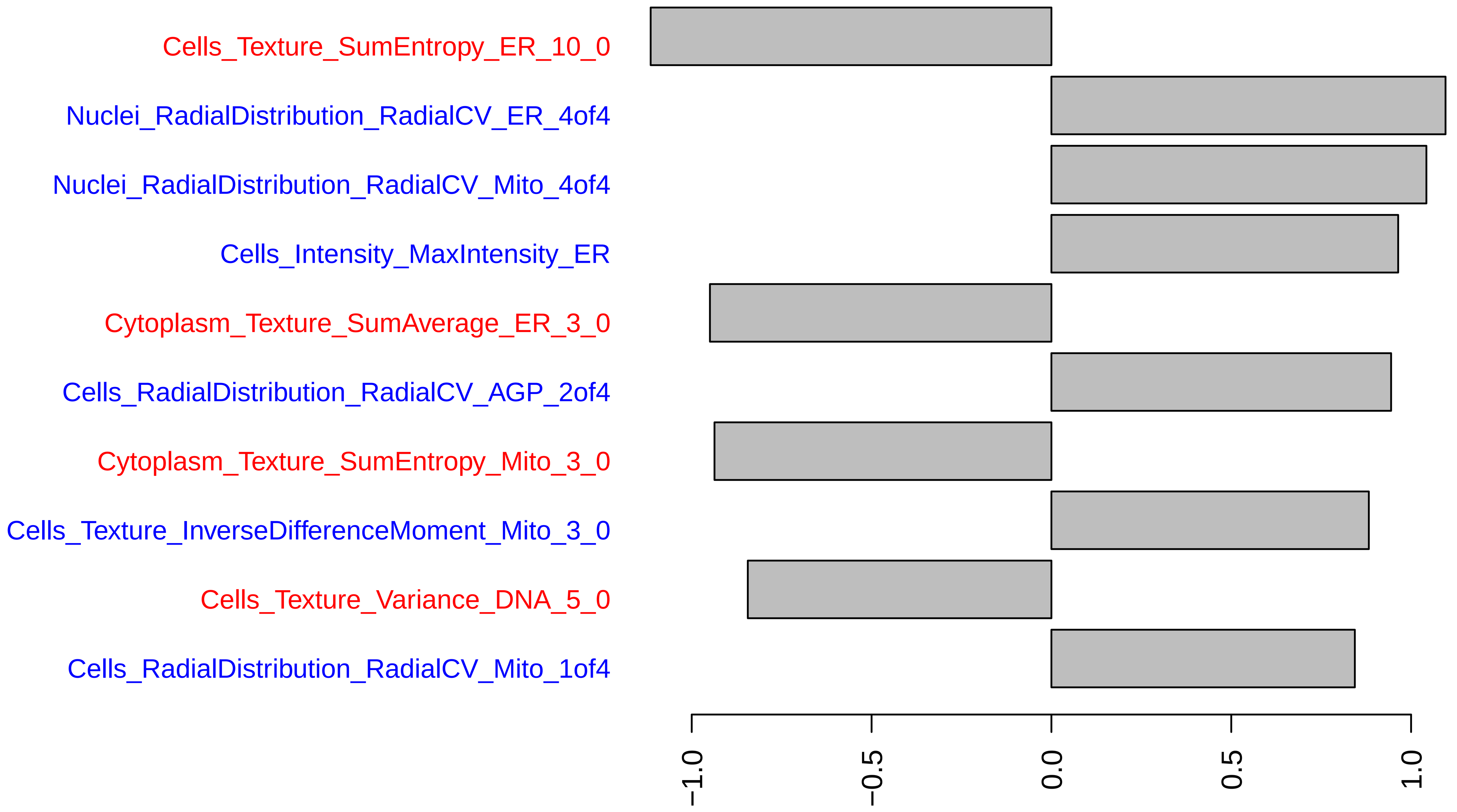

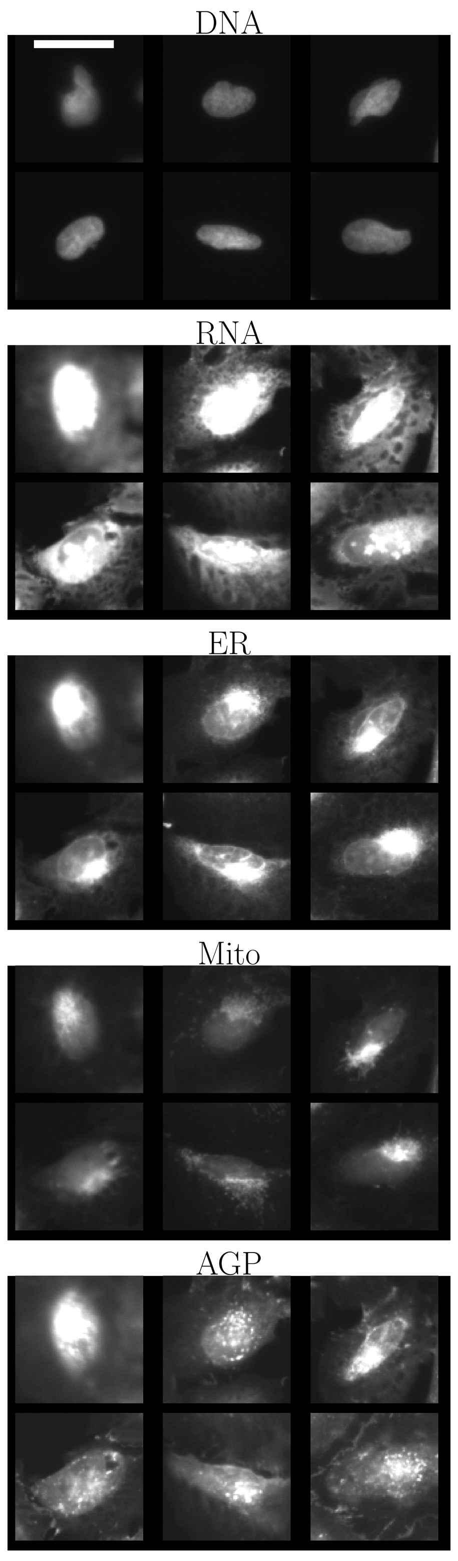

Note : Scale bar is 39.36 μm.

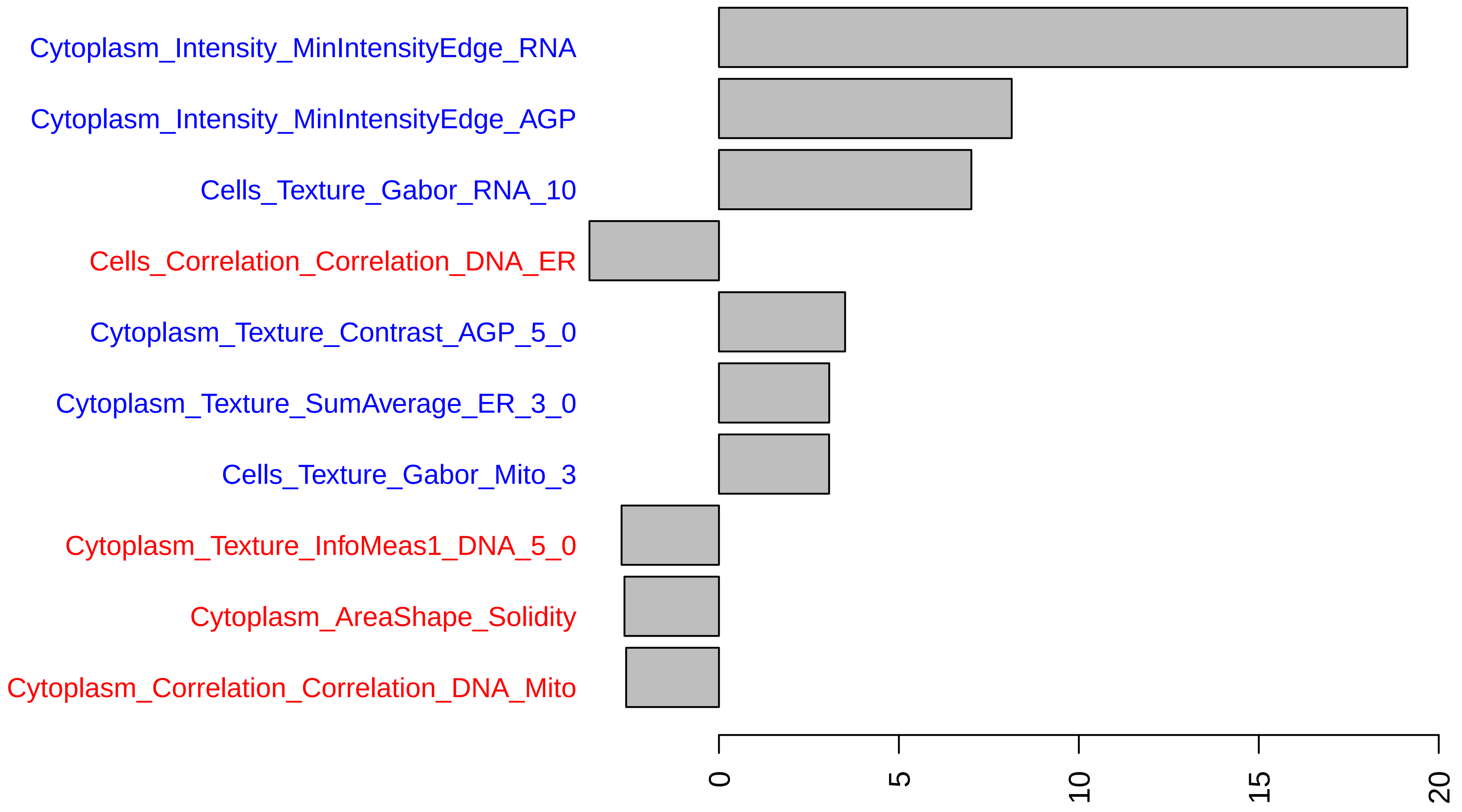

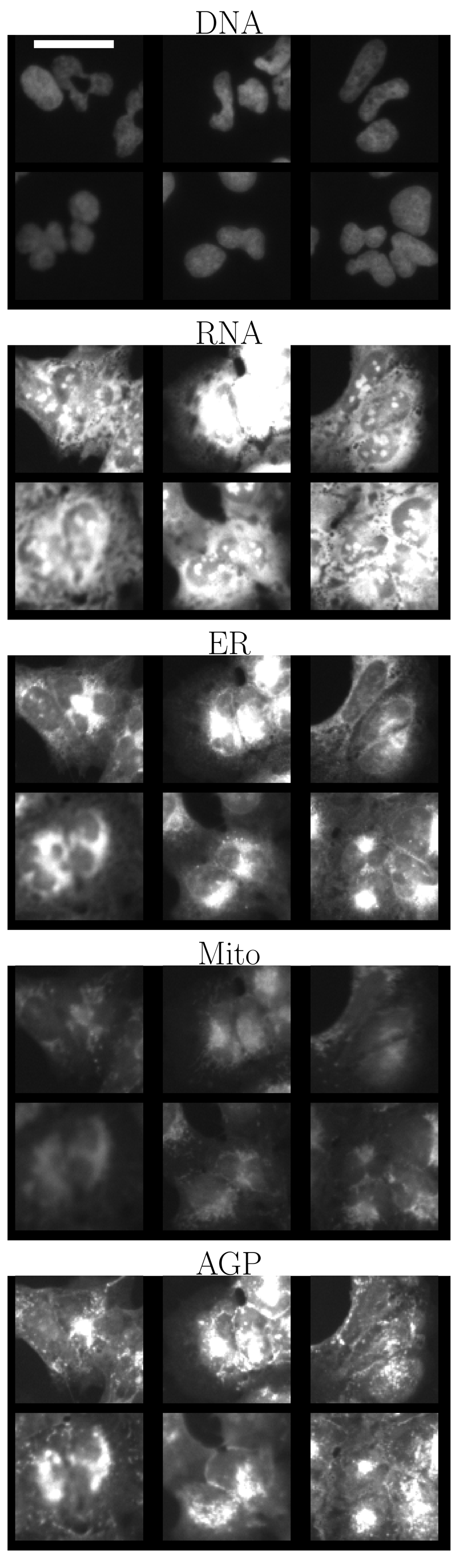

Note : Scale bar is 39.36 μm.

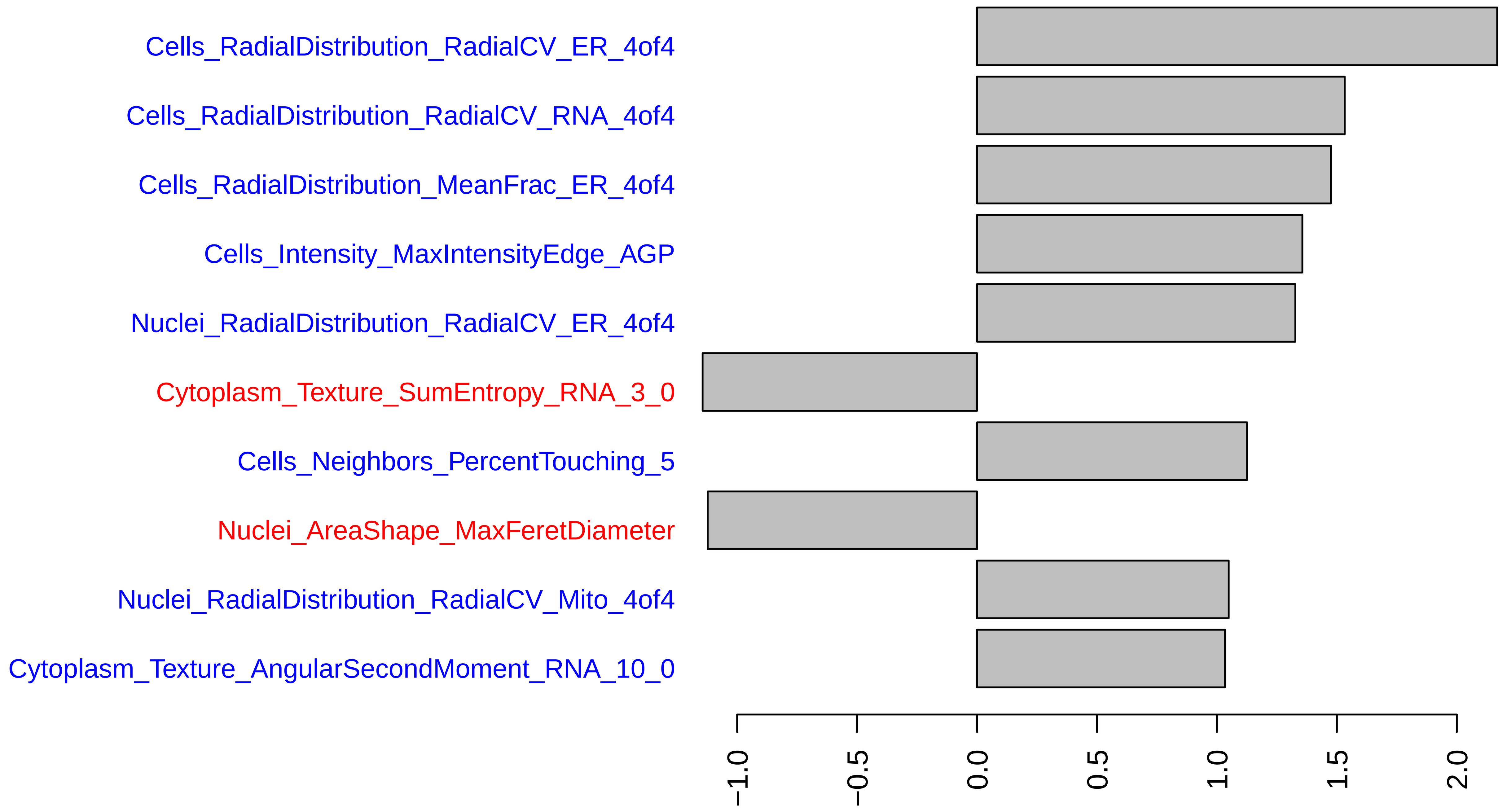

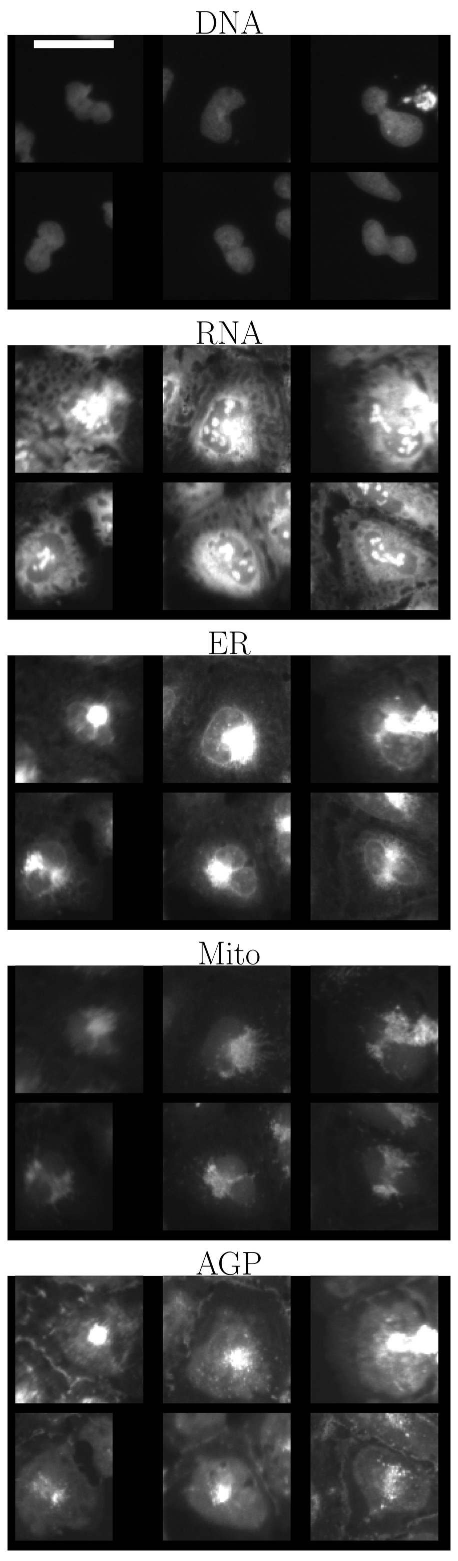

Note : Scale bar is 39.36 μm.

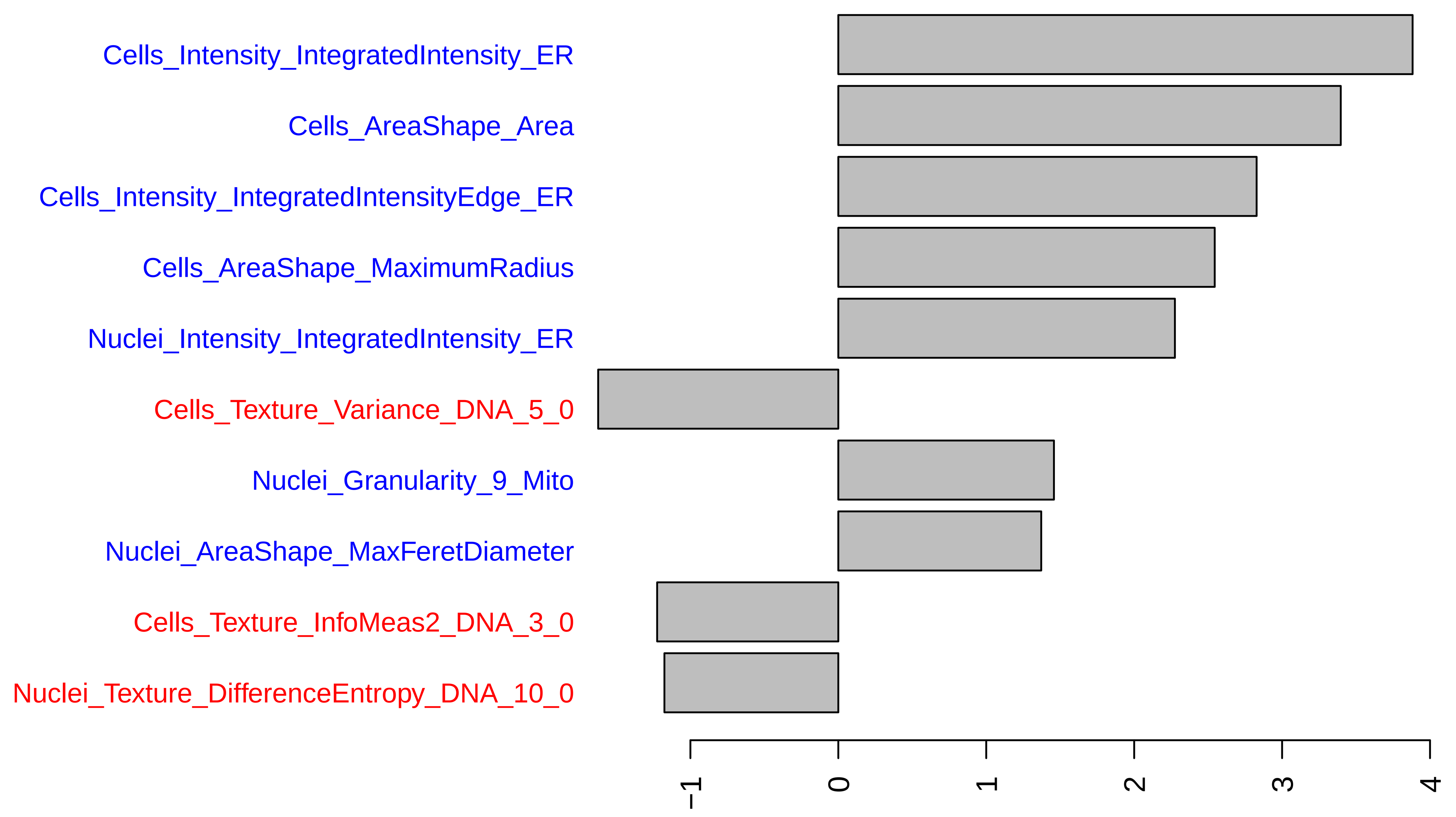

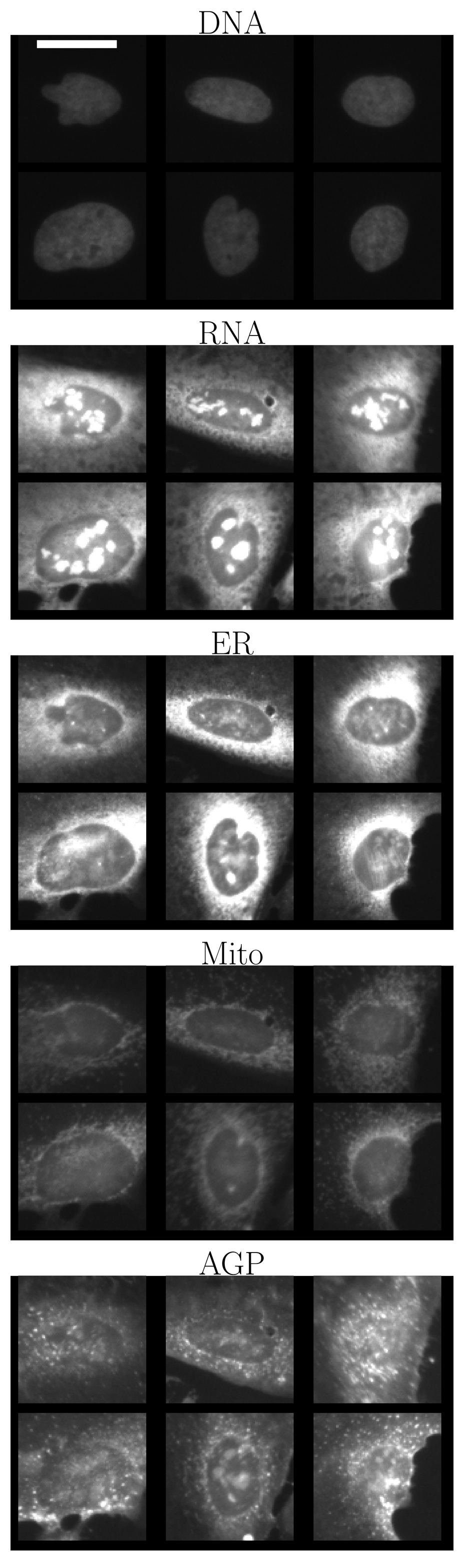

Note : Scale bar is 39.36 μm.

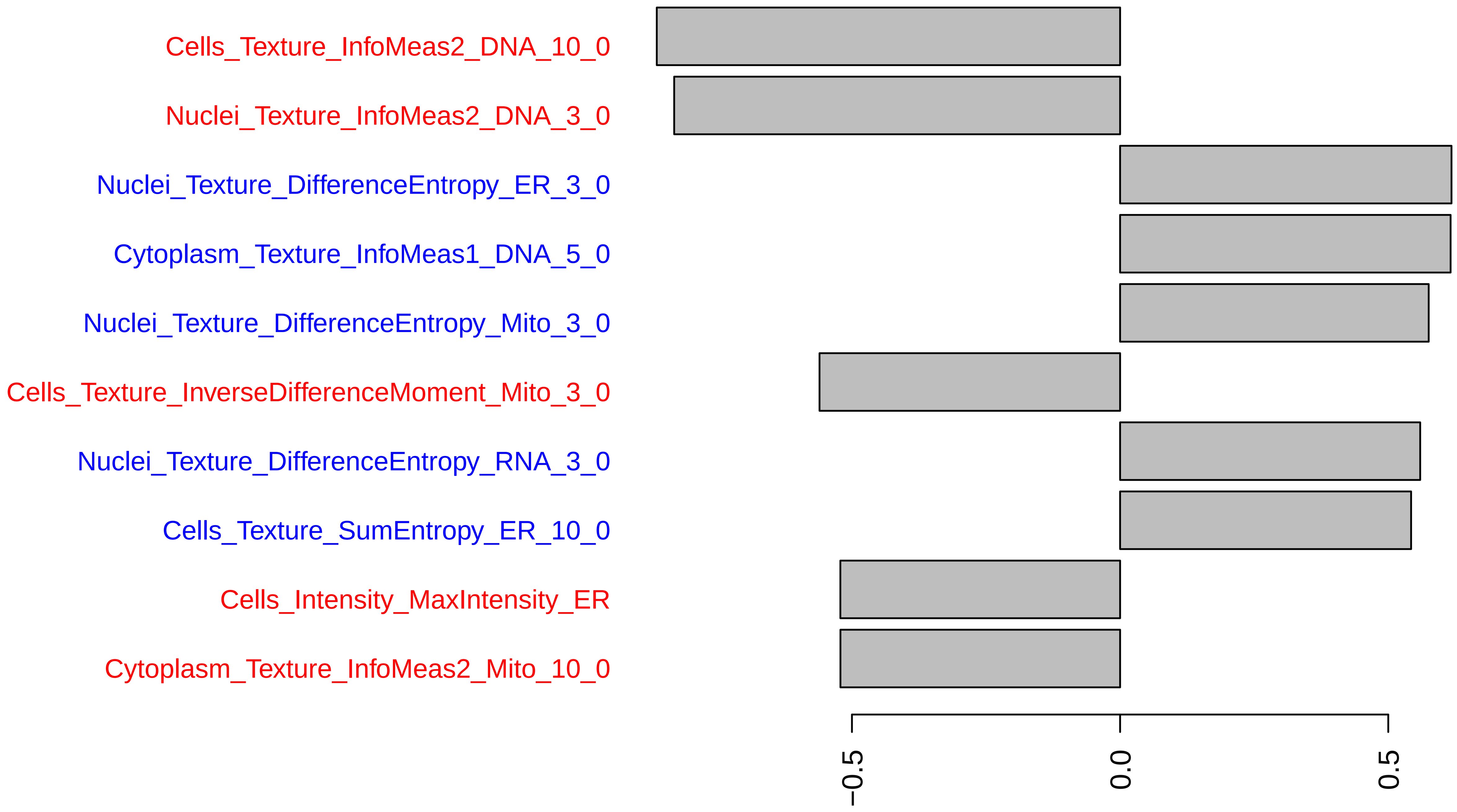

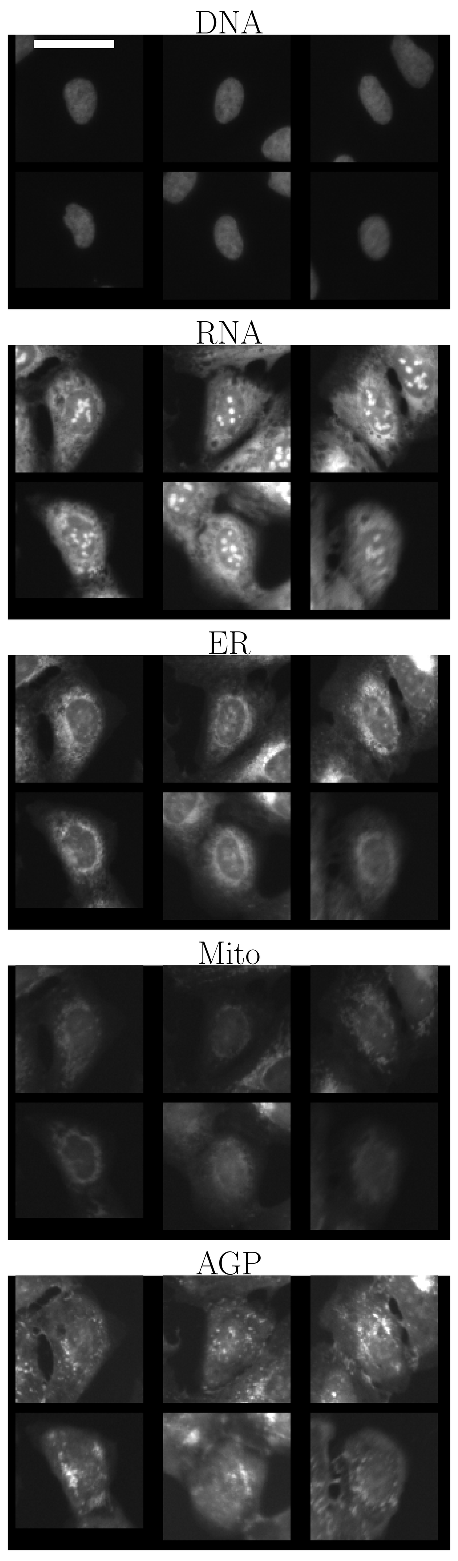

Note : Scale bar is 39.36 μm.

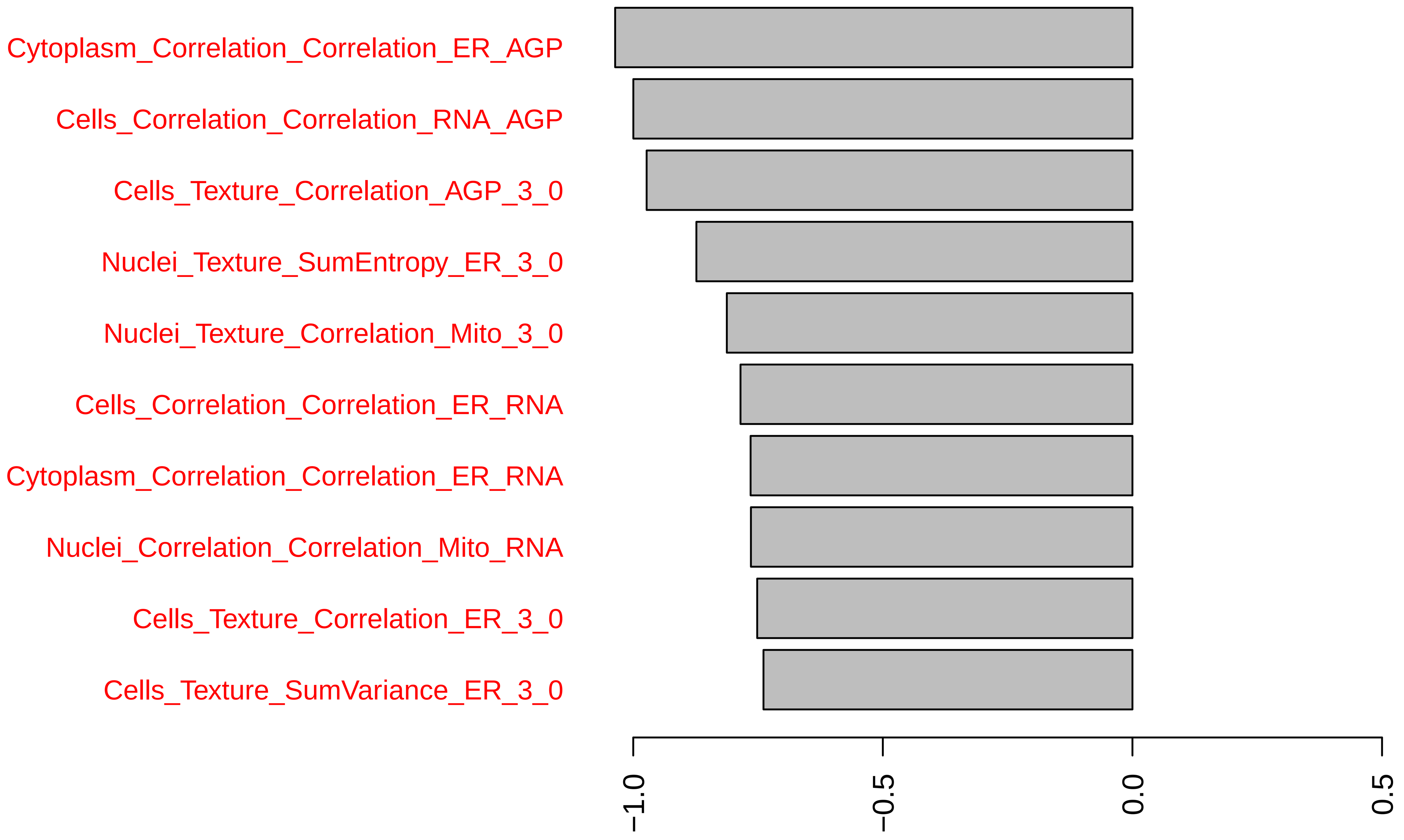

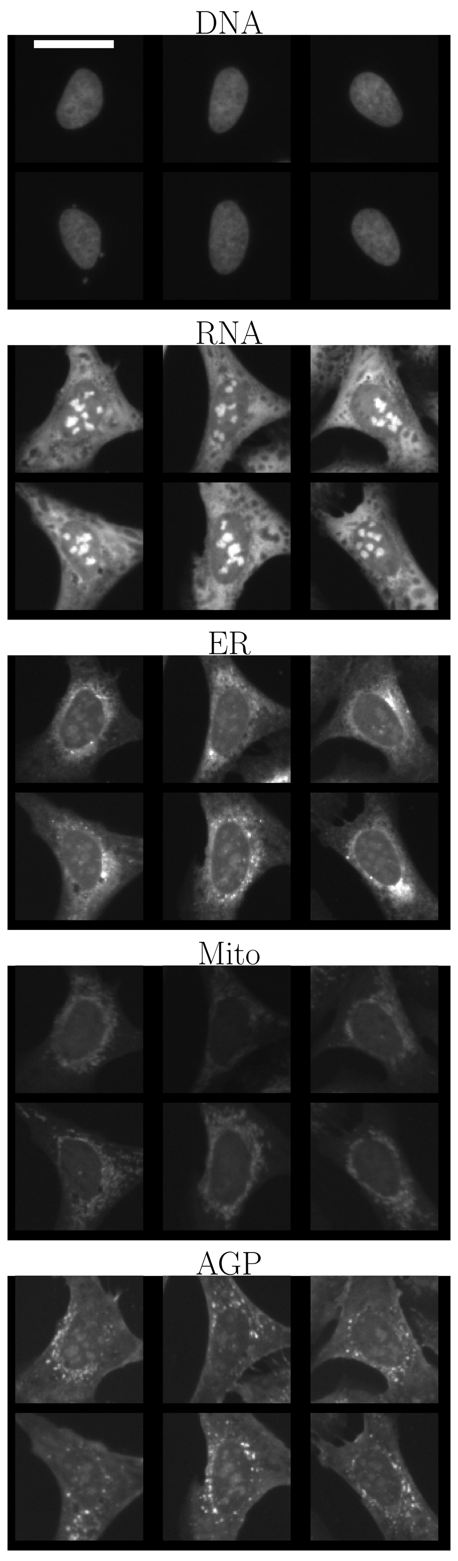

Note : Scale bar is 39.36 μm.

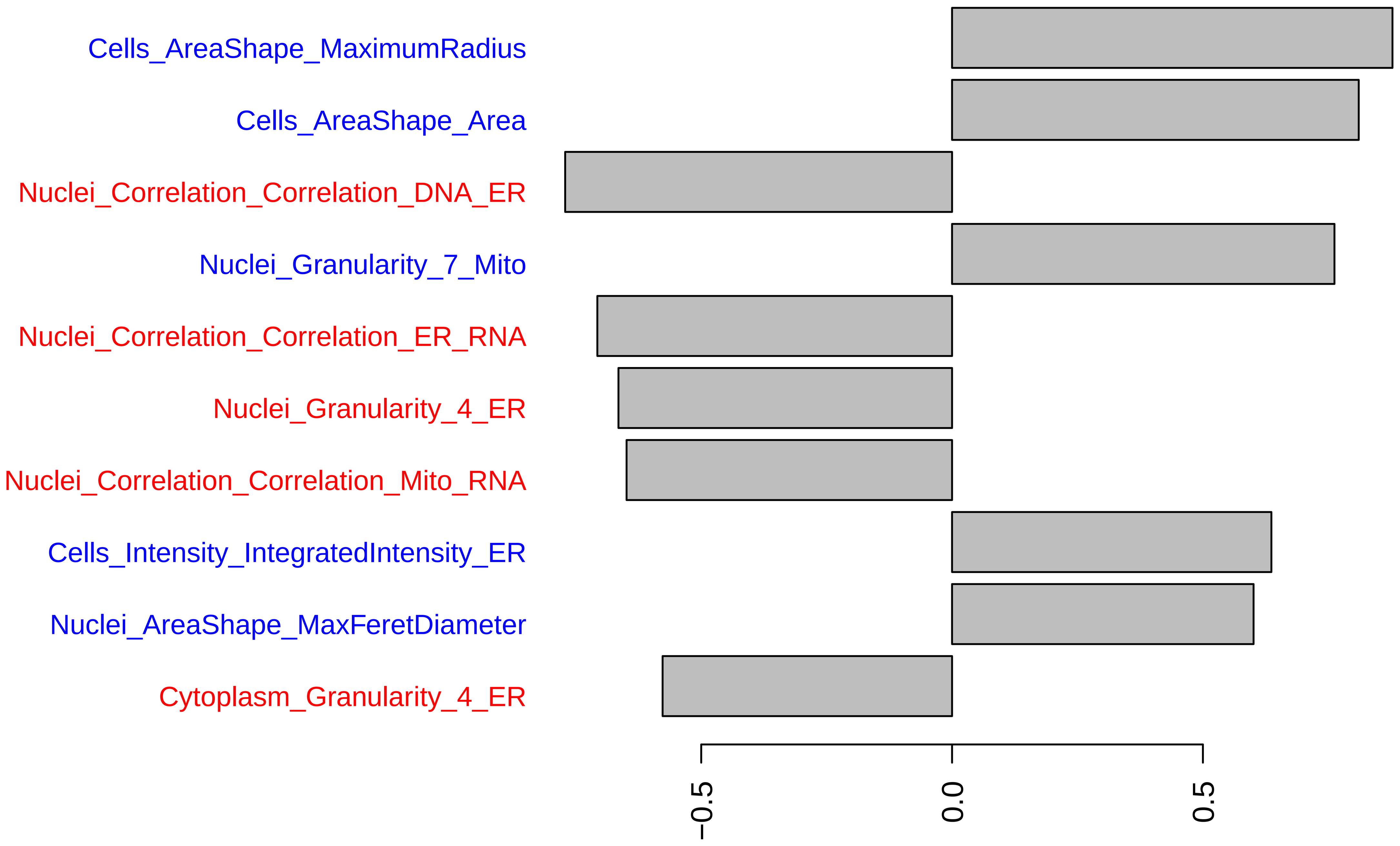

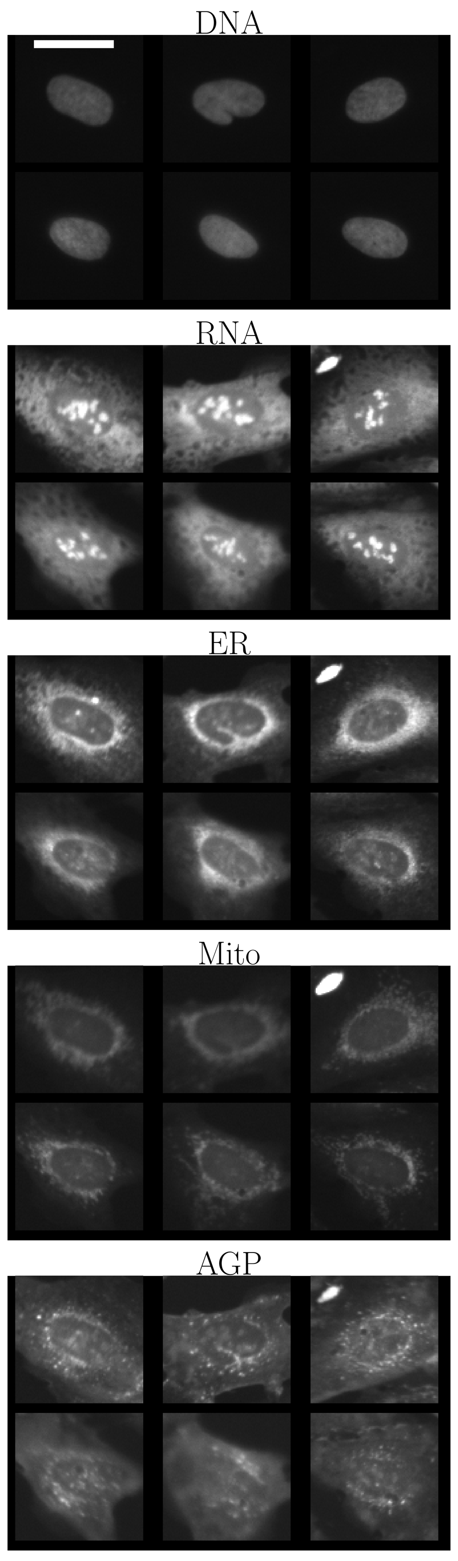

Note : Scale bar is 39.36 μm.

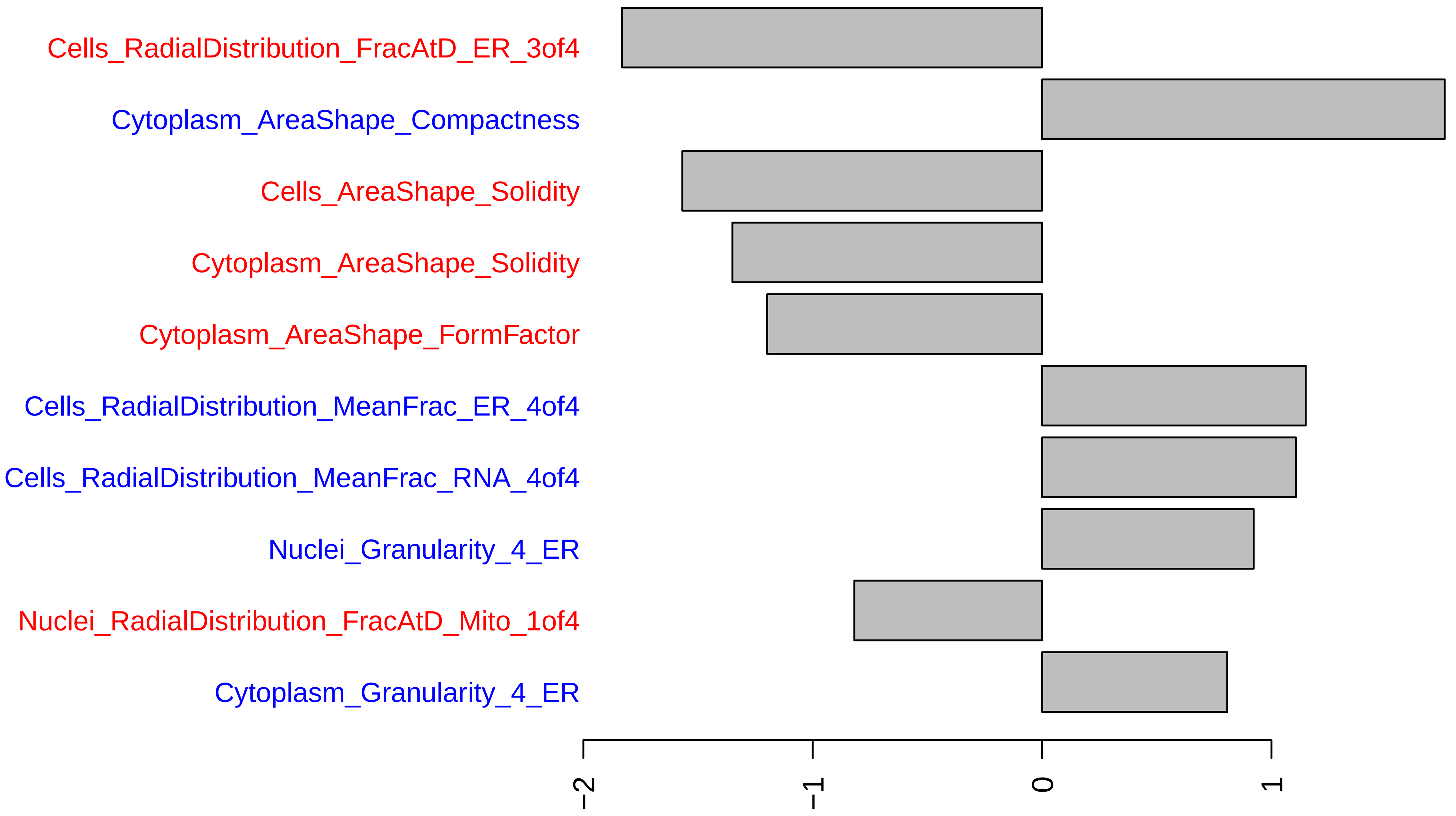

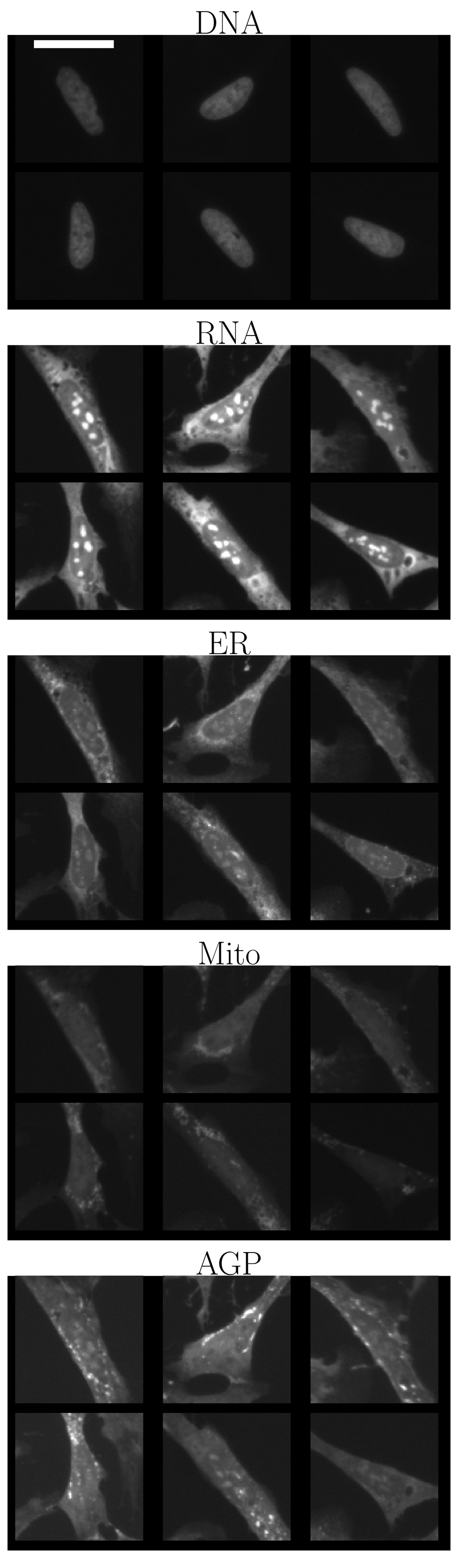

Note : Scale bar is 39.36 μm.
