# Supplementary figures and images for: Systematic morphological profiling of human gene and allele function via Cell Painting
Source: eLife. 2017 Mar 18;6:e24060. doi: 10.7554/eLife.24060 (PMC5386591; doi:10.7554/eLife.24060)

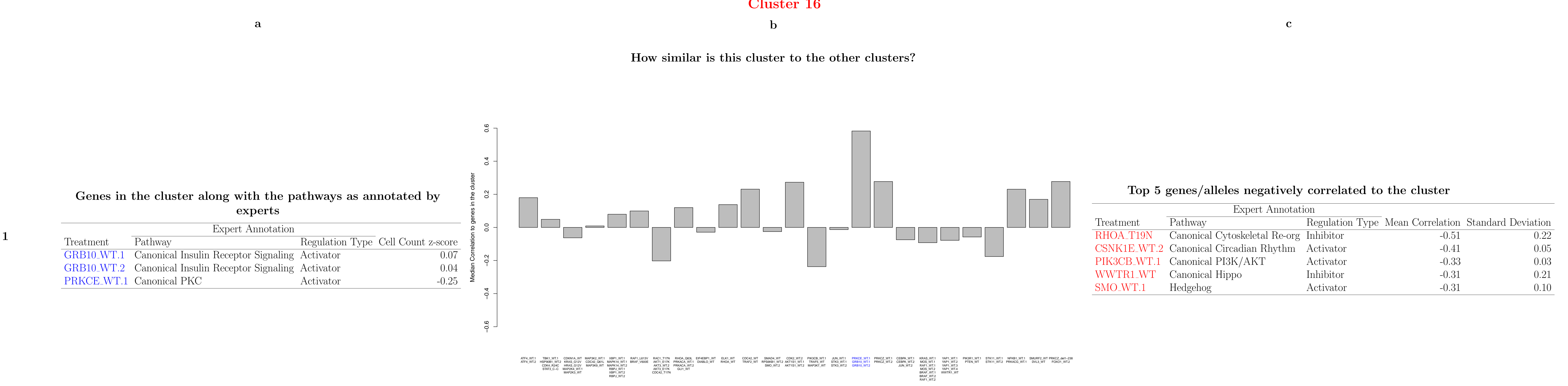

Supplement: Supplementary file 2. — The details of the contents have been described in Figure 5. DOI: http://dx.doi.org/10.7554/eLife.24060.017 [file elife-24060-supp2.zip › Supplementary file 2/type A/16A.pdf]

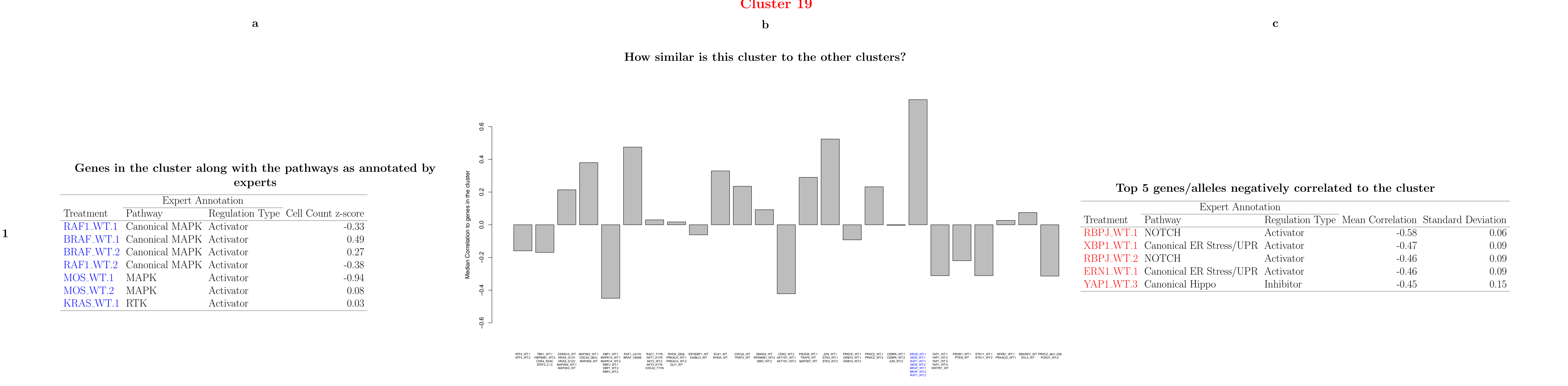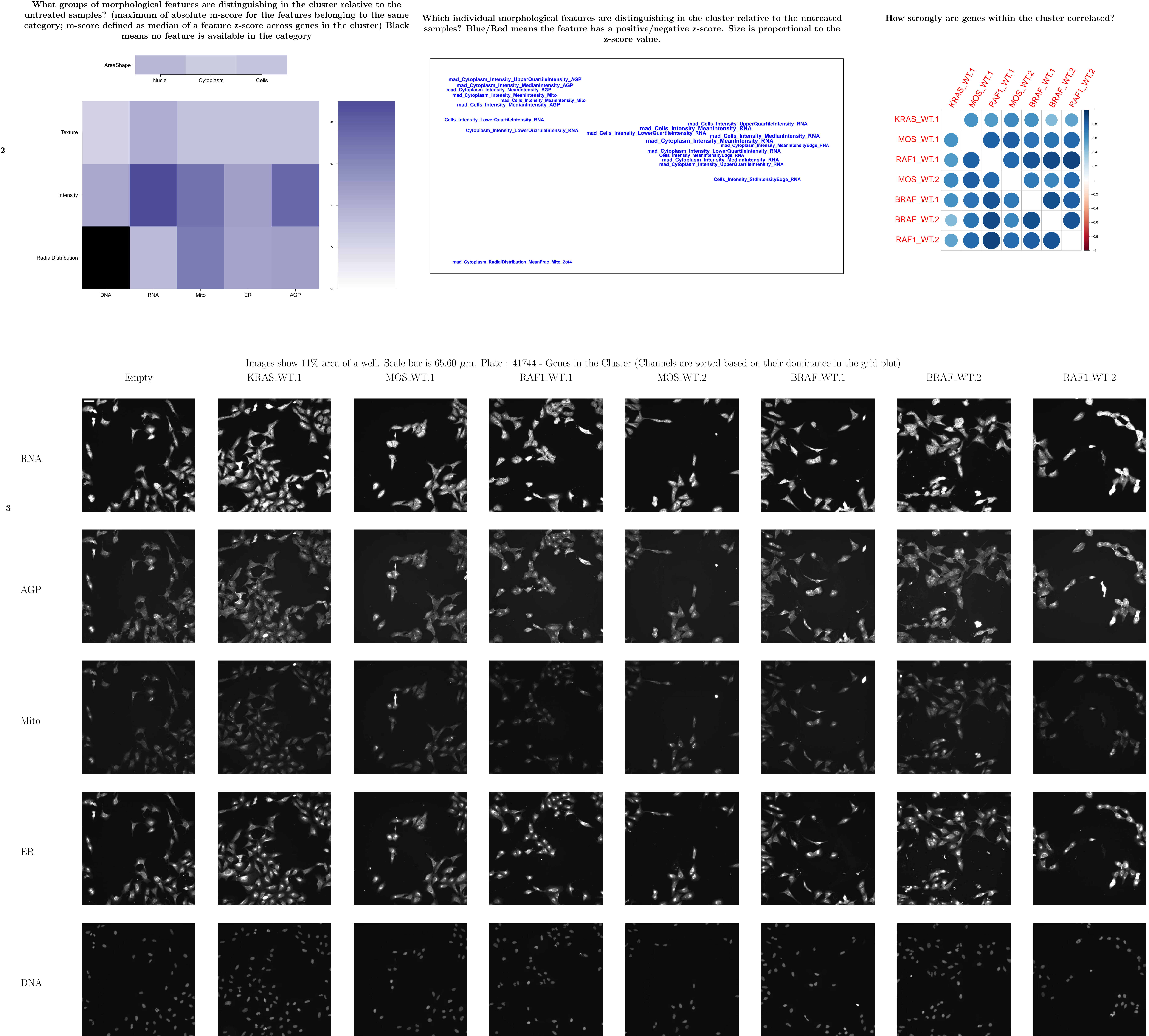

Supplement: Supplementary file 2. — The details of the contents have been described in Figure 5. DOI: http://dx.doi.org/10.7554/eLife.24060.017 [file elife-24060-supp2.zip › Supplementary file 2/type A/19A.pdf]

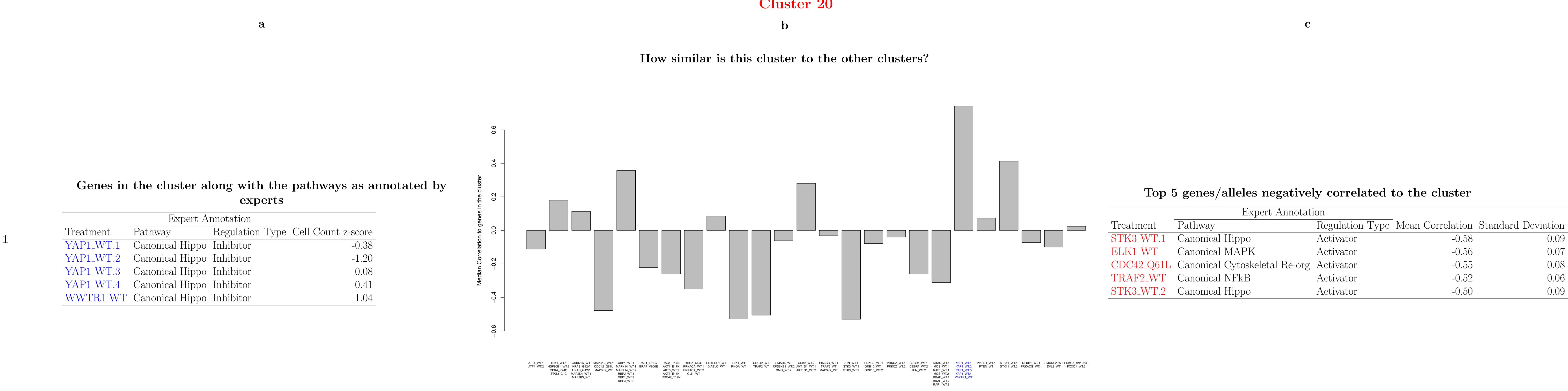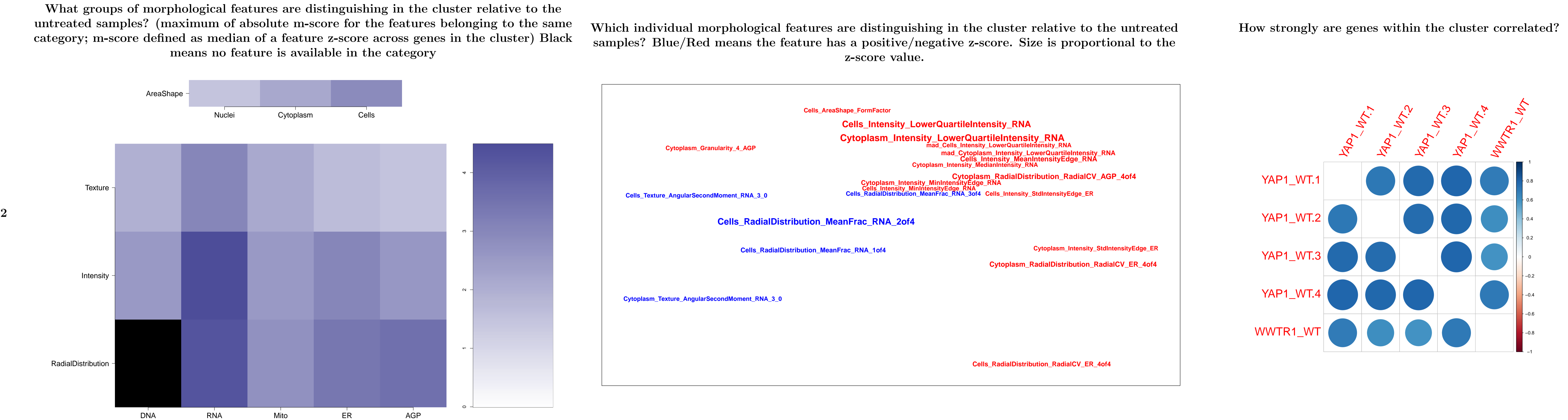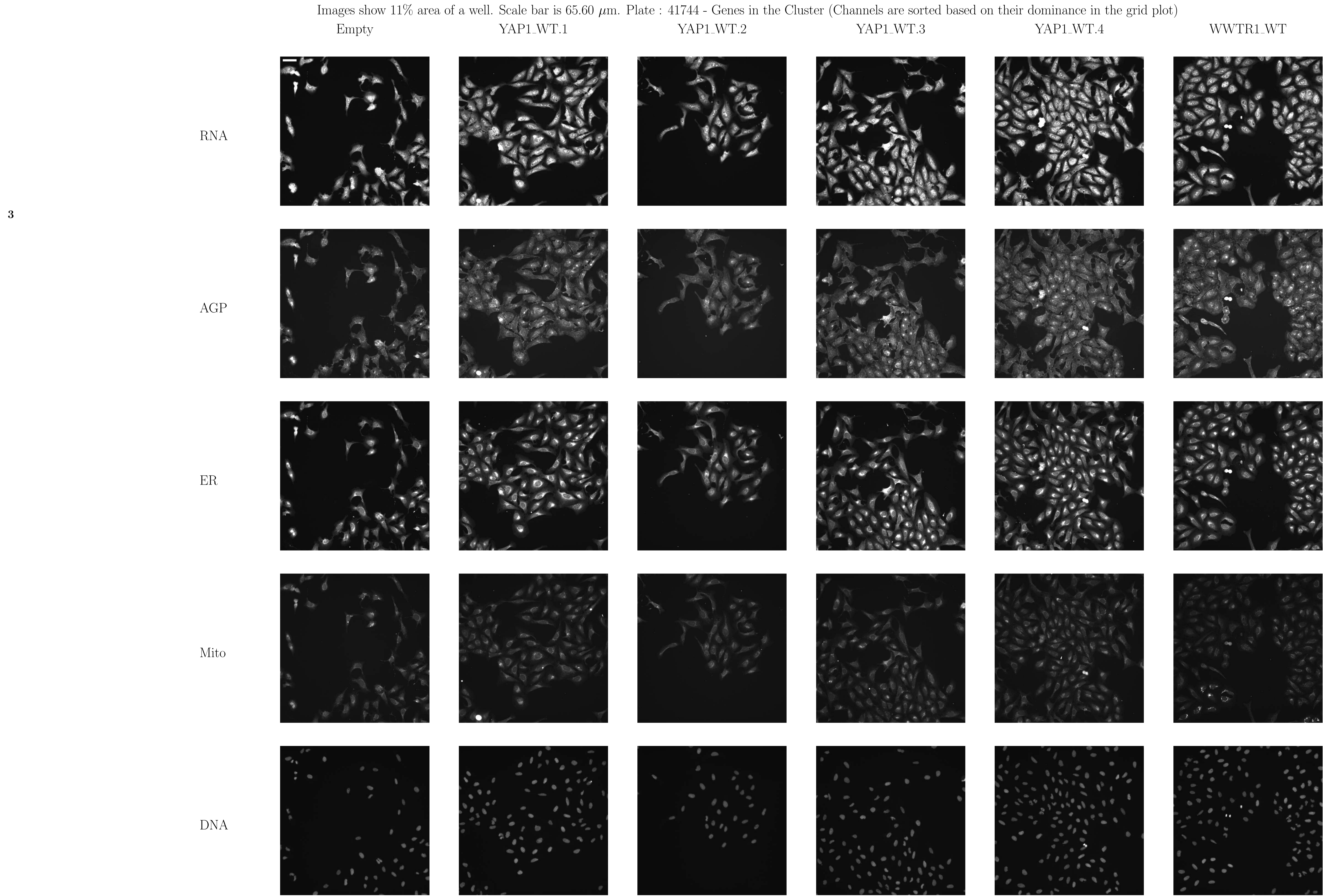

Supplement: Supplementary file 2. — The details of the contents have been described in Figure 5. DOI: http://dx.doi.org/10.7554/eLife.24060.017 [file elife-24060-supp2.zip › Supplementary file 2/type A/20A.pdf]

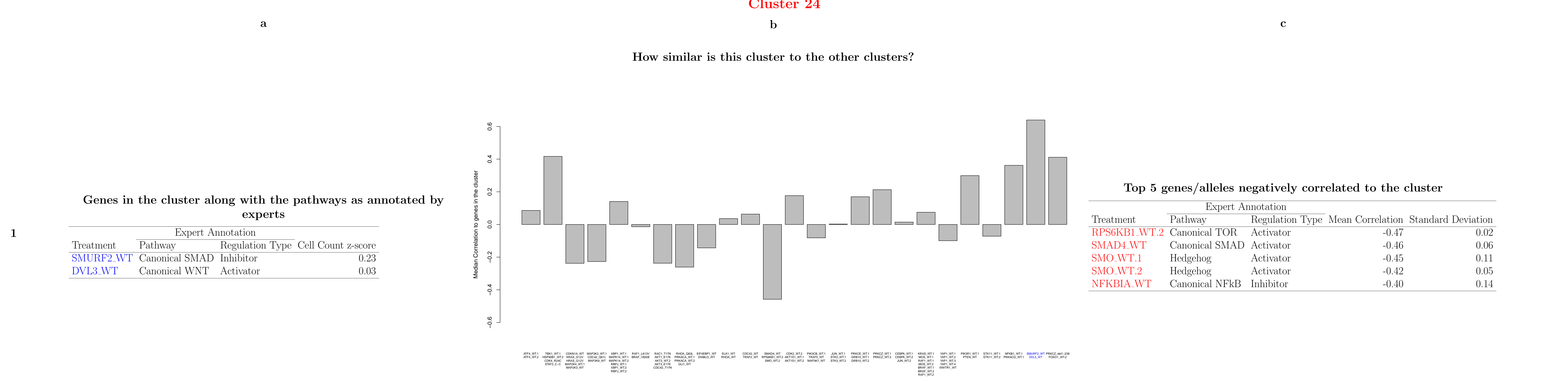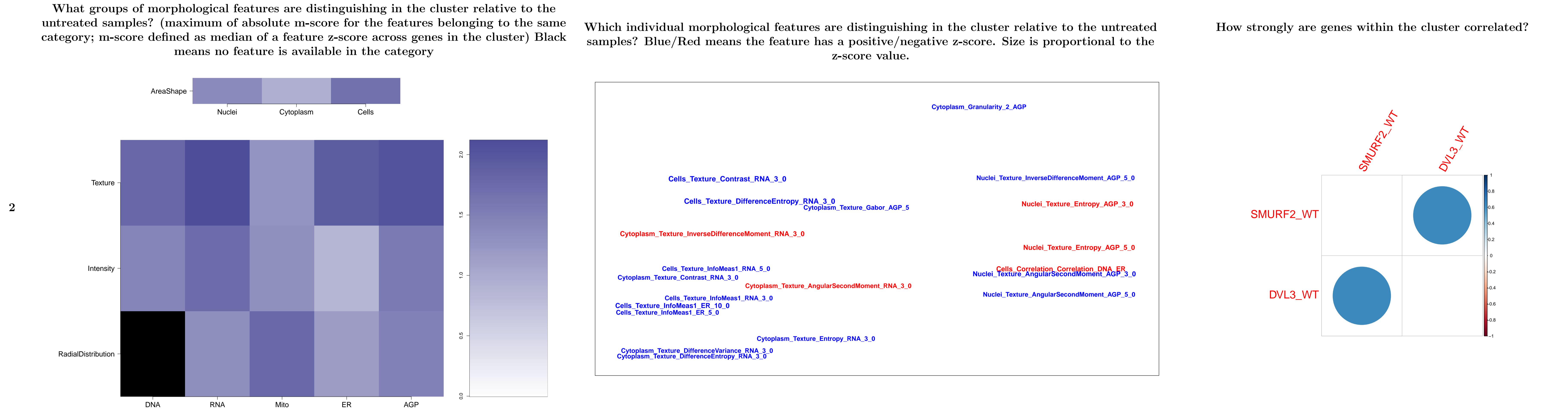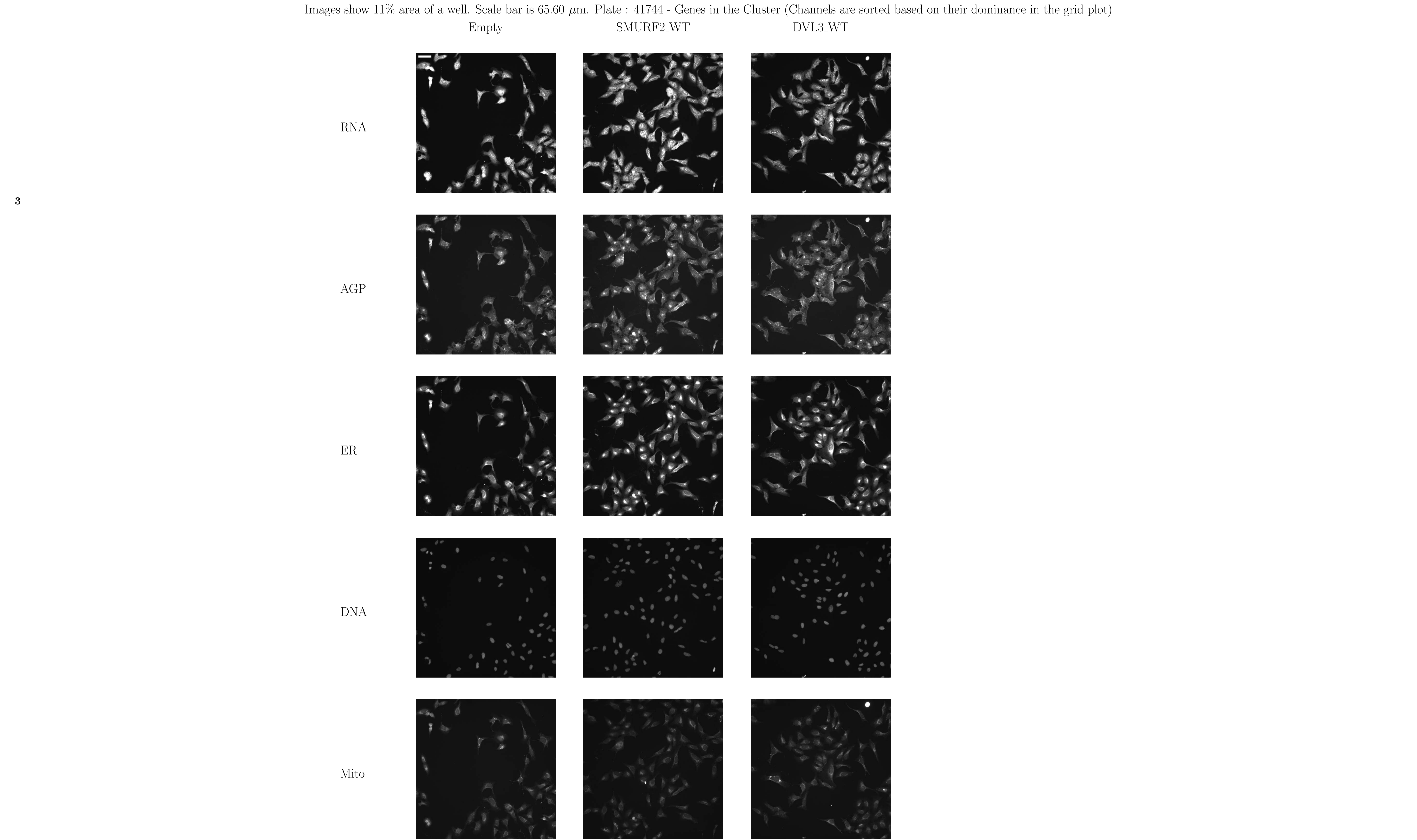

Supplement: Supplementary file 2. — The details of the contents have been described in Figure 5. DOI: http://dx.doi.org/10.7554/eLife.24060.017 [file elife-24060-supp2.zip › Supplementary file 2/type A/24A.pdf]

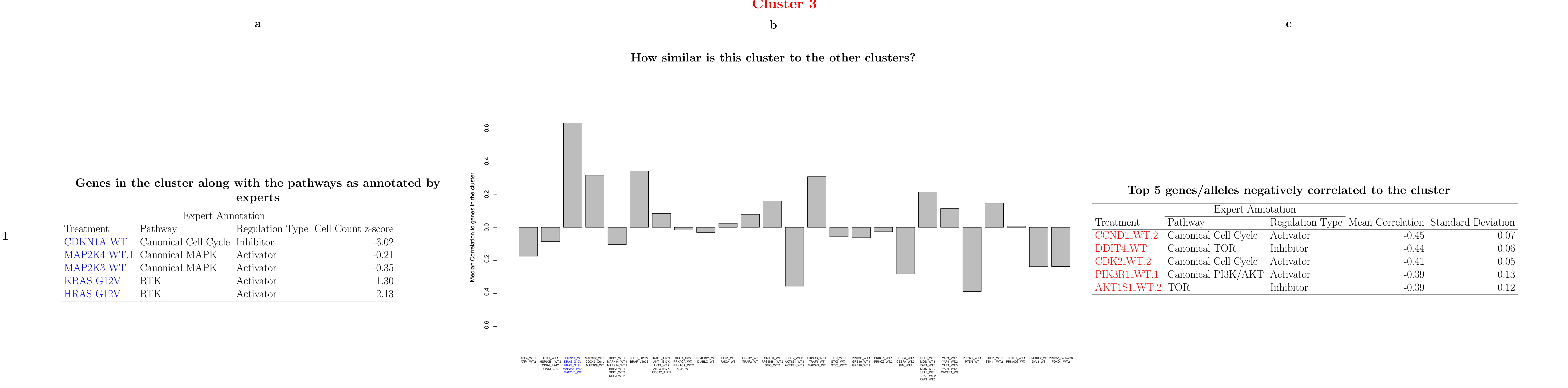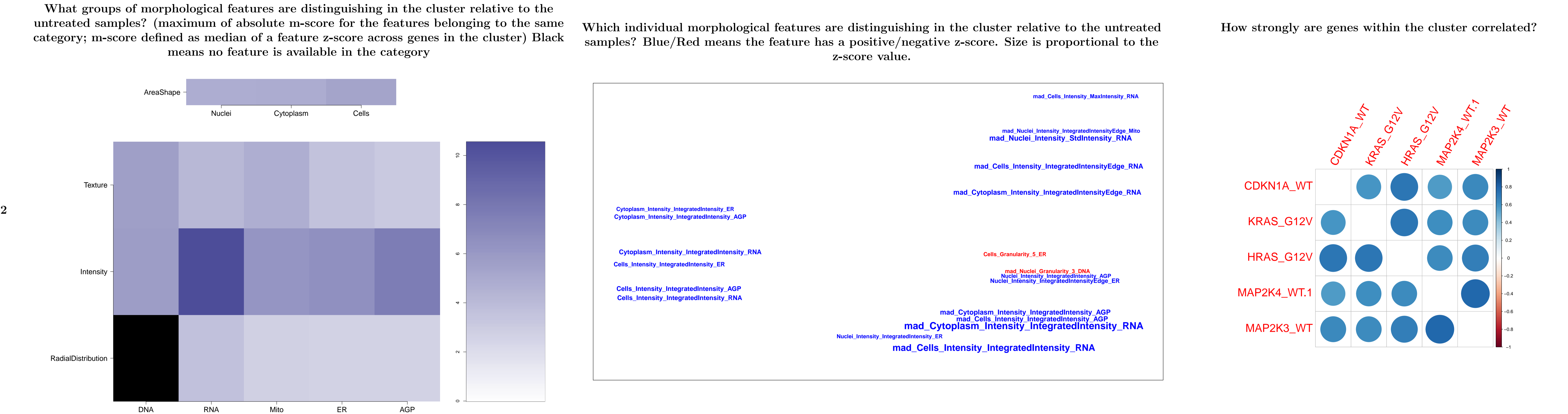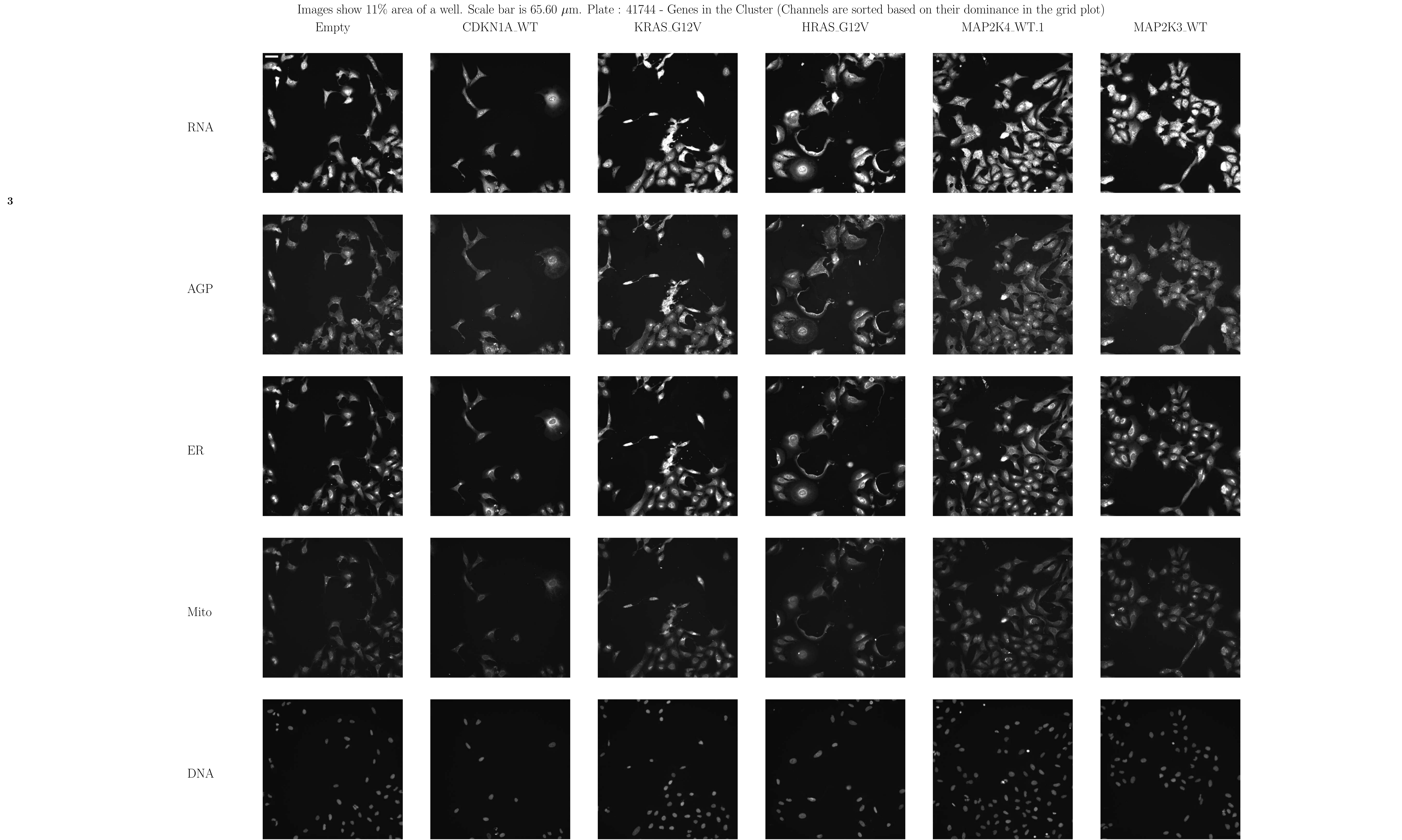

Supplement: Supplementary file 2. — The details of the contents have been described in Figure 5. DOI: http://dx.doi.org/10.7554/eLife.24060.017 [file elife-24060-supp2.zip › Supplementary file 2/type A/3A.pdf]

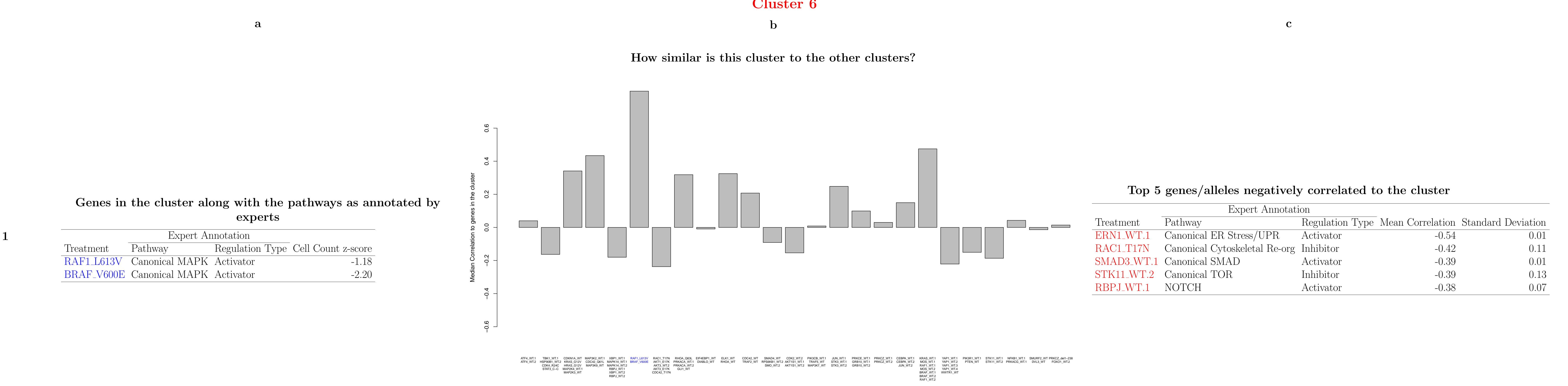

Supplement: Supplementary file 2. — The details of the contents have been described in Figure 5. DOI: http://dx.doi.org/10.7554/eLife.24060.017 [file elife-24060-supp2.zip › Supplementary file 2/type A/6A.pdf]
